# Supplementary material for: Phylogeography of Sub-Saharan Mitochondrial Lineages Outside Africa Highlights the Roles of the Holocene Climate Changes and the Atlantic Slave Trade
Source: Int J Mol Sci. 2022 Aug 16;23(16):9219. doi: 10.3390/ijms23169219 (PMC9408831; doi:10.3390/ijms23169219)
Supplement: Supplementary file 1 [file ijms-23-09219-s001.zip › Table S1.pdf]

Table S1. List of samples used in this study.

Sample ID	Haplogroup	Country
AF346967	L3e3b2	Unknown
AF346968	L1c1a1a1a	Unknown
AF346969	L1c1a2b	Unknown
AF346976	L2a1i1	Unknown
AF346977	L2a1a2	Unknown
AF346980	L3e1e1	Unknown
AF346985	L0a1a2	Unknown
AF346986	L1b1a3	Unknown
AF346987	L1c1d1	Unknown
AF346992	L1c2a1a	Unknown
AF346994	L3e2b7	Unknown
AF346995	L2c3a	Unknown
AF346996	L1c1a2b	Unknown
AF346997	L1c1a1a1a	Unknown
AF346998	L0a2b	Unknown
AF346999	L0a2b	Unknown
AF347000	L3h1a2a1	Unknown
AF347008	L0k1a1a	Unknown
AF347009	L0k1a1c	Unknown
AF347014	L3d1a1a	Unknown
AF347015	L3e2b1a1	Unknown
AF381981	L2c1a	Mauritania
AF381988	L0a1b1	Morocco
AF381991	L3b1a	Mauritania
AF381992	L1c3a	Mauritania
AF381994	L1b1a5	Mauritania
AF381998	L3d3b	Jordan
AM711903	L0a2b	Unknown
AM711904	L0d1c1a1a	Unknown
AY195766	L2b1a3	South Africa
AY195776	L2a1f	South Africa
AY195777	L0d2a1a	South Africa
AY195780	L0a1a2	South Africa
AY195782	L3d1d	South Africa
AY195783	L1b1a4a	South Africa
DQ304925	L2a1a1	USA
DQ304926	L2a1a	USA
DQ304927	L2a1a2c	USA
DQ304928	L2a1a	USA
DQ304929	L2a1e1	USA
DQ304930	L2a1e1	USA
DQ304931	L2a1e1	USA
DQ304932	L2a1a	USA
DQ304933	L2a1a1	USA
DQ304934	L2a1f1a	USA
DQ304935	L2a1f	USA
DQ304936	L2a1f	USA
DQ304937	L2a1f1	USA
DQ304938	L2a1m1a	USA
DQ304939	L2a1l1a	USA
DQ304940	L2a1m1	USA
DQ304941	L2a1n	USA
DQ304942	L2a1c4a	USA
DQ304943	L2a1c4a	USA
DQ304944	L2a1c	USA
DQ304945	L2a1e	USA
DQ304946	L2a1e1	USA
DQ304947	L2a1e1	USA
DQ304948	L2a1a3b	USA
DQ304949	L2a1c	USA
DQ304950	L2a1c4a1	USA
DQ304951	L2a1c4a1	USA
DQ304952	L2a1f	USA
DQ304953	L2a1f	USA
DQ304954	L2a1f	USA
DQ304955	L2a1f1	USA
DQ304956	L2a1f	USA
DQ304957	L2a1f2	USA
DQ304958	L2a1f1a	USA
DQ304959	L2a1f	USA
DQ304960	L2a1f1	USA
DQ304961	L2a1f1	USA
DQ304962	L2a1f	USA
DQ305001	L3e2b+152	USA
DQ305002	L3e2b+152	USA
DQ305003	L3e2b+152	USA
DQ305004	L3e2b+152	USA
DQ305005	L3e2b+152	USA
DQ305006	L3e2b+152	USA
DQ305007	L3e2b+152	USA
DQ305008	L3e2b3	USA
DQ305009	L3e2b3	USA
DQ305010	L3e3b	USA
DQ305011	L3e3b	USA
DQ305012	L3e3a	USA
DQ305013	L3e3b1	USA
DQ305014	L3e3a	USA
DQ305015	L3e3b	USA
DQ305016	L3e3b	USA

DQ305017	L3e3b1	USA
DQ305018	L3e2b8	USA
DQ305019	L3e2b8	USA
DQ305020	L3e2b8	USA
DQ305021	L3e2b1a1	USA
DQ305022	L3e2b1	USA
DQ305023	L3e2b1a1	USA
DQ305024	L3e2a1b1	USA
DQ305025	L3e2a1b3	USA
DQ305026	L3e2a1a	USA
DQ305027	L3e2a1a	USA
DQ305028	L3e2a1b1	USA
DQ305029	L3e2a1b	USA
DQ305030	L3e2a1b1	USA
DQ305031	L3e2a1b1	USA
DQ305032	L3f1b1a	USA
DQ305033	L3f1b1a	USA
DQ305034	L3f1b1a	USA
DQ305035	L3f1b1a	USA
DQ305036	L3f1b1a	USA
DQ341058	L0a2a2a	Dominican Republic
DQ341059	L1c2b1a'b	Ethiopia
EF184599	L0f2a	Tanzania
EF184600	L0f	Tanzania
EF184601	L0a1c1	Tanzania
EF184602	L0a2	Tanzania
EF184603	L0a2a1a	Tanzania
EF184604	L0a2	Tanzania
EF184605	L0a2a2a	Tanzania
EF184606	L0a2	Tanzania
EF184607	L0a2	Tanzania
EF184608	L0a2	Tanzania
EF184609	L0k1a1	South Africa
EF184610	L0k1a1b	South Africa
EF184611	L0k1a1a	South Africa
EF184612	L1c3b1a	Tanzania
EF184613	L1c1a2b	Cameroon
EF184614	L1c1a2b	Cameroon
EF184615	L1c1a2b	Cameroon
EF184616	L1c1a1a1a	Cameroon
EF184617	L2a1c5	Tanzania
EF184618	L2a1+143	Tanzania
EF184619	L2a1+143	Tanzania
EF184620	L2a1+143	Tanzania
EF184621	L2d1a	Tanzania
EF184622	L3h1a2	Tanzania
EF184623	L3d1a1a	Tanzania
EF184624	L3h1a2	Tanzania
EF184625	L3a1a	Tanzania
EF184627	L4b2a2	Tanzania
EF184628	L3d1a1a1	Tanzania
EF184629	L4b2a1	Tanzania
EF184630	L3a1a	Tanzania
EF184631	L3h1a2a1	Tanzania
EF184632	L3h1a2a1	Tanzania
EF184633	L4b2a2c	South Africa
EF184639	L4b2a2	Tanzania
EF184640	L4b2a2b	Tanzania
EF184641	L3d1a1a	Tanzania
EF556166	L3h1a2a1	Israel
EU092677	L3e3a	Israel
EU092678	L4a1a	Israel
EU092679	L2a1+143+16189	Israel
EU092680	L3f1b+16292	Israel
EU092681	L3e1b1	Israel
EU092682	L3b1b1	Israel
EU092683	L2a1c+16129	Israel
EU092684	L3x2a1a	Israel
EU092685	L3e1	Israel
EU092686	L6b	Israel
EU092687	L2a1l2a	Israel
EU092688	L0a1b1a1	Mozambique
EU092689	L1c3b1b	Mozambique
EU092690	L2a1b1a	Mozambique
EU092691	L2a1a2	Mozambique
EU092692	L2b2	Mozambique
EU092693	L3e1d1a	Mozambique
EU092694	L3b1a11	Mozambique
EU092695	L3e4a	Mozambique
EU092696	L3f1b1a	Mozambique
EU092697	L2c2	Mozambique
EU092698	L3e1b2	Mozambique
EU092699	L5a2	Mozambique
EU092700	L0d2a1	Mozambique
EU092701	L0a2a2a	Mozambique
EU092702	L3d1a1a	Mozambique
EU092703	L1c3a	Mozambique
EU092704	L3f1b4c	Mozambique
EU092705	L2a1b1a	Mozambique
EU092706	L3e3a	Mozambique
EU092707	L1c2a1a	Mozambique
EU092708	L0d2c1	Mozambique

EU092709	L3e1a3a	Mozambique
EU092710	L2c2b1b	Netherlands
EU092711	L2a1a3	Portugal
EU092712	L1c2b1c	Portugal
EU092713	L1b1a3a	Portugal
EU092714	L0a1a1	Guinea-Bissau
EU092753	L3h1a2b	Lebanon
EU092754	L2c2	Lebanon
EU092755	L1b1a15	Jordan
EU092756	L2a1j	Jordan
EU092757	L3e1	Jordan
EU092758	L3f1b2a	Jordan
EU092759	L3b1a4	Jordan
EU092760	L0a1c1	Iran
EU092761	L2a1b	Egypt
EU092762	L3d1c	Egypt
EU092763	L0a1a+200	Egypt
EU092764	L0a1a	Egypt
EU092765	L2a1d1	Egypt
EU092766	L2b1	Egypt
EU092767	L3b1a+@16124	Egypt
EU092768	L3b1a+@16124	Egypt
EU092769	L3e2a	Egypt
EU092770	L3f2b	Egypt
EU092771	L3f1b+16292	Egypt
EU092772	L3h1a2b	Egypt
EU092773	L6a	Egypt
EU092774	L5b1b	Egypt
EU092775	L1b1a2a	Egypt
EU092776	L3e5	Egypt
EU092777	L3e2b2	Egypt
EU092778	L2a1a2a1a	Egypt
EU092779	L3b1a1a	Kuwait
EU092780	L4b2a2b	Kuwait
EU092781	L5a1a	Kuwait
EU092782	L2a1+143+16189	Oman
EU092783	L2a1f	Oman
EU092784	L3e2b2	Oman
EU092785	L3e2b2	Oman
EU092786	L0f2b	Oman
EU092787	L0a2a2a	Oman
EU092788	L3i2	Oman
EU092789	L3e3a	Oman
EU092790	L3e1a3a	Oman
EU092829	L2a1a2a1a	Tunisia
EU092830	L3d1c1	Tunisia
EU092831	L0d1c3	South Africa
EU092832	L0d1c1a1b	South Africa
EU092833	L0d1a1b	South Africa
EU092834	L0d1c2	South Africa
EU092835	L0d2a1	South Africa
EU092836	L1c1d1	South Africa
EU092837	L0k1a1	South Africa
EU092838	L4b2a2c	South Africa
EU092839	L0d2a1a	South Africa
EU092840	L0d2b2	South Africa
EU092841	L0d1b2b2a	South Africa
EU092842	L0d3b1	South Africa
EU092843	L0d1c1a1a	South Africa
EU092844	L0d2c1a1	South Africa
EU092845	L0d2c1a1	South Africa
EU092846	L0d2a1a	South Africa
EU092847	L3e1a2	South Africa
EU092848	L1c2a3	South Africa
EU092849	L1c2b1b	South Africa
EU092850	L1c2a3	South Africa
EU092851	L3d3a1a	South Africa
EU092852	L1b1a10b	South Africa
EU092853	L0k1a2	South Africa
EU092854	L2b1a3	South Africa
EU092855	L0k1a2	South Africa
EU092856	L0d1c1a1a	South Africa
EU092857	L3f1b1a1	South Africa
EU092858	L0a1b1a1	South Africa
EU092859	L0d1c1a1b	South Africa
EU092860	L0d1c1a1b	South Africa
EU092861	L0a2a1b	South Africa
EU092862	L3e1d1	South Africa
EU092863	L0d1a1a3	South Africa
EU092864	L1c2a2	South Africa
EU092865	L3f1b4a	South Africa
EU092866	L1c3a	South Africa
EU092905	L2a1a3a	Chad
EU092906	L0a4	Chad
EU092907	L3e3a	Chad
EU092908	L3d1a1a	Kenya
EU092909	L0a1b1a1	Kenya
EU092910	L2a1b1a	Kenya
EU092911	L0a2a1a2	Kenya
EU092912	L3f1b4a1	Kenya
EU092913	L0a2d	Kenya
EU092914	L2a1h	Kenya

EU092915	L3e1e1	Kenya
EU092916	L2a1a	Kenya
EU092917	L3e2b	Kenya
EU092918	L3b1a+152	Kenya
EU092919	L2a1b1a	Jordan
EU092920	L3x2a1a	Jordan
EU092921	L0d3a	Kuwait
EU092922	L2a1+143+@16309	Kuwait
EU092923	L3i1b	Kuwait
EU092924	L6a	Saudi Arabia
EU092925	L0a2a2a	Yemen
EU092926	L3e2a2	Yemen
EU092927	L2a1d1	Oman
EU092928	L1b1a5	Tunisia
EU092929	L1b1a5	Egypt
EU092930	L3b1a1a	Cyprus
EU092931	L3b1a1a	Cyprus
EU092932	L3d1a1a1	Pakistan
EU092933	L2a1a2	Pakistan
EU092934	L5b1a	Pakistan
EU092935	L4a2	Pakistan
EU092936	L0b	Ethiopia
EU092937	L2a1m1	Ethiopia
EU092938	L4b2a2a	Ethiopia
EU092939	L2a1d1	Ethiopia
EU092940	L1b1a2a	Ethiopia
EU092941	L3a2a	Ethiopia
EU092942	L4b2a1	Ethiopia
EU273484	L1c1a1a2	Cameroon
EU273485	L1c1a1a1b1	Cameroon
EU273486	L1c1d1	Gabon
EU273487	L1c1a2b	Gabon
EU273488	L1c3b1a	Gabon
EU273489	L1c6	Gabon
EU273490	L1c1a1a1a	Gabon
EU273491	L1c1a1b	Gabon
EU273492	L1c1a1a1b	Gabon
EU273493	L1c3c	Gabon
EU273494	L1c1a1a1b	Gabon
EU273495	L1c1a1a1b	Gabon
EU273496	L1c1a2a1	Gabon
EU273497	L1c1a1a1b	Gabon
EU273498	L1c1a1a1a	Gabon
EU273499	L1c1b1	Gabon
EU273500	L1c1a1a2	Cameroon
EU273501	L1c2a2	Cameroon

EU273502	L1c4a	Gabon
EU547188	L2a1l2a	Poland
EU564850	L2a1l2a1	unknown
EU597489	L1c1a2a1	Congo
EU597490	L3b1a1a	Kenya
EU597491	L2a1+143+16189	Israel
EU597500	L3d5a	Nigeria
EU597501	L1c1a1a1b1	Congo
EU597502	L0d1b2b2b1	Africa
EU597512	L3d2b	Nigeria
EU597513	L1c1a1a1b	Congo
EU597514	L0d1a1a1	Africa
EU597525	L2a2b1a	Congo
EU597526	L3d3a1a	South Africa
EU597537	L0a2b	Africa
EU597549	L2a2b1a	Congo
EU597561	L2a1f3	Kenya
EU597570	L2d+16129	Latin America
EU597572	L1c2a1a	Congo
EU935434	L0a1b1a	Egypt
FJ625855	L3f3a	Chad
FJ625856	L3f3	Chad
FJ625857	L3f3b	Chad
FJ625858	L3f3b	Chad
FJ625859	L3f3b	Chad
FJ625860	L3f1b+16292	Chad
FJ713601	L1c1d1	Unknown
FJ769771	L2a1l1a	Bahamas
GU056815	L3e1	USA
GU455415	L3f1b5	Morocco
GU455416	L3e2b	Morocco
GU455417	L3d4	Morocco
GU455418	L3h1b2	Morocco
GU455419	L3f	Morocco
GU455420	L3d4	Morocco
GU455421	L3b1	Morocco
GU455422	L3f1b5	Morocco
HG00554	L1b1a3	Puerto Rico
HG00740	L1b1a7a	Puerto Rico
HG01063	L3e2b+152	Puerto Rico
HG01073	L3e1e2	Puerto Rico
HG01080	L1c3b1a	Puerto Rico
HG01095	L3e1e2	Puerto Rico
HG01108	L0a1a2	Puerto Rico
HG01170	L3e1e2	Puerto Rico
HG01176	L1b1a3	Puerto Rico

HG01188	L2b1a3	Puerto Rico
HG01286	L2a1l1b	Puerto Rico
HG01323	L3e1e2	Puerto Rico
HG01326	L3e1e2	Puerto Rico
HG01362	L3b1a+@16124	Colombia
HG01363	L2a1a2c	Colombia
HG01365	L3e1a	Colombia
HG01378	L1c3b1	Colombia
HG01389	L1c3a1b	Colombia
HG01403	L2b1a	Puerto Rico
HG01414	L3e2b3	Puerto Rico
HG02585	L3d2b	Gambia
HG02586	L3d1b1b	Gambia
HG02588	L2a1a	Gambia
HG02589	L1b1a	Gambia
HG02594	L3b1a	Gambia
HG02595	L2b	Gambia
HG02610	L3d1	Gambia
HG02611	L1c1c	Gambia
HG02613	L3b1	Gambia
HG02614	L3h1b2	Gambia
HG02620	L2a1l2	Gambia
HG02623	L3b1	Gambia
HG02624	L2c	Gambia
HG02628	L3d1b1	Gambia
HG02629	L2a1l2	Gambia
HG02634	L3b2b	Gambia
HG02635	L1b1a14	Gambia
HG02642	L2c	Gambia
HG02643	L2c5	Gambia
HG02645	L2b	Gambia
HG02646	L1b1a+189	Gambia
HG02666	L3e4a	Gambia
HG02667	L2c5	Gambia
HG02675	L3d3a	Gambia
HG02676	L2a1a3	Gambia
HG02678	L1b2	Gambia
HG02679	L2a1c	Gambia
HG02702	L3d1b1	Gambia
HG02703	L2c	Gambia
HG02715	L2c5	Gambia
HG02716	L2a1l2	Gambia
HG02721	L3b1a9a	Gambia
HG02722	L3b2a	Gambia
HG02756	L2c4	Gambia
HG02757	L2c4	Gambia

HG02759	L2c1	Gambia
HG02760	L2b1a	Gambia
HG02768	L3d1b1	Gambia
HG02922	L2a1a1	Nigeria
HG02923	L3f1b1a	Nigeria
HG02938	L3e2a	Nigeria
HG02941	L2a1a2a1a	Nigeria
HG02943	L3e3b	Nigeria
HG02944	L3e2a1b1	Nigeria
HG02946	L3f1b3	Nigeria
HG02947	L3e1a3b	Nigeria
HG02952	L2b1b	Nigeria
HG02953	L3d2b	Nigeria
HG02968	L3e2b+152	Nigeria
HG02970	L2d1a	Nigeria
HG02971	L0a1a2	Nigeria
HG02973	L3e2b1a2	Nigeria
HG02974	L1b1a3	Nigeria
HG02976	L3e1a	Nigeria
HG02977	L2a1a3c	Nigeria
HG02979	L2a1f	Nigeria
HG02981	L0a1a2	Nigeria
HG02982	L2b1a	Gambia
HG02983	L1c3a1a	Gambia
HG03024	L3h1b2	Gambia
HG03025	L3e4a1	Gambia
HG03027	L3e2a3	Gambia
HG03039	L3b1a+@16124	Gambia
HG03040	L3d1b1	Gambia
HG03045	L3h1b2	Gambia
HG03046	L2b1a2	Gambia
HG03048	L2c1a	Gambia
HG03049	L2c	Gambia
HG03052	L1b1a4	Sierra Leone
HG03054	L3b1	Sierra Leone
HG03055	L3b1	Sierra Leone
HG03057	L1b1a	Sierra Leone
HG03058	L1b1a14	Sierra Leone
HG03061	L3e2b1a	Sierra Leone
HG03063	L0a1a+200	Sierra Leone
HG03064	L2a1i	Sierra Leone
HG03133	L1c2b1c	Nigeria
HG03135	L2a1f1	Nigeria
HG03136	L1b1a3	Nigeria
HG03139	L4b2b	Nigeria
HG03157	L2c2a1	Nigeria

HG03159	L2a1f	Nigeria
HG03160	L3e2b8	Nigeria
HG03162	L3f1b+16292+150	Nigeria
HG03163	L2a1f1	Nigeria
HG03166	L1b1a3b	Nigeria
HG03168	L1c2a1	Nigeria
HG03169	L2a1a3c	Nigeria
HG03172	L3f1b1a	Nigeria
HG03175	L3e2a1b1	Nigeria
HG03189	L4b2b	Nigeria
HG03190	L3e1a	Nigeria
HG03193	L3f1b1a	Nigeria
HG03195	L3e2b1	Nigeria
HG03196	L3e1	Nigeria
HG03198	L3d5a	Nigeria
HG03199	L2a1f	Nigeria
HG03202	L2a1b1	Nigeria
HG03209	L2e	Sierra Leone
HG03212	L3e4a1	Sierra Leone
HG03224	L3d2	Sierra Leone
HG03225	L2c	Sierra Leone
HG03240	L2e	Gambia
HG03241	L3d1a1b	Gambia
HG03246	L3b1a+@16124	Gambia
HG03247	L3d2a	Gambia
HG03259	L4b1a	Gambia
HG03265	L3d5a	Nigeria
HG03267	L0a1a2	Nigeria
HG03268	L0a1a2	Nigeria
HG03270	L1b1a18	Nigeria
HG03271	L1c2b1b	Nigeria
HG03279	L2d1	Nigeria
HG03280	L3e2b8	Nigeria
HG03439	L3f1b1	Sierra Leone
HG03442	L1b1a10	Sierra Leone
HG03445	L3d1'2'3'4'5'6	Sierra Leone
HG03446	L3b1a	Sierra Leone
HG03449	L2a1c2a	Sierra Leone
HG03451	L2c	Sierra Leone
HG03452	L2a1f	Sierra Leone
HG03455	L2c	Sierra Leone
HG03457	L2a1l1b	Sierra Leone
HG03458	L4b1a	Sierra Leone
HG03460	L2a1a1	Sierra Leone
HG03461	L2a1i	Sierra Leone
HG03464	L1c1c	Sierra Leone
HG03469	L1b1a14	Sierra Leone
HG03470	L1b1a14	Sierra Leone
HG03473	L2c	Sierra Leone
HG03476	L2c	Sierra Leone
HG03478	L3e'i'k'x	Sierra Leone
HG03484	L2c	Sierra Leone
HG03499	L1c2a2	Nigeria
HG03511	L3e2b	Nigeria
HG03514	L2a1f	Nigeria
HG03515	L2b1a3	Nigeria
HG03517	L0a1a2	Nigeria
HG03518	L3d1c1	Nigeria
HG03520	L3e1	Nigeria
HG03521	L3b1a10	Nigeria
HG03539	L2a1a3	Gambia
HG03547	L2a1l	Sierra Leone
HG03548	L2a1a1	Sierra Leone
HG03556	L3e4a1	Sierra Leone
HG03557	L2a1c3a	Sierra Leone
HG03558	L3h1b1a	Sierra Leone
HG03559	L3d1b1	Sierra Leone
HG03563	L2a1a	Sierra Leone
HG03565	L3b1a5	Sierra Leone
HG03567	L3d1b3a	Sierra Leone
HG03571	L2a1c3a	Sierra Leone
HM771145	L1c1a1a1b1	Cameroon
HM771146	L1c1a1a1a	Congo
HM771147	L1c1a1a2	Cameroon
HM771148	L1c1a2a2	Central African Republic
HM771149	L1c1a2a1	Central African Republic
HM771150	L1c1a2b	Central African Republic
HM771151	L1c1a1a1a	Central African Republic
HM771152	L1c1a1a1a	Central African Republic
HM771153	L1c1a1a1a	Central African Republic
HM771154	L1c1a1a1b1	Central African Republic
HM771155	L1c1a1a1b1	Central African Republic
HM771156	L1c1a1a1a	Central African Republic
HM771157	L1c4b	Central African Republic
HM771158	L1c4b	Central African Republic
HM771159	L1c4b	Central African Republic

HM771160	L0a2a1	Central African Republic
HM771161	L0a2a1	Central African Republic
HM771162	L1b2a	Gabon
HM771163	L1b1a3	Gabon
HM771164	L1c1a2a2	Gabon
HM771165	L1c1a1a1b1	Gabon
HM771166	L1c1d	Gabon
HM771167	L1c3b1a	Gabon
HM771168	L2a1a3c	Congo
HM771169	L2a1c2a	Congo
HM771170	L3d2a	Gabon
HM771171	L3e1d	Gabon
HM771172	L3e1f1a	Gabon
HM771173	L3e1a3a	Gabon
HM771174	L3e2a1b2	Gabon
HM771175	L3e2a1b2	Gabon
HM771176	L3e2a1b2	Gabon
HM771177	L3e2b+152	Gabon
HM771178	L1c1a2b	Central African Republic
HM771179	L1c1a2a2	Central African Republic
HM771180	L1c1a2a2	Central African Republic
HM771181	L1c1a1a1a	Central African Republic
HM771182	L1c1a1a1b	Central African Republic
HM771221	L1c2b2	Gabon
HM771222	L1c2b1a	Gabon
HM771223	L1c2b2	Gabon
HM771224	L2a1c	Congo
HM771225	L2a1c5	Congo
HM771226	L2b1b	Congo
HM771227	L3d1a1a	Gabon
HM771228	L3d1a2	Gabon
HM771229	L3e2b1	Gabon
HM771230	L3e2b1	Gabon
HM771231	L3e3b1	Gabon
HM771232	L3e3b2	Gabon
HM771233	L4b2b1	Unknown
HQ384199	L2a5	Spain
HQ425328	L3d2b	USA
HQ425645	L2a1a2c	USA
HQ610935	L4a	Unknown
HQ675033	L3x2b	Spain
JF509360	L1c3b1	Western Sahara
JF682349	L3b1a1a	USA

JF812599	L1c4b	USA
JN204423	L2a1l2a	Poland
JN214430	L1b1a8	Spain
JN214431	L1b1a8	Spain
JN214432	L2a1c4	Spain
JN214433	L2c1a	Spain
JN214434	L3f1b+16292	Spain
JN214435	L1b1a8	Spain
JN214436	L2a1c6	Spain
JN214437	L1b1a12a	Spain
JN214438	L1b1a	Spain
JN214440	L2a1c3a	Spain
JN214441	L1b1a8	Spain
JN214442	L1b1a6	Spain
JN214443	L2b3b	Spain
JN214444	L1b1a5	Spain
JN214446	L3x2b	Spain
JN214447	L1b1a16	Spain
JN655773	L3x1a2	Ethiopia
JN655774	L3a1b	Ethiopia
JN655775	L3h1b1a	Ethiopia
JN655776	L3x2a	Ethiopia
JN655777	L3f1b+16292	Ethiopia
JN655778	L3i2	Ethiopia
JN655779	L3x1a2	Ethiopia
JN655780	L3i1a	Ethiopia
JN655781	L3x1b	Ethiopia
JN655782	L3x1b	Ethiopia
JN655783	L3f1b+16292	Ethiopia
JN655784	L3f	Ethiopia
JN655785	L3i2	Ethiopia
JN655786	L3x1+16311	Ethiopia
JN655787	L3i2	Ethiopia
JN655788	L3h1a2b	Ethiopia
JN655789	L3k1	Chad
JN655790	L3b1a+152	Chad
JN655791	L3b1a9	Chad
JN655792	L3b1a+@16124	Chad
JN655793	L3d2b	Chad
JN655794	L3e1b1	Chad
JN655795	L3e2b6	Chad
JN655796	L3e3b	Chad
JN655797	L3d3b	Chad
JN655798	L3e5a1a	Chad
JN655799	L3h1b1a	Chad
JN655800	L3d1a1a	Somalia

JN655801	L3h2	Somalia	JQ044833	L2a1c4a	Burkina Faso
JN655802	L3x2a	Somalia	JQ044834	L4b1a	Burkina Faso
JN655803	L3a+709	Somalia	JQ044873	L3d1b2	Burkina Faso
JN655804	L3i2	Somalia	JQ044874	L0a1a2	Burkina Faso
JN655805	L3a2a	Somalia	JQ044875	L1b1a3a1	Burkina Faso
JN655806	L3b1a+@16124	Somalia	JQ044876	L1b1a9	Burkina Faso
JN655807	L3e1a1a	Somalia	JQ044877	L2a1a	Burkina Faso
JN655808	L3d1a1	Somalia	JQ044878	L2c2b2	Burkina Faso
JN655809	L3f1a1	Somalia	JQ044879	L2a1a2	Burkina Faso
JN655810	L3x1b	Somalia	JQ044880	L3f1b+16292	Burkina Faso
JQ044795	L1c3b2	Burkina Faso	JQ044881	L2a1i1	Burkina Faso
JQ044796	L3b1a6	Burkina Faso	JQ044882	L2c2	Burkina Faso
JQ044797	L2b2	Burkina Faso	JQ044883	L2a1f	Burkina Faso
JQ044798	L3b1a+152	Burkina Faso	JQ044884	L2a1a2	Burkina Faso
JQ044799	L2a1c1a1	Burkina Faso	JQ044885	L2a1a	Burkina Faso
JQ044800	L2b1b	Burkina Faso	JQ044886	L1c3b2	Burkina Faso
JQ044801	L3e2a1b1	Burkina Faso	JQ044887	L2c1a	Burkina Faso
JQ044802	L2a1c2	Burkina Faso	JQ044888	L2a1a1	Burkina Faso
JQ044804	L2a1m	Burkina Faso	JQ044889	L1b1a	Burkina Faso
JQ044805	L3b1a	Burkina Faso	JQ044890	L2b1a2	Burkina Faso
JQ044806	L1c3a	Burkina Faso	JQ044891	L3d1b3a	Burkina Faso
JQ044808	L1b1a17	Burkina Faso	JQ044892	L2a1a3	Burkina Faso
JQ044809	L2a1a2	Burkina Faso	JQ044893	L0a1a1	Burkina Faso
JQ044810	L2c3	Burkina Faso	JQ044894	L3f1b1	Burkina Faso
JQ044811	L4b1a	Burkina Faso	JQ044895	L3f1b1a	Burkina Faso
JQ044812	L2a1a2	Burkina Faso	JQ044897	L2a1l1a2	Burkina Faso
JQ044813	L2a1a3a	Burkina Faso	JQ044898	L1b1a9	Burkina Faso
JQ044814	L3d1b3a	Burkina Faso	JQ044899	L3e2b	Burkina Faso
JQ044815	L3d1a1	Burkina Faso	JQ044900	L3f1b+16292	Burkina Faso
JQ044816	L2e1	Burkina Faso	JQ044901	L2c1a	Burkina Faso
JQ044817	L2a1l1a1	Burkina Faso	JQ044902	L3b1a6	Burkina Faso
JQ044818	L2a1a	Burkina Faso	JQ044903	L0a1a+200	Burkina Faso
JQ044819	L2a1a3b	Burkina Faso	JQ044905	L2a1i	Burkina Faso
JQ044820	L3b1a7a	Burkina Faso	JQ044906	L3e2b+152	Burkina Faso
JQ044821	L2a1e1	Burkina Faso	JQ044907	L1c3a	Burkina Faso
JQ044822	L2a1p	Burkina Faso	JQ044908	L2a1m1a	Burkina Faso
JQ044823	L2c4	Burkina Faso	JQ044909	L2a1c1	Burkina Faso
JQ044824	L3d1c1	Burkina Faso	JQ044910	L2b2	Burkina Faso
JQ044825	L1b1a15	Burkina Faso	JQ044911	L2a1a	Burkina Faso
JQ044826	L3b'f	Burkina Faso	JQ044912	L2a1f	Burkina Faso
JQ044827	L3e2b	Burkina Faso	JQ044955	L2a1l1b	Burkina Faso
JQ044828	L2a1c2a	Burkina Faso	JQ044956	L2a1l1	Burkina Faso
JQ044829	L3d1d	Burkina Faso	JQ044957	L2a1c4a	Burkina Faso
JQ044830	L3e	Burkina Faso	JQ044958	L2a1i	Burkina Faso
JQ044831	L3f1b1	Burkina Faso	JQ044959	L3d4	Burkina Faso
JQ044832	L3e2b	Burkina Faso	JQ044960	L3b1a+152	Burkina Faso

JQ044961	L2a1a	Burkina Faso	JQ045047	L2a1l3	Burkina Faso
JQ044962	L2a1c2	Burkina Faso	JQ045048	L2a1a2	Burkina Faso
JQ044963	L3e2b2	Burkina Faso	JQ045049	L2e1	Burkina Faso
JQ044964	L3e3b3	Burkina Faso	JQ045050	L2d	Burkina Faso
JQ044965	L3b1a10	Burkina Faso	JQ045051	L3d1b3	Burkina Faso
JQ044966	L2a1l1a	Burkina Faso	JQ045052	L3f1b1a	Burkina Faso
JQ044967	L1c3b1b	Burkina Faso	JQ045053	L0a1a2	Burkina Faso
JQ044968	L2a1a2a1	Burkina Faso	JQ045054	L2a1f	Burkina Faso
JQ044969	L2a1c2a	Burkina Faso	JQ045055	L1b1a10	Burkina Faso
JQ044970	L2a1e1	Burkina Faso	JQ045056	L2a1f	Burkina Faso
JQ044971	L2c3	Burkina Faso	JQ045057	L3d2b	Burkina Faso
JQ044972	L3e2b5	Burkina Faso	JQ045058	L3b1a6	Burkina Faso
JQ044973	L2a1c+16086	Burkina Faso	JQ045059	L3d1b	Burkina Faso
JQ044974	L2a1c1	Burkina Faso	JQ045060	L2a1a	Burkina Faso
JQ044975	L2a1b2	Burkina Faso	JQ045061	L2a1f	Burkina Faso
JQ044976	L2c	Burkina Faso	JQ045062	L2a1	Burkina Faso
JQ044977	L2a1a1	Burkina Faso	JQ045063	L2a1c3b2	Burkina Faso
JQ044978	L2a1l2	Burkina Faso	JQ045064	L2a1c1	Burkina Faso
JQ044979	L3f1b+16292+150	Burkina Faso	JQ045065	L3d1b3	Burkina Faso
JQ044980	L1b1a9	Burkina Faso	JQ045066	L2a1a1	Burkina Faso
JQ044981	L2a1n	Burkina Faso	JQ045067	L2a1c4a	Burkina Faso
JQ044982	L3e3b	Burkina Faso	JQ045068	L2c1a	Burkina Faso
JQ044983	L2a1a	Burkina Faso	JQ045069	L2d+16129	Burkina Faso
JQ044984	L2c5	Burkina Faso	JQ045070	L3e1g	Burkina Faso
JQ044985	L3e2b1a	Burkina Faso	JQ045109	L2c3a	Burkina Faso
JQ044986	L3b1a5	Burkina Faso	JQ045110	L2a1c3b1	Burkina Faso
JQ044987	L2a1a2	Burkina Faso	JQ045111	L1b1a	Burkina Faso
JQ044988	L3d1c1	Burkina Faso	JQ045112	L3d1b	Burkina Faso
JQ044989	L2c1	Burkina Faso	JQ045113	L1b1a4a	Burkina Faso
JQ044990	L3h1b2	Burkina Faso	JQ045114	L1b1a14	Burkina Faso
JQ044991	L3e2a	Burkina Faso	JQ045116	L2a1c3b1	Burkina Faso
JQ044992	L2a1a	Burkina Faso	JQ045117	L3d1d	Burkina Faso
JQ045033	L3e2a1b3	Burkina Faso	JQ045118	L3e4a1	Burkina Faso
JQ045034	L3b1a6	Burkina Faso	JQ045119	L1b1a	Burkina Faso
JQ045035	L2a1c4a	Burkina Faso	JQ045120	L3b1a	Burkina Faso
JQ045036	L1b1a	Burkina Faso	JQ045121	L1b1a17	Burkina Faso
JQ045037	L2b1a2	Burkina Faso	JQ045122	L2a1c3b1	Burkina Faso
JQ045038	L1b3	Burkina Faso	JQ045123	L3b1a	Burkina Faso
JQ045039	L2a1f2	Burkina Faso	JQ045124	L2a1c1	Burkina Faso
JQ045040	L2a1m	Burkina Faso	JQ045125	L3e4a1	Burkina Faso
JQ045041	L3b1a+152	Burkina Faso	JQ412577	L2a1c1a2	unknown
JQ045042	L2c2	Burkina Faso	JQ701814	L2a1f1	USA
JQ045043	L2b2	Burkina Faso	JQ701823	L1b1a3b	USA
JQ045044	L2d1	Burkina Faso	JQ701829	L3e2b1a2	Africa
JQ045045	L2a1c4a	Burkina Faso	JQ701833	L2b1a3	African European
JQ045046	L2a1f3	Burkina Faso	JQ701901	L1c1c	Unknown

JQ701914	L2a1m1a	Ireland	JQ705587	L1b1a15a	USA
JQ701926	L2a1c4a	unknown	JQ705589	L2a1l2a1	Romania
JQ701954	L2c	unknown	JQ705596	L3e5b	Unknown
JQ702015	L2a1l2a1	Poland	JQ705597	L1c2b1c	Unknown
JQ702047	L3k1	Unknown	JQ705606	L1b1a18	Unknown
JQ702115	L2c4	unknown	JQ705626	L2c3	unknown
JQ702123	L2b3a	Hawaii	JQ705650	L1c2b1b1	Mexico
JQ702169	L2c	unknown	JQ705669	L1b1a3a	Unknown
JQ702179	L3e3b	Unknown	JX303768	L1c1a2	Zambia
JQ702227	L0a1a2	Unknown	JX303769	L1b1a10b	Zambia
JQ702241	L3b1a10	Unknown	JX303770	L1b2a	Zambia
JQ702261	L2a1c1a2	unknown	JX303771	L1b1a3	Zambia
JQ702307	L2a1c	unknown	JX303772	L0a2a2a2	Zambia
JQ702326	L0a1b1a1	Unknown	JX303773	L1c2b1b1	Zambia
JQ702381	L3b1a	Africa	JX303774	L3f1b1a1	Zambia
JQ702420	L3d1b2	Unknown	JX303775	L1c2a1a	Zambia
JQ704982	L3e2a1a	Unknown	JX303776	L3e1	Zambia
JQ705001	L2a1c5	unknown	JX303777	L1b1a	Zambia
JQ705012	L3e2b	Unknown	JX303778	L0a2a2a	Zambia
JQ705019	L3d2b	Unknown	JX303779	L1b1a	Zambia
JQ705046	L2a1c1a1	Ghana	JX303780	L2a1f1	Zambia
JQ705049	L2a1l2a	Poland	JX303781	L3e1e1	Zambia
JQ705055	L2a1a2a1a	Mozambique	JX303782	L1c2a3a	Zambia
JQ705077	L3d1b2	Unknown	JX303783	L1c2a1a	Zambia
JQ705087	L2a1a	unknown	JX303784	L0a2a1b	Zambia
JQ705109	L0a2a2a	Unknown	JX303785	L1c3b1a	Zambia
JQ705115	L1b1a4	USA	JX303786	L0a2a2a2	Zambia
JQ705120	L2c	unknown	JX303787	L1b1a3	Zambia
JQ705136	L3e2a1b1	Unknown	JX303788	L0d2b2	Zambia
JQ705137	L3h1b1a	Unknown	JX303789	L3e2b1a2	Zambia
JQ705145	L2a1c3a1	USA	JX303790	L1c2b1b1	Zambia
JQ705150	L2a1f	unknown	JX303791	L0d1c2a	Zambia
JQ705185	L2a1l2a	Russia	JX303792	L2c2a1	Zambia
JQ705249	L1b1a6	Unknown	JX303793	L1c2a1a	Zambia
JQ705250	L2a1a2	unknown	JX303794	L1c2b1b1	Zambia
JQ705275	L1c1a	Puerto Rico	JX303795	L2a1d2	Zambia
JQ705285	L3e4a	Unknown	JX303796	L0a1b1a1	Zambia
JQ705310	L3k1	Unknown	JX303797	L1c5	Zambia
JQ705320	L3e4a1	Unknown	JX303798	L2a1g	Zambia
JQ705361	L3b1a	Unknown	JX303799	L3e3b2	Zambia
JQ705410	L3d2b	Unknown	JX303800	L1c2b1b1	Zambia
JQ705455	L2a1e1	unknown	JX303801	L1b1a10b	Zambia
JQ705478	L1b1a6	Unknown	JX303802	L3d3a1b	Zambia
JQ705521	L3e1f2	Unknown	JX303803	L3d3a1b	Zambia
JQ705529	L2a1a3a	unknown	JX303804	L3f1b1a1	Zambia
JQ705576	L1c2b1c	Unknown	JX303805	L2a1f3	Zambia

JX303844	L3e2b	Zambia	KC345764	L0d1c	Angola
JX303845	L1c2b2	Zambia	KC345765	L0d1b1b1	Angola
JX303846	L1b1a10b	Zambia	KC345766	L0d1b1b1	Angola
JX303847	L3e1d1	Zambia	KC345767	L0d1b1b1	Angola
JX303848	L1c2a1a	Zambia	KC345768	L0d1b1b1	Angola
JX303849	L3e1d1a	Zambia	KC345769	L0d1a1b1a	Angola
JX303850	L3e1d1	Zambia	KC345770	L0d1a1b1a	Angola
JX303851	L1c2b1b1	Zambia	KC345771	L0d1b1b1	Angola
JX303852	L2a1c1	Zambia	KC345772	L0d1b1b1	Angola
JX303853	L2a1i1	Zambia	KC345773	L0d1a1b1a	Angola
JX303854	L3e1a3a	Zambia	KC345774	L0d1b1b1	Angola
JX303855	L1c2a1a	Zambia	KC345775	L0d1a1b1a	Angola
JX303856	L0k2a1a	Zambia	KC345776	L0d1a1b1a	Angola
JX303857	L2a1i1	Zambia	KC345777	L0d2a1a	Angola
JX303858	L2a1b1a	Zambia	KC345778	L0d1c2	Angola
JX303859	L1b1a10b	Zambia	KC345779	L0d1c	Angola
JX303860	L1b1a10b	Zambia	KC345780	L0d1b1+@152	Angola
JX303861	L0k1b	Zambia	KC345781	L0d1b2b2	Angola
JX303862	L2a1d2	Zambia	KC345782	L0d1a1b1a	Angola
JX303863	L2c2a1	Zambia	KC345783	L0d1b1b1	Angola
JX303864	L3d3a1	Zambia	KC345784	L0d2a1a	Angola
JX303865	L0k1b	Zambia	KC345785	L0k1a2	Angola
JX303866	L1b1a10b	Zambia	KC345786	L0d1b2a	Botswana
JX303867	L0k1b	Zambia	KC345787	L0d1b2a1	Botswana
JX303868	L0k2a1a	Zambia	KC345788	L0d1b1a1	Botswana
JX303869	L0a1a2	Zambia	KC345789	L0d1b1a1	Botswana
JX303870	L2a1q	Zambia	KC345790	L0d1b1a1	Botswana
JX303871	L1c3a1b	Zambia	KC345791	L0d1a	Botswana
JX303872	L3e1a3a	Zambia	KC345792	L0d1c2	Botswana
JX303873	L2a1d2	Zambia	KC345793	L0d1b1a1	Botswana
JX303874	L2a1b1a	Zambia	KC345832	L0d1a1a1	Botswana
JX303875	L3e1d1	Zambia	KC345833	L0d1c1a2	Botswana
JX303876	L3b1a3	Zambia	KC345834	L0d1c1a1a2	Botswana
JX303877	L1c2a1a	Zambia	KC345835	L0d1c1a1a1	Botswana
JX303878	L3e1a2	Zambia	KC345836	L0d1c2a1	Botswana
JX303879	L1c2a1a	Zambia	KC345837	L0d1c1a2	Botswana
JX303880	L2a1b1a	Zambia	KC345838	L0d2b1a1a	Botswana
JX303881	L3e1a3a	Zambia	KC345839	L0d2b1a1a	Botswana
KC257337	L1c3b2	USA	KC345840	L0d2b1a1a	Botswana
KC257338	L1c3b2	USA	KC345841	L0d2b1a1a	Botswana
KC257339	L1c3b2	USA	KC345842	L0d2b1a1	Botswana
KC257340	L1c3b2	USA	KC345843	L0d2b1a1a	Botswana
KC257341	L1c3b2	USA	KC345844	L0d2b1a1	Botswana
KC257342	L1c3b2	USA	KC345845	L0d2b1a1a	Botswana
KC257343	L1c3b2	USA	KC345846	L0d2b1a1a	Botswana
KC257344	L1c3b2	USA	KC345847	L0d2b1a1a	Botswana

KC345848	L0d2b1a1a	Botswana	KC345932	L0d1c1a1a	Botswana
KC345849	L0d2b1a1a	Botswana	KC345933	L0d1c1a1	Botswana
KC345850	L0d2b1a1a	Botswana	KC345934	L0d1c1a1a	Botswana
KC345851	L0k1a2	Botswana	KC345935	L0d1c1a1a	Botswana
KC345852	L0d1c2a	Botswana	KC345936	L0d1c1a1b	Botswana
KC345853	L0d1c1a1a1	Botswana	KC345937	L0d1b2b1a	Botswana
KC345854	L0d1c1a2	Botswana	KC345938	L0d1c2a1	Botswana
KC345855	L0d1a1a1	Botswana	KC345939	L0d1c1a1a1	Botswana
KC345856	L0d1c1a1a1	Botswana	KC345940	L0d1c1a2	Botswana
KC345857	L0d1c1a2	Botswana	KC345941	L0d1c1a2	Botswana
KC345858	L0d1c1a1a1	Botswana	KC345942	L0d1c1a2	Botswana
KC345859	L0d1c1a1a1	Botswana	KC345943	L0d1b2b1a	Botswana
KC345860	L0d1c1a1a1	Botswana	KC345944	L0d1c3	Botswana
KC345861	L0d1c1a1a1	Botswana	KC345945	L0d1c1a1a1	Botswana
KC345862	L0d1c1a1a1	Botswana	KC345984	L0d1c2a	Botswana
KC345863	L0d1c1a1a1	Botswana	KC345985	L0d1c	Botswana
KC345864	L0d1a1a1	Botswana	KC345986	L0d1c2a	Botswana
KC345865	L0d2a1a1a	Botswana	KC345987	L0d2a1a1	Botswana
KC345866	L0d2a1a	Botswana	KC345988	L0d2a1a1a	Botswana
KC345867	L0d1b2b1a	Botswana	KC345989	L0d2a1a1	Botswana
KC345868	L0d1b2b2c1	Botswana	KC345990	L0d2a1b	Botswana
KC345869	L0d1b2a1	Botswana	KC345991	L0d2a1b	Botswana
KC345908	L0d1b2b1a	Botswana	KC345992	L0d2a1a1a	Botswana
KC345909	L0d1b1a1	Botswana	KC345993	L0d2a1a1a	Botswana
KC345910	L0d2a2	Botswana	KC345994	L0d1c1a1a2	Botswana
KC345911	L0d2a1a1	Botswana	KC345995	L0d1c1a2	Botswana
KC345912	L0d2b1a1a	Botswana	KC345996	L0d1c1a2	Botswana
KC345913	L0k1a1d	Botswana	KC345997	L0d1c1a1a	Botswana
KC345914	L0k1a1	Botswana	KC345998	L0d1c1a1a1	Botswana
KC345915	L0k1a1	Botswana	KC345999	L0d1c1a1a	Botswana
KC345916	L0k1a1d	Botswana	KC346000	L0d1c1a1a1	Botswana
KC345917	L0d1c	Botswana	KC346001	L0d1c1a2	Botswana
KC345918	L0d1b1a1	Botswana	KC346002	L0d1c1a1a1	Botswana
KC345919	L0d1c1a1a	Botswana	KC346003	L0d1c1a1a	Botswana
KC345920	L0d1c1	Botswana	KC346004	L0d2a1a1a	Botswana
KC345921	L0d1b2b2a	Botswana	KC346005	L0d2a1b	Botswana
KC345922	L0k2a1	Botswana	KC346006	L0d2a1a1	Botswana
KC345923	L0d1b2b1b	Botswana	KC346007	L0d2a1a1	Botswana
KC345924	L0d1b2b2	Botswana	KC346008	L0d2a1a1	Botswana
KC345925	L0d1b2a1	Botswana	KC346009	L0d2b1a1a	Botswana
KC345926	L0d1b2b1b	Botswana	KC346010	L0d2a1a1a	Botswana
KC345927	L0d2a1b	Botswana	KC346011	L0d2a1b	Botswana
KC345928	L0d2a1a3	Botswana	KC346012	L0d2a1a1	Botswana
KC345929	L0d2a1b	Botswana	KC346013	L0d1c1a1a2	Botswana
KC345930	L0d3b1	Botswana	KC346014	L0d1b2a1	Botswana
KC345931	L0d3b2	Botswana	KC346015	L0d1c2a1	Botswana

KC346016	L0d1b1a1	Botswana
KC346017	L0d1c2a1	Botswana
KC346018	L0d1c2a1	Botswana
KC346019	L0d1c1a1a	Botswana
KC346020	L0d2a1a1	Botswana
KC346021	L0d2a1a1	Botswana
KC346060	L0d2c2a1a	Botswana
KC346061	L0d2a1a1	Botswana
KC346062	L0d2c2a1a	Botswana
KC346063	L0k1a1d	Botswana
KC346064	L0d1b2b2a	Botswana
KC346065	L0k1a2	Botswana
KC346066	L0d1b2a1	Botswana
KC346067	L0d1b2a1	Botswana
KC346068	L0d1c1a1a	Botswana
KC346069	L0d1b2a1	Botswana
KC346070	L0d1b2a1	Botswana
KC346071	L0d2b1a1a	Botswana
KC346072	L0k1a3	Botswana
KC346073	L0k1a3	Botswana
KC346074	L0d1b1a	Botswana
KC346075	L0d1c1a1a2	Botswana
KC346076	L0d1c1a2	Botswana
KC346077	L0d1c1a2	Botswana
KC346078	L0d1b1a	Botswana
KC346079	L0d1c1a2	Botswana
KC346080	L0d1c1a1a1	Botswana
KC346081	L0d1c2a1	Botswana
KC346082	L0d1c1a2	Botswana
KC346083	L0k1a2	Botswana
KC346084	L0d1c1a2	Botswana
KC346085	L0d1c1a1b	Botswana
KC346086	L0d2c1a	Botswana
KC346087	L0d2a1a	Botswana
KC346088	L0d1c1a1a	Botswana
KC346089	L0d1c1a1a	Botswana
KC346090	L0d2a1	Botswana
KC346091	L0d2a1a3	Botswana
KC346092	L0d1a1c	Namibia
KC346093	L0d1b2b2c2	Namibia
KC346094	L0d1c1a1b	Namibia
KC346095	L0d1c1a1a	Namibia
KC346096	L0d1c1a1b	Namibia
KC346097	L0d1c1a1a	Namibia
KC346136	L0d1b2b2c1	Namibia
KC346137	L0d1c1a1b	Namibia
KC346138	L0d1b2b1b	Namibia
KC346139	L0d1b2b2c1	Namibia
KC346140	L0d1c1a1b	Namibia
KC346141	L0d1b2b2c2	Namibia
KC346142	L0d1c1a1b	Namibia
KC346143	L0d1b2b2c2	Namibia
KC346144	L0d1c1a	Namibia
KC346145	L0d2a1a1	Namibia
KC346146	L0d2c2	Namibia
KC346147	L0d2b1b	Namibia
KC346148	L0d3b1	Namibia
KC346149	L0k1a1	Namibia
KC346150	L0k1a1	Namibia
KC346151	L0d1b2b1b	Namibia
KC346152	L0d1b2a1	Namibia
KC346153	L0d1c1a1a	Namibia
KC346154	L0d2b1b	Namibia
KC346155	L0d2b1b	Namibia
KC346156	L0d2c1b	Namibia
KC346157	L0d2c1a	Namibia
KC346158	L0d2c1b	Namibia
KC346159	L0k1a1	Namibia
KC346160	L0k1a1c	Namibia
KC346161	L0k1a1	Namibia
KC346162	L0k1a1a	Namibia
KC346163	L0d1b2b2b1	Namibia
KC346164	L0d1b2b1b	Namibia
KC346165	L0d1b2b2b1	Namibia
KC346166	L0d1c1a1b	Namibia
KC346167	L0d2b1b	Namibia
KC346168	L0d2a1a	Namibia
KC346169	L0d2b1b	Namibia
KC346170	L0d1b2b2c1	Namibia
KC346171	L0d1b2a1	Namibia
KC346172	L0d1c1a1b	Namibia
KC346173	L0d1b2b2a	Namibia
KC346212	L0d1c1a1b	Namibia
KC346213	L0d1b2b2b1	Namibia
KC346214	L0d1b2b2a	Namibia
KC346215	L0d1b2a1	Namibia
KC346216	L0d1b2b2a	Namibia
KC346217	L0d2c2a	Namibia
KC346218	L0d2c2b	Namibia
KC346219	L0d2c2b	Namibia
KC346220	L0d2a1a	Namibia
KC346221	L0d2a1a3	Namibia

KC346222	L0d2c1a	Namibia
KC346223	L0d2d	Namibia
KC346224	L0d3b2	Namibia
KC346225	L0d1a1b1b	Namibia
KC346226	L0d1a1a	Namibia
KC346227	L0d1a1a3	Namibia
KC346228	L0d1a1a3	Namibia
KC346229	L0d3b1	Namibia
KC346230	L0k1b	Namibia
KC346231	L0d1c1a1a2	Namibia
KC346232	L0d1c1a1a	Namibia
KC346233	L0k2a	Zambia
KC346234	L0d1b1c	Zambia
KC346235	L0d1b1b1	Zambia
KC346236	L0d1b1c	Zambia
KC346237	L0d1c2	Zambia
KC346238	L0d1c2	Zambia
KC346239	L0d1c2	Zambia
KC346240	L0d2a2	Zambia
KC346241	L0k1b	Zambia
KC346242	L0k2b	Zambia
KC346243	L0d1b1c	Zambia
KC346244	L0d1b1b1	Zambia
KC346245	L0d1b2a2	Zambia
KC346246	L0d1c2	Zambia
KC346247	L0d1b2b2b	Zambia
KC346248	L0d1b1b1	Zambia
KC533452	L3e1a1a	South Africa
KC533506	L3e1a1a	South Africa
KC533508	L2a1b1a	South Africa
KC533509	L0a2a2a	South Africa
KC533510	L0d2a1	South Africa
KC533512	L3e1b2	South Africa
KC533513	L2b2a	South Africa
KC533514	L1c1	South Africa
KC533516	L0d2a1a	South Africa
KC533518	L3e1b2	South Africa
KC622055	L3e3b1	Botswana
KC622056	L0a2a2a	Botswana
KC622057	L2a1b1a	Botswana
KC622058	L3f2a1	Botswana
KC622059	L3e1b2	Botswana
KC622060	L1c2b1b1	Botswana
KC622061	L3e1	Botswana
KC622062	L2a1b1a	Botswana
KC622063	L0a1b1a1	Botswana
KC622064	L0a2a2a	Botswana
KC622065	L0a2a1a2	Botswana
KC622066	L2a1b1a	Botswana
KC622067	L0a1b1a1	Botswana
KC622068	L0a1b1a1	Botswana
KC622069	L2a1a2	Botswana
KC622070	L2a1a2	Botswana
KC622071	L2a1b1a	Botswana
KC622072	L2a1a2	Botswana
KC622073	L3f1b1a	Botswana
KC622074	L2a1d2	Botswana
KC622075	L2c2b1b	Botswana
KC622076	L2a1f	Botswana
KC622077	L0a1b1a1	Botswana
KC622078	L0a1b1a1	Botswana
KC622079	L3e1a2	Namibia
KC622080	L3d3a1a	Namibia
KC622081	L3d3a1a	Namibia
KC622082	L3f1b4a	Namibia
KC622083	L3f1b4a	Namibia
KC622122	L3d3a1a	Namibia
KC622123	L3f1b4a	Namibia
KC622124	L3d3a1a	Namibia
KC622125	L1c1b	Namibia
KC622126	L3d3a1	Namibia
KC622127	L2b1a3	Namibia
KC622128	L3e1a2	Namibia
KC622129	L3d3a1	Namibia
KC622130	L3e1a2	Namibia
KC622131	L1b1a	Namibia
KC622132	L3e3b1	Namibia
KC622133	L1c3a1a	Namibia
KC622134	L3d3a1a	Namibia
KC622135	L3e1a2	Namibia
KC622136	L3d3a1a	Namibia
KC622137	L3d3a1a	Namibia
KC622138	L3f1b4a	Namibia
KC622139	L3f1b4a	Namibia
KC622140	L3d3a1a	Namibia
KC622141	L3d3a1a	Namibia
KC622142	L3e1a2	Namibia
KC622143	L3e1a2	Namibia
KC622144	L3f1b4a	Namibia
KC622145	L3e1a2	Namibia
KC622146	L3d3a1a	Namibia
KC622147	L3d3a1a	Namibia

KC622148	L3d3a1a	Namibia
KC622149	L3d3a1a	Namibia
KC622150	L0g	Namibia
KC622151	L0g	Namibia
KC622152	L3f1b1a1	Namibia
KC622153	L3d3a1a	Namibia
KC622154	L0a2a1a	Namibia
KC622155	L1c2a3	Namibia
KC622156	L3e1a2	Namibia
KC622157	L3d3a1a	Namibia
KC622158	L3f1b4a	Namibia
KC622159	L2a1q	Namibia
KC622198	L3d3a1a	Namibia
KC622199	L3d3a1a	Namibia
KC622200	L3d3a1a	Namibia
KC622201	L3d3a1a	Namibia
KC622202	L3e2b	Namibia
KC622203	L3d3a1a	Namibia
KC622204	L3d3a1a	Namibia
KC622205	L3e1a2	Namibia
KC622206	L3d3a1a	Namibia
KC622207	L3d3a1a	Namibia
KC622208	L3f1b4a	Namibia
KC622209	L3d3a1a	Namibia
KC622210	L3f1b4a	Namibia
KC622211	L3d3a1a	Namibia
KC622212	L3h1b2	Namibia
KC622213	L3d3a1a	Namibia
KC622214	L3d3a1a	Namibia
KC622215	L4b2a2c	Botswana
KC622216	L3e2b	Botswana
KC622217	L3e2b	Botswana
KC622218	L4b2a2c	Botswana
KC622219	L3d3a1	Botswana
KC622220	L2a1f	Botswana
KC622221	L2b1a3	Botswana
KC622222	L0a1b1a1	Botswana
KC622223	L1b1a10b	Botswana
KC622224	L3e1a2	Botswana
KC622225	L3d3a1b	Botswana
KC622226	L4b2a2c	Botswana
KC622227	L3e1e1	Botswana
KC622228	L3e2b3	Botswana
KC622229	L3d1a1a1	Botswana
KC622230	L3f1b4c	Botswana
KC622231	L1c2b1b1	Botswana

KC622232	L3e1e1	Botswana
KC622233	L1c2b1b1	Botswana
KC622234	L3e1e1	Botswana
KC622235	L3e1e1	Botswana
KC911364	L5b1a	Iran
KC911395	L2a1f3	Iran
KC911506	L3e3a	Iran
KC911529	L3d1a1a	Iran
KC911533	L3d1a1a	Iran
KF011502	L3f1b	Spain
KF011503	L3f1b	Spain
KF055291	L1b1a3	USA
KF055293	L3e2b8	USA
KF055296	L3d1a1a	USA
KF055297	L3d1b3	USA
KF055298	L1c3a	USA
KF055299	L2a1i1	USA
KF055300	L3e3b	USA
KF055302	L0a1a+200	USA
KF055303	L1b1a10	USA
KF055304	L3e2b+152	USA
KF055305	L1b1a	USA
KF055306	L3b1a	USA
KF055307	L3e2b+152	USA
KF055308	L3e1e	USA
KF055309	L3d1a1a1	USA
KF055310	L2c	USA
KF055311	L2c2a	USA
KF055313	L1b1a	USA
KF055314	L1b1	USA
KF055315	L3e1e	USA
KF055317	L1c2b2	USA
KF055318	L2a1a1	USA
KF055319	L2c	USA
KF055320	L3e2b1a1	USA
KF055321	L3e4a	USA
KF055322	L1b1a18	USA
KF055323	L2a1c+16129	USA
KF055324	L3b1a4	USA
KF055325	L2c1	USA
KF055326	L2a1a2	USA
KF055327	L2a1e1	USA
KF450918	L1c2b2	Pakistan
KF450919	L0a2a2a	Pakistan
KF450936	L0d3	Pakistan
KF451012	L1c4b	Central African Republic

KF451013	L0a2b	Congo
KF451014	L2a2b1a	Congo
KF451015	L1c1a1a1a	Central African Republic
KF451016	L1c1a2a2	Central African Republic
KF451017	L0a2a1	Central African Republic
KF451018	L1c1a1a1a	Central African Republic
KF451019	L1c1a1a1a	Central African Republic
KF451020	L0a2b	Congo
KF451021	L0a2a1	Central African Republic
KF451022	L1c1a2a1	Central African Republic
KF451023	L1c1a1a1b1	Central African Republic
KF451024	L1c1a1a1b	Central African Republic
KF451025	L1c4b	Central African Republic
KF451026	L5a1c	Congo
KF451027	L2a2b1a	Congo
KF451028	L1c1a1a1a	Central African Republic
KF451029	L0a2a1	Central African Republic
KF451030	L1c1a2b	Central African Republic
KF451031	L0a2b	Congo
KF451032	L2a2b1a	Congo
KF451033	L1c1a1a1b1	Central African Republic
KF451034	L1c4b	Central African Republic
KF451035	L2a4a	Congo
KF451036	L1c4b	Central African Republic
KF451037	L1c1a2b	Central African Republic
KF451038	L2a2a1	Congo
KF451039	L1c4b	Central African Republic
KF451040	L0a2b1	Congo
KF451041	L1c1a2a2	Central African Republic
KF451042	L2a4a	Congo
KF451043	L1c1a1a1a	Central African Republic
KF451096	L2a1+143+16189	Israel
KF451132	L2a1+143+16189	Israel
KF451138	L2b1a2	Israel
KF451441	L1b1a	Nigeria
KF451442	L2a1f	Nigeria
KF451443	L2a1a2b	Nigeria
KF451444	L3e1	Nigeria
KF451445	L2a1f	Nigeria
KF451446	L3d6	Nigeria
KF451447	L3e2a2	Nigeria
KF451448	L3f1b4b	Nigeria
KF451449	L1b1a18	Nigeria
KF451450	L2a1a2b	Nigeria
KF451451	L3e1e	Nigeria
KF451452	L2a1f2	Nigeria
KF451453	L2a1c5	Nigeria
KF451454	L1c3a1b	Nigeria
KF451455	L1b1a10	Nigeria
KF451456	L2a1a3c	Nigeria
KF451457	L2a1i1	Nigeria
KF451458	L3e3b1	Nigeria
KF451495	L1c4b	Central African Republic
KF451496	L0a2b	Congo
KF451497	L2a2b2	Congo
KF451498	L0a2b	Congo
KF451499	L1c1a2b	Central African Republic
KF451500	L1c4b	Central African Republic
KF451501	L0d1b2a1	Namibia
KF451502	L4b2a2c	Namibia
KF451503	L0d1c3	Namibia
KF451504	L0k1a1a	Namibia
KF451505	L2a1b1a	South Africa
KF451506	L0a2a2a	South Africa
KF451537	L3d3a1a	South Africa
KF451538	L0d1b2b2c1	Namibia
KF451539	L0d2a1a	South Africa
KF451540	L3d3a1a	South Africa
KF451541	L0d1b2a1	Namibia
KF451542	L0d1a1a1	South Africa
KF451543	L0d1b2b2b1	South Africa
KF451544	L3d3a1a	South Africa
KF672796	L0a	Cameroon
KF672797	L0k2a1	Mozambique
KF672798	L0k2a	Mozambique
KF672799	L0d1a1a	Mozambique
KF672800	L0b	Kenya
KF672801	L0d2c1	Mozambique
KF672802	L0d2b2	Mozambique
KF672803	L0d1c	Mozambique
KF672804	L0d1c	Mozambique

KF672805	L0a1b1a1	Mozambique
KF672806	L0a1b1a1a	Mozambique
KF672807	L0a1b1a	Chad
KF672808	L0a1e	Mozambique
KF672809	L0f2a1	Somalia
KF672810	L0a2	Somalia
KF672811	L0a1	Chad
KF672812	L0a1d	Ethiopia
KF672813	L0a2c	Somalia
KF672814	L0f2b	Sudan
KF672815	L0a1d	Kenya
KF672816	L0d1b2b2a	Mozambique
KF672817	L0f	Somalia
KF672818	L0d2c1	Mozambique
KF672819	L0a2a2a	Mozambique
KF672820	L0a1d	Somalia
KF672821	L0a1a+200	Ethiopia
KF672822	L0a1a2	São Tomé e Principe
KF672823	L0d3	Somalia
KF672824	L0a2a2a	São Tomé e Principe
KF672825	L0a2a2a	Somalia
KF672826	L0a1a2	São Tomé e Principe
KF672827	L0a1b1a	Cameroon
KF672828	L0f2b	Sudan
KF672829	L0a1a	Chad
KF672830	L0a1a+200	Ethiopia
KF672831	L0a2a1a	Niger
KF672832	L0a2a2a	Mozambique
KF672833	L0f2a	Cameroon
KJ185427	L2a5	Zambia
KJ185428	L2a1d2	Zambia
KJ185429	L2a1f	Zambia
KJ185430	L0a1a2	Zambia
KJ185431	L0a1b1a1	Zambia
KJ185432	L0a1+16293	Zambia
KJ185433	L0a2a1a	Zambia
KJ185434	L1b1a	Zambia
KJ185435	L1c2b1b1	Zambia
KJ185436	L1c2b2	Zambia
KJ185437	L1c1a2	Zambia
KJ185438	L1c2b1b1	Zambia
KJ185439	L1c2b1b1	Zambia
KJ185440	L2a1a2	Zambia
KJ185441	L2a5	Zambia
KJ185442	L2a1b1a	Zambia
KJ185443	L2b1a3	Zambia
KJ185444	L2b1b	Zambia
KJ185445	L3e2b	Zambia
KJ185446	L3e1	Zambia
KJ185447	L3e4a	Zambia
KJ185448	L3e1a1a	Zambia
KJ185449	L3e1a3a	Zambia
KJ185450	L1c2a1a	Zambia
KJ185451	L1c3a	Zambia
KJ185452	L2a1b1a	Zambia
KJ185453	L1c3a1b	Zambia
KJ185454	L1c2a1a	Zambia
KJ185455	L2a1a2a1a	Zambia
KJ185456	L3e3b1	Zambia
KJ185457	L0f1	Zambia
KJ185458	L0a2a2a2	Zambia
KJ185459	L2a1d2	Zambia
KJ185460	L2b1a3	Zambia
KJ185461	L0a2a1b	Zambia
KJ185462	L0a2a1b	Zambia
KJ185463	L0a2d	Zambia
KJ185464	L1c2b2	Zambia
KJ185503	L0a2a1b	Angola
KJ185504	L0a1b1a	Angola
KJ185505	L0a2a1b	Angola
KJ185506	L0a2a1b	Angola
KJ185507	L0a1b2	Angola
KJ185508	L0a2a1b	Angola
KJ185509	L0a1b1a	Angola
KJ185510	L0a1b1	Angola
KJ185511	L1b1a+189	Angola
KJ185512	L1c2a1b	Angola
KJ185513	L1c1b	Angola
KJ185514	L1c1b	Angola
KJ185515	L1c1b	Angola
KJ185516	L1c2a1a	Angola
KJ185517	L1c1b	Angola
KJ185518	L1c1b	Angola
KJ185519	L1c2b1b	Angola
KJ185520	L1c1b	Angola
KJ185521	L1c3b1a	Angola
KJ185522	L1c3b1a	Angola
KJ185523	L1c3b1a	Angola
KJ185524	L1c2b1b	Angola
KJ185525	L2a5	Angola
KJ185526	L2c2b1b	Angola

KJ185527	L3e2b+152	Angola	KJ185611	L3d3a1	Zambia
KJ185528	L3e4a	Angola	KJ185612	L3d3a1b	Zambia
KJ185529	L3e4a	Angola	KJ185613	L3d3a1	Zambia
KJ185530	L3e1e1	Angola	KJ185614	L3d4	Zambia
KJ185531	L3e1d1	Angola	KJ185615	L3d3a1	Zambia
KJ185532	L3f1b4a	Angola	KJ185616	L3e2b1a2	Zambia
KJ185533	L3f1b4a	Angola	KJ185655	L0a2a2a1	Zambia
KJ185534	L3f1b4a	Angola	KJ185656	L0a1a2	Zambia
KJ185535	L3h1b2	Angola	KJ185657	L0a2a2a	Zambia
KJ185536	L3d3a1b	Zambia	KJ185658	L0a2a2a	Zambia
KJ185537	L3e1a2	Zambia	KJ185659	L0a1b1a1	Zambia
KJ185538	L3e1a3a	Zambia	KJ185660	L0a1b1a1	Zambia
KJ185539	L2c2a1	Zambia	KJ185661	L1b1a	Zambia
KJ185540	L0a1b1a1	Zambia	KJ185662	L1c2b1a'b	Zambia
KJ185579	L1c2b1b1	Zambia	KJ185663	L1c2b1b1	Zambia
KJ185580	L1c2b2	Zambia	KJ185664	L1c1b	Zambia
KJ185581	L1c2a1a	Zambia	KJ185665	L1c2b1b1	Zambia
KJ185582	L2a1f3	Zambia	KJ185666	L1c3a1b	Zambia
KJ185583	L2a1f3	Zambia	KJ185667	L1c2b2	Zambia
KJ185584	L2a1c5	Zambia	KJ185668	L1c2b1b1	Zambia
KJ185585	L2a1c5	Zambia	KJ185669	L1c2b1b1	Zambia
KJ185586	L2a1d2	Zambia	KJ185670	L1c2b1b1	Zambia
KJ185587	L2a1b1a	Zambia	KJ185671	L1c2b1b1	Zambia
KJ185588	L2a1b1a	Zambia	KJ185672	L1c2a1a	Zambia
KJ185589	L2a1a1	Zambia	KJ185673	L1c2b1b1	Zambia
KJ185590	L2a1d2	Zambia	KJ185674	L1c1a2	Zambia
KJ185591	L2a1d2	Zambia	KJ185675	L1c1	Zambia
KJ185592	L2a5	Zambia	KJ185676	L1c2b1b1	Zambia
KJ185593	L2a5	Zambia	KJ185677	L1c2b2	Zambia
KJ185594	L2a5	Zambia	KJ185678	L2a1f	Zambia
KJ185595	L2a1c1	Zambia	KJ185679	L2a1a2a1a	Zambia
KJ185596	L2a1f3	Zambia	KJ185680	L2a1f	Zambia
KJ185597	L2a1i1	Zambia	KJ185681	L2a1f	Zambia
KJ185598	L2a1i1	Zambia	KJ185682	L2a5	Zambia
KJ185599	L2b1a3	Zambia	KJ185683	L2a1f	Zambia
KJ185600	L2b1a3	Zambia	KJ185684	L2a5	Zambia
KJ185601	L2c2a1	Zambia	KJ185685	L2a1f1	Zambia
KJ185602	L2c2b1b	Zambia	KJ185686	L2a1d2	Zambia
KJ185603	L2c2a1	Zambia	KJ185687	L2a5	Zambia
KJ185604	L2c2a1	Zambia	KJ185688	L2a1a	Zambia
KJ185605	L2c2a1	Zambia	KJ185689	L2a1d2	Zambia
KJ185606	L2e1a	Zambia	KJ185690	L2a1i1	Zambia
KJ185607	L2e1a	Zambia	KJ185691	L2a1d2	Zambia
KJ185608	L2e1a	Zambia	KJ185692	L2a1a	Zambia
KJ185609	L3b1a11	Zambia	KJ185731	L2a1c1	Zambia
KJ185610	L3b1a11	Zambia	KJ185732	L2a1a2a1a	Zambia

KJ185733	L2a1a2a1a	Zambia
KJ185734	L2a5	Zambia
KJ185735	L2a1b1a	Zambia
KJ185736	L3b1a1a	Zambia
KJ185737	L3b1a1	Zambia
KJ185738	L3b1a11	Zambia
KJ185739	L3e1a3a	Zambia
KJ185740	L3e1d1	Zambia
KJ185741	L3e4a	Zambia
KJ185742	L3e1a3a	Zambia
KJ185743	L3e1a3a	Zambia
KJ185744	L3e4a	Zambia
KJ185745	L3e4a	Zambia
KJ185746	L3e4a	Zambia
KJ185747	L3e1f1a	Zambia
KJ185748	L5a2	Zambia
KJ185749	L0a2a2a	Angola
KJ185750	L0a1b2	Angola
KJ185751	L0a2a1a	Angola
KJ185752	L0a2a1a	Angola
KJ185753	L0a1b1a	Angola
KJ185754	L0a1a2	Angola
KJ185755	L0a2a1b	Angola
KJ185756	L0a1b1a	Angola
KJ185757	L0a1b1a	Angola
KJ185758	L0a1b2	Angola
KJ185759	L0a1b1a	Angola
KJ185760	L1b1a15	Angola
KJ185761	L1c2b1b	Angola
KJ185762	L1c1b	Angola
KJ185763	L1c2b1b1	Angola
KJ185764	L1c2a3	Angola
KJ185765	L1c2a2	Angola
KJ185766	L1c3b1a	Angola
KJ185767	L1c1a2	Angola
KJ185768	L2a1a	Angola
KJ185807	L1b1a10	Angola
KJ185808	L1b1a	Angola
KJ185809	L1b1a10	Angola
KJ185810	L1b1a10	Angola
KJ185811	L1b1a	Angola
KJ185812	L1b1a10	Angola
KJ185813	L1c4b	Angola
KJ185814	L1c2b1a'b	Angola
KJ185815	L1c3a1a	Angola
KJ185816	L1c2b1a	Angola

KJ185817	L1c2b1a	Angola
KJ185818	L1c2b2	Angola
KJ185819	L1c2a3	Angola
KJ185820	L1c3b1a	Angola
KJ185821	L1c3c	Angola
KJ185822	L1c1b	Angola
KJ185823	L2a5	Angola
KJ185824	L2a1a2a1a	Angola
KJ185825	L2a1b1a	Angola
KJ185826	L2a1a2a1a	Angola
KJ185827	L2a1a1	Angola
KJ185828	L2a1b	Angola
KJ185829	L2a1a2	Angola
KJ185830	L2a5	Angola
KJ185831	L2b2a	Angola
KJ185832	L2b1a3	Angola
KJ185833	L2b2a	Angola
KJ185834	L2c2b1b	Angola
KJ185835	L3d3a1a	Angola
KJ185836	L3d1a2	Angola
KJ185837	L3d1b3a	Angola
KJ185838	L3d1a1a	Angola
KJ185839	L3e1	Angola
KJ185840	L3e1e1	Angola
KJ185841	L3e1a3	Angola
KJ185842	L3e1	Angola
KJ185843	L3e2b3	Angola
KJ185844	L3e2b3	Angola
KJ185883	L0a2a1a	Zambia
KJ185884	L0a1a2	Zambia
KJ185885	L1b1a3	Zambia
KJ185886	L1b1a10b	Zambia
KJ185887	L1c3b1a	Zambia
KJ185888	L1c2b1b1	Zambia
KJ185889	L1c2b1b1	Zambia
KJ185890	L1c2a1a	Zambia
KJ185891	L1c2b1b1	Zambia
KJ185892	L1c2b1b1	Zambia
KJ185893	L2a1f	Zambia
KJ185894	L2a1g	Zambia
KJ185895	L2a1f3	Zambia
KJ185896	L2a1a2	Zambia
KJ185897	L2a1f	Zambia
KJ185898	L2a1d2	Zambia
KJ185899	L2a1f	Zambia
KJ185900	L2a1i1	Zambia

KJ185901	L2a1d2	Zambia
KJ185902	L2e1a	Zambia
KJ185903	L3d3a1	Zambia
KJ185904	L3e1a3a	Zambia
KJ185905	L3e1e1	Zambia
KJ185906	L3e1d1a	Zambia
KJ185907	L3e1a3a	Zambia
KJ185908	L3e1a3a	Zambia
KJ185909	L3e1a3a	Zambia
KJ185910	L3e2b	Zambia
KJ185911	L3e3a	Zambia
KJ185912	L3e1a3a	Zambia
KJ185913	L3e1a3a	Zambia
KJ185914	L3e1a3a	Zambia
KJ185915	L3f2a1	Zambia
KJ185916	L1c2b1b1	Zambia
KJ185917	L1c2b1b1	Zambia
KJ185918	L1c3b1a	Zambia
KJ185919	L1c2b2	Zambia
KJ185920	L1c2b1b1	Zambia
KJ185959	L3e1e1	Zambia
KJ185960	L3e1e1	Zambia
KJ185961	L3e1e1	Zambia
KJ185962	L3e1a3a	Zambia
KJ185963	L1c2b1b1	Zambia
KJ185964	L1c3a	Zambia
KJ185965	L3d3a1	Zambia
KJ185966	L0a1b1a1	Zambia
KJ185967	L0a1b1a1	Zambia
KJ185968	L0a2a1a2	Zambia
KJ185969	L1c2a1a	Zambia
KJ185970	L0a2a2a	Zambia
KJ185971	L3e1f1a	Zambia
KJ185972	L0a1a2	Zambia
KJ185973	L2a1a2a1a	Zambia
KJ185974	L2b2	Zambia
KJ185975	L3d1a1a	Zambia
KJ185976	L3e1e1	Zambia
KJ185977	L0a1b1a1	Zambia
KJ185978	L0a1b1a1	Zambia
KJ185979	L0a1b1a1	Zambia
KJ185980	L1c2b1b1	Zambia
KJ185981	L1c3b1a	Zambia
KJ185982	L2a1a1	Zambia
KJ185983	L2c2a1	Zambia
KJ185984	L3e1a3a	Zambia
KJ185985	L2a1f	Zambia
KJ185986	L2a2b	Zambia
KJ185987	L2a1f1	Zambia
KJ185988	L3e2b	Zambia
KJ185989	L3f1b4a	Zambia
KJ185990	L0a1b1a1	Zambia
KJ185991	L3d3a1	Zambia
KJ185992	L3d3a1a	Zambia
KJ185993	L3d1a1a	Zambia
KJ185994	L2c2a1	Zambia
KJ185995	L0g	Zambia
KJ185996	L1c2a1a	Zambia
KJ445763	L0a2a2a	South Africa
KJ445764	L5a1c	Congo
KJ445765	L5a1	Kenya
KJ445766	L1c3a1b	Nigeria
KJ445767	L1c1a2b	Central African Republic
KJ445768	L1c1a2b	Central African Republic
KJ445769	L1c1a2b	Central African Republic
KJ445770	L1c1a2b	Central African Republic
KJ445771	L1c1a2b	Central African Republic
KJ445772	L1c1a2b	Central African Republic
KJ445773	L1c1a2b	Central African Republic
KJ445774	L1c1a2a1	Central African Republic
KJ445775	L1c1a2a2	Central African Republic
KJ445776	L1c1a2a2	Central African Republic
KJ445777	L1c1a2a2	Central African Republic
KJ445778	L1c1a1a1a	Central African Republic
KJ445779	L1c1a1a1a	Central African Republic
KJ445780	L1c1a1a1a	Central African Republic
KJ445781	L1c1a1a1a	Central African Republic
KJ445782	L1c1a1a1a	Central African Republic
KJ445783	L1c1a1a1a	Central African Republic
KJ445784	L1c1a1a1a	Central African Republic
KJ445785	L1c1a1a1a	Central African Republic
KJ445786	L1c1a1a1b1	Central African Republic

KJ445787	L1c1a1a1b1	Central African Republic
KJ445788	L1c1a1a1	Central African Republic
KJ445789	L1c4b	Central African Republic
KJ445790	L1c4	Central African Republic
KJ445791	L1c4b	Central African Republic
KJ445792	L1c4b	Central African Republic
KJ445793	L1c4b	Central African Republic
KJ445794	L1c4b	Central African Republic
KJ445795	L1c4b	Central African Republic
KJ445796	L1c4b	Central African Republic
KJ445797	L1c4b	Central African Republic
KJ445798	L1c2a1a	Kenya
KJ445799	L1c2a1a	Kenya
KJ445800	L1c2b2	Pakistan
KJ446543	L3e3b1	Nigeria
KJ446544	L3e1	Nigeria
KJ446545	L3e1e	Nigeria
KJ446546	L3e1b1	Israel
KJ446547	L3e1b1	Israel
KJ446548	L3e2b1	Algeria
KJ446549	L3e2b+152	Kenya
KJ446550	L3e2a1b1	Algeria
KJ446551	L3e2a2	Nigeria
KJ446552	L3e2a	Algeria
KJ446740	L2b1a2	Israel
KJ446741	L2b1a2	Israel
KJ446742	L2c1a	Senegal
KJ446743	L2c1a	Senegal
KJ446744	L2c5	Senegal
KJ446745	L2c3a	Senegal
KJ446746	L2c3a	Senegal
KJ446747	L2c3a	Senegal
KJ446748	L2a2a1	Congo
KJ446749	L2a2b2	Congo
KJ446750	L2a2b1a	Congo
KJ446751	L2a2b1a	Congo
KJ446752	L2a2b1a	Congo
KJ446753	L2a4a	Congo
KJ446754	L2a4a	Congo
KJ446755	L2a1c1	Senegal
KJ446756	L2a1c3b1	Senegal
KJ446757	L2a1c3b1	Senegal
KJ446758	L2a1c3b1	Senegal
KJ446759	L2a1c2	Senegal
KJ446760	L2a1f2	Nigeria
KJ446761	L2a1a3c	Nigeria
KJ446762	L2a1a2	Pakistan
KJ446763	L2a1a2b	Nigeria
KJ446764	L2a1i1	Nigeria
KJ446765	L2a1g	Pakistan
KJ446766	L2a1c5	Nigeria
KJ446767	L2a1c5	Nigeria
KJ669130	L0d1c1a1b	Namibia
KJ669131	L0d1c1a1a	Namibia
KJ669132	L0d1c1a1a	Namibia
KJ669133	L0d1c1a1b	Namibia
KJ669134	L0d1c1a2	Namibia
KJ669135	L0d1b1b	South Africa
KJ669136	L0d1c	Namibia
KJ669137	L0d1d	South Africa
KJ669138	L0d1a1d	South Africa
KJ669139	L0d1a1c	Namibia
KJ669140	L0d1a1a1	Namibia
KJ669141	L0d1a1b1b	South Africa
KJ669142	L0d1a1a	Namibia
KJ669143	L0d1a1a2	Namibia
KJ669144	L0d1b2b2b1	South Africa
KJ669145	L0d1b2b2a	South Africa
KJ669146	L0d1b2b2a	Namibia
KJ669147	L0d1b2b2c2	Namibia
KJ669148	L0d1b2b2c2	Namibia
KJ669149	L0d1b2a1	Namibia
KJ669150	L0d1b2b1b1	South Africa
KJ669151	L0d1b2b2c2	Namibia
KJ669152	L0d1b2a1	Namibia
KJ669153	L0d1b2b1b1	Namibia
KJ669154	L0d1b2b2b1	Namibia
KJ669155	L0d1b2b1b1	Namibia
KJ669156	L0d2c1	South Africa
KJ669157	L0d2c1	South Africa
KJ669158	L0d2c1	South Africa
KJ669159	L0d2c1a	Namibia
KJ669160	L0d2c1	Namibia
KJ669161	L0d2c2	Namibia
KJ669162	L0d2c2b	South Africa
KJ669163	L0d2c1a1	Namibia
KJ669164	L0d2c2a1	Namibia

KJ669165	L0d2c2	Namibia
KJ669166	L0d2b1b	South Africa
KJ669167	L0d2d	Namibia
KM101583	L3f1b1a	USA
KM101585	L3f1b1a	USA
KM101586	L3b2b	USA
KM101587	L3f1b1a	USA
KM101588	L1c3a	USA
KM101589	L3f1b+16292+150	USA
KM101590	L2a1b1	USA
KM101591	L3e2b+152	USA
KM101592	L2c	USA
KM101593	L2c	USA
KM101594	L2a1m1a	USA
KM101595	L1b1a	USA
KM101596	L3e2a1b1	USA
KM101597	L3b3	USA
KM101598	L0a1a2	USA
KM101599	L2c2	USA
KM101600	L3b2	USA
KM101601	L2a1f	USA
KM101602	L3e2b	USA
KM101603	L2a1f	USA
KM101604	L2a1c3a1	USA
KM101606	L3e2b	USA
KM101608	L3e3b	USA
KM101609	L2a1f	USA
KM101610	L2a1b1	USA
KM101611	L2a1f2	USA
KM101612	L3e2a1b1	USA
KM101613	L3e2a1b3	USA
KM101614	L3f1b+16292+150	USA
KM101615	L2b2	USA
KM101616	L1b1a7a	USA
KM101617	L3e4a	USA
KM101618	L2a1c5	USA
KM101619	L2c2	USA
KM101620	L1b1a6	USA
KM101621	L1b1a7	USA
KM101622	L3e2a1b1	USA
KM101625	L1b1a3a	USA
KM101666	L1b1a10	USA
KM101667	L2c4	USA
KM101668	L1c1b	USA
KM101669	L3h1b1a	USA
KM101670	L1b1a17	USA
KM101671	L3b1a	USA
KM101672	L3f1b1a	USA
KM101673	L2a1c	USA
KM101674	L2a1a1	USA
KM101675	L3f1b+16292+150	USA
KM101677	L3e3b	USA
KM101678	L0a1a2	USA
KM101679	L3f1b1a1	USA
KM101680	L1c3a	USA
KM101681	L3b3	USA
KM101682	L3b1b	USA
KM101683	L2a1f1	USA
KM101684	L2a1+143+@16309	USA
KM101685	L2a1l2	USA
KM101686	L3d1a2	USA
KM101687	L1b1a7	USA
KM101688	L3b1a1a	USA
KM101689	L2a1a1	USA
KM101692	L3f1b4a1	USA
KM101693	L2a1b1	USA
KM101694	L1c3b1b	USA
KM101695	L1b1a7	USA
KM101696	L2a1f	USA
KM101697	L1c3a1a	USA
KM101698	L3e2a1b	USA
KM101699	L1b1a14	USA
KM101700	L3f1b1a	USA
KM101701	L3f1b4c	USA
KM101702	L3f1b1a	USA
KM101703	L2a1e	USA
KM101704	L2a1c3b	USA
KM101705	L2c2a	USA
KM101706	L2a1a2	USA
KM102097	L1b1a7a	USA
KM102101	L3b1a10	USA
KM102103	L3e1e2	USA
KM102104	L2c	USA
KM102105	L3b1a+152	USA
KM102112	L3e1e2	USA
KM102120	L3d1'2'3'4'5'6	USA
KM102147	L3f1b4	USA
KM245150	L2a1+143+@16309	Saudi Arabia
KM245151	L3i1a	Saudi Arabia
KM245152	L3i1a	Ethiopia
KM986515	L2d1a	Yemen
KM986519	L0a2c	Yemen

KM986521	L2a1a2	Yemen
KM986523	L4b2a2c	Yemen
KM986524	L5a1b	Yemen
KM986525	L5a1a	Yemen
KM986528	L0f	Yemen
KM986532	L0a1a+200	Yemen
KM986536	L2a1a2a1a	Yemen
KM986538	L3x1+16311	Yemen
KM986543	L3x1+16311	Yemen
KM986544	L3f	Yemen
KM986546	L3d3a1a	Yemen
KM986547	L3h1b1a	Yemen
KM986549	L3x2a	Yemen
KM986551	L3h1b1a	Yemen
KM986553	L3k1	Yemen
KM986555	L0a2c	Yemen
KM986556	L3x1+16311	Yemen
KM986559	L3x2a	Yemen
KM986561	L3e2b	Yemen
KM986563	L0a2a2a	Yemen
KM986564	L3h1a2b	Yemen
KM986566	L3x1	Yemen
KM986567	L3b1a1a	Yemen
KM986571	L0a2c	Yemen
KM986575	L3b1a1a	Yemen
KP229451	L3i1	Unknown
KP229452	L3i1	Unknown
KP229453	L3i1	Unknown
KP229454	L3i1	Unknown
KP229455	L3i1	Unknown
KP240909	L2a2b1a	Congo
KP240910	L2a1a2b	Nigeria
KP240911	L2a1b1a	South Africa
KP240912	L2a1+143+16189	Israel
KP240913	L2a1c+16129	Israel
KP240914	L3d1a1a	Pakistan
KP240915	L3d1b	Senegal
KP240928	L0d1a1a1	South Africa
KP240929	L0a2b	Congo
KP240930	L0a2b	Congo
KP317053	L4b2b	Niger
KP317054	L4a1a	United Arab Emirates
KP317055	L4b2a2	United Arab Emirates
KP317056	L4b2a2c	Ethiopia
KP317057	L6a	Ethiopia
KP317058	L4a1a	Ethiopia
KP317059	L4a1a	Ethiopia
KP317060	L6b	Ethiopia
KP317061	L4a1a	Ethiopia
KP317062	L4b1a	Burkina Faso
KP317063	L4b2b	Chad
KP317064	L4b2b	Chad
KP317065	L4b2b1	Niger
KP317066	L4b2a2a	Ethiopia
KP317067	L6b	Kenya
KP317068	L4b2a2b	Kenya
KP317069	L4b2a2a	Sudan
KP317070	L4b2a2	Sudan
KP317071	L6a	Somalia
KP317072	L4b2a2c	Somalia
KP317073	L4a2	Somalia
KP317074	L4b2a1	Somalia
KP317075	L4b2a	Somalia
KR135863	L2c2	São Tomé e Principe
KR135864	L2c	São Tomé e Principe
KR135865	L2b2a	São Tomé e Principe
KR135866	L2a1a3c	São Tomé e Principe
KR135867	L2a1+143	Ethiopia
KR135868	L2a1+143+16189	Ethiopia
KR135869	L2a1+143	Ethiopia
KR135870	L2a1c+16129	Ethiopia
KR135871	L2a1a	Mozambique
KR135872	L2a1a2	Mozambique
KR135873	L2a1b1a	Mozambique
KR135874	L2a1b1a	Mozambique
KR135875	L2a1b1a	Mozambique
KR135876	L2a1a2	Mozambique
KR135877	L2a1b1a	Mozambique
KR135878	L2a1a2	Mozambique
KR135879	L2a1a2	Mozambique
KR135880	L2a1h	Mozambique
KR135881	L2a1a2a1a	Mozambique
KR135882	L2a1b1a	Mozambique
KR135883	L2a1a	Mozambique
KR135884	L2a1d2	Mozambique
KT756878	L2d+16129	Mexico
KT819205	L1b1a12a	Spain
KT819206	L1b1a6	Spain
KT819207	L2a1+143+16189	Spain

KT819208	L2a1c6	Spain
KT819209	L2b1a	Spain
KT819210	L2b3a	Spain
KT819211	L3f1b1	Spain
KT819224	L2a1b+143	Spain
KT819225	L3d1b1	Spain
KT819226	L3d3b	Spain
KT819227	L3h1b1a	Spain
KT819228	L3x2b	Spain
KT819237	L1b1a6	Morocco
KT819238	L2a1+143	Morocco
KT819239	L2b1a	Morocco
MF055747	L3e3b1	Madagascar
MF055748	L3b1a1a	Madagascar
MF055749	L1c2a2	Madagascar
MF055750	L3e3b1	Madagascar
MF055752	L2a1b1a	Madagascar
MF055753	L3e3b1	Madagascar
MF055754	L3b1a1a	Madagascar
MF055756	L0d1c	Madagascar
MF055757	L3b1a1a	Madagascar
MF055758	L3d1a1a1	Madagascar
MF055759	L3d1a1a	Madagascar
MF055760	L2a1b1a	Madagascar
MF055761	L0a2	Madagascar
MF055762	L3b1a1a	Madagascar
MF055765	L3e3a	Madagascar
MF055775	L0a1b1a1	Madagascar
MF055780	L3b1a8	Madagascar
MF055781	L3e3b1	Madagascar
MF055783	L1c3c	Madagascar
MF055785	L2a1a2	Madagascar
MF055786	L0a1b1a1	Madagascar
MF055787	L0a2a2a	Madagascar
MF055789	L2a1a2	Madagascar
MF055790	L3b1a3	Madagascar
MF055791	L3e3b1	Madagascar
MF055795	L3a+709	Madagascar
MF055803	L3e3a	Madagascar
MF055804	L3d1a1a	Madagascar
MF055805	L3d1a1a	Madagascar
MF055807	L3d1a1a	Madagascar
MF055809	L3d1a1a1	Madagascar
MF055810	L3e1a1a	Madagascar
MF055811	L0a2a1a	Madagascar
MF055812	L3e3b1	Madagascar
MF055815	L0a1b1a1	Madagascar
MF055816	L2a1b1a	Madagascar
MF055818	L3b1a1a	Madagascar
MF055821	L3e1a3a	Madagascar
MF055918	L3e3a	Madagascar
MF055925	L0a2a2a	Madagascar
MF055928	L0a2a2a	Madagascar
MF055929	L3e3b1	Madagascar
MF055932	L0a2a2a	Madagascar
MF055933	L0a2a2a	Madagascar
MF055934	L1c3c	Madagascar
MF055936	L2a1b1a	Madagascar
MF055937	L2a1b1a	Madagascar
MF055939	L0a2a1b	Madagascar
MF055940	L2a1b1a	Madagascar
MF055943	L2a1b1a	Madagascar
MF055944	L1c1d	Madagascar
MF055945	L0f	Madagascar
MF055954	L0a1b1a1	Madagascar
MF055957	L3b1a8	Madagascar
MF055959	L3b1a1a	Madagascar
MF055961	L0a2a2a	Madagascar
MF055962	L3e1a1a	Madagascar
MF055963	L3e3a	Madagascar
MF055966	L0a1b1a1	Madagascar
MF055969	L3k1	Madagascar
MF055970	L2a1b1a	Madagascar
MF055971	L3d1a1a	Madagascar
MF055973	L0a1b1a	Madagascar
MF055974	L3d1a1a	Madagascar
MF055978	L3d1a1a	Madagascar
MF055979	L3e1a3a	Madagascar
MF055980	L3e3a	Madagascar
MF055986	L0a2a2a	Madagascar
MF055988	L0a2a2a	Madagascar
MF055989	L3b1a1a	Madagascar
MF055991	L3d1a1a	Madagascar
MF055993	L0a1b1a1	Madagascar
MF056005	L1b2a	Madagascar
MF056008	L3e1a3a	Madagascar
MF056009	L3b1a1a	Madagascar
MF056010	L2a1b1a	Madagascar
MF056132	L2a1f	Madagascar
MF056134	L2a1f	Madagascar
MF056142	L3b1a1a	Madagascar
MF056145	L2a1b1a	Madagascar

MF056150	L3e2b+152	Madagascar
MF056151	L1b2a	Madagascar
MF056155	L3b1a1a	Madagascar
MF056157	L2a1b1a	Madagascar
MF056158	L2a1b1a	Madagascar
MF056159	L2a1b1a	Madagascar
MF056160	L2a1a2	Madagascar
MF056161	L3b1a1a	Madagascar
MF056163	L2a1b1a	Madagascar
MF056167	L3b1a1a	Madagascar
MF056169	L2a5	Madagascar
MF056172	L2a1a	Madagascar
MF056175	L2a1f3	Madagascar
MF056176	L2a1a3c	Madagascar
MF056177	L3d1a1a	Madagascar
MF056187	L3e1a3a	Madagascar
MF056190	L0a2a2a	Madagascar
MF056194	L3b1a1a	Madagascar
MF056195	L2a1b1a	Madagascar
MF056199	L3a+709	Madagascar
MF056204	L3e3b1	Madagascar
MF056210	L2a5	Madagascar
MF056212	L3a+709	Madagascar
MF056213	L2a1b1a	Madagascar
MF056214	L3b1a1a	Madagascar
MF056219	L0a2a1b	Madagascar
MF056221	L2a1a2	Madagascar
MF056223	L3a+709	Madagascar
MF056224	L2a1a2	Madagascar
MF056225	L3b1a1a	Madagascar
MF056235	L3a+709	Madagascar
MF056238	L3b1a1a	Madagascar
MF056239	L3b1a1a	Madagascar
MF056251	L3b1a1a	Madagascar
MF056342	L2a1b1a	Madagascar
MF056344	L2a1a2	Madagascar
MF056345	L3d1a	Madagascar
MF056348	L2a1b1a	Madagascar
MF056351	L3e3b2	Madagascar
MF056354	L0a2a2a	Madagascar
MF056355	L3d1a1a1	Madagascar
MF056359	L0a2a2a	Madagascar
MF056360	L2a1a	Madagascar
MF056361	L0f	Madagascar
MF056364	L1c2b2	Madagascar
MF056365	L2a1f3	Madagascar
MF056368	L3b1a1a	Madagascar
MF056371	L3a1b	Madagascar
MF056373	L3a1b	Madagascar
MF056376	L2b1a3	Madagascar
MF056381	L3d1a1a1	Madagascar
MF056383	L0a1b1a1	Madagascar
MF056384	L3d1a1a	Madagascar
MF056386	L3e1a3a	Madagascar
MF056387	L0a2a2a	Madagascar
MF056392	L3b2	Madagascar
MF056393	L3b1a1a	Madagascar
MF056394	L3e1a3a	Madagascar
MF056395	L1c2a1a	Madagascar
MF056397	L2a1b1a	Madagascar
MF056398	L3e3a	Madagascar
MF056402	L3e3b1	Madagascar
MF056403	L0a2a2a	Madagascar
MF056404	L3d1a1a	Madagascar
MF056407	L2a1f	Madagascar
MF056408	L3d3b	Madagascar
MF056409	L3d1a1a	Madagascar
MF056410	L2a1b1a	Madagascar
MF056412	L2a1a2	Madagascar
MF056415	L3b1a11	Madagascar
MF056416	L3e1b2	Madagascar
MF056418	L3e3a	Madagascar
MF056512	L3b1a1a	Madagascar
MF056514	L2a1a2	Madagascar
MF056516	L3d1a1a	Madagascar
MF056518	L3b1a1a	Madagascar
MF056526	L3d1a1a	Madagascar
MF056529	L3d1a1a	Madagascar
MF056533	L2a1b1a	Madagascar
MF056534	L3d1a1a	Madagascar
MF056536	L3d3b	Madagascar
MF056537	L0a2a2a	Madagascar
MF056538	L3e1a1a	Madagascar
MF056539	L3d1a1a	Madagascar
MF056542	L3e2b	Madagascar
MF056544	L2a1b1a	Madagascar
MF056545	L0a2a2a	Madagascar
MF056548	L2a1a2	Madagascar
MF056553	L3b1a11	Madagascar
MF056554	L2a1a2	Madagascar
MF056555	L2a1b1a	Madagascar
MF056556	L2a1a2	Madagascar

MF056558	L2a1a2	Madagascar
MF056559	L2a1b1a	Madagascar
MF056561	L2a1b1a	Madagascar
MF056562	L1c2a1a	Madagascar
MF056563	L3e3a	Madagascar
MF056564	L0f	Madagascar
MF056565	L1c3c	Madagascar
MF056566	L2a1b1a	Madagascar
MF056567	L1c2a1a	Madagascar
MF056568	L2a1b1a	Madagascar
MF056571	L2a1b1a	Madagascar
MF056573	L3b1a1a	Madagascar
MF056575	L4b2a	Madagascar
MF056582	L3b1a1a	Madagascar
MF056587	L3b1a8	Madagascar
MF056589	L3b1a1a	Madagascar
MF056591	L3d1a1a	Madagascar
MF056594	L2a1a2	Madagascar
MF056702	L3e1a3a	Madagascar
MF056703	L3b1a1a	Madagascar
MF056706	L3e1d1	Madagascar
MF056707	L2a1a2	Madagascar
MF056709	L3d1a1a	Madagascar
MF056711	L2a1a2	Madagascar
MF056712	L3f1b4a	Madagascar
MF056715	L2a1b1a	Madagascar
MF056721	L3b1a1a	Madagascar
MF056724	L0a2a2a	Madagascar
MF056728	L3e2b+152	Madagascar
MF056735	L3d1a1a	Madagascar
MF056741	L3e2b+152	Madagascar
MF056745	L3b1a1a	Madagascar
MF056747	L4b1a	Madagascar
MF056748	L3e1a1a	Madagascar
MF056749	L2a1b1a	Madagascar
MF056750	L2a1a2	Madagascar
MF056753	L3e1a1a	Madagascar
MF056755	L3e1a1a	Madagascar
MF056763	L3e1a1a	Madagascar
MF056764	L2a1b1a	Madagascar
MF056766	L3e3a	Madagascar
MF056768	L3b1a8	Madagascar
MF056776	L3e3a	Madagascar
MF056777	L3e1e	Madagascar
MF056780	L2b1a3	Madagascar
MF056785	L3b1a8	Madagascar
MF056794	L1c3a	Madagascar
MF056797	L3e1d1	Madagascar
MF056805	L3e1d1	Madagascar
MF056810	L3b1a8	Madagascar
MF056811	L2a1b1a	Madagascar
MF056814	L3d1a1a	Madagascar
MF056818	L3b1a8	Madagascar
MF056819	L3d1a1a	Madagascar
MF056820	L1c3b1a	Madagascar
MF056821	L3b1a1a	Madagascar
MF056936	L3d1a1a1	Madagascar
MF056940	L2a1a2	Madagascar
MF056945	L2a1b1a	Madagascar
MF056946	L2a1b1a	Madagascar
MF056948	L1c2a1a	Madagascar
MF056949	L3b1a11	Madagascar
MF056955	L3d1a1a1	Madagascar
MF056958	L3d1a1a	Madagascar
MF056959	L3e1a3a	Madagascar
MF056960	L3e1a3a	Madagascar
MF056963	L3e1d1	Madagascar
MF056965	L3e1a3a	Madagascar
MF056966	L3d1a1a	Madagascar
MF056967	L3d1a1a	Madagascar
MF056969	L3e1a3a	Madagascar
MF056971	L2a1b1a	Madagascar
MF056976	L2a1b1a	Madagascar
MF056979	L3e1a1a	Madagascar
MF056980	L2a1b1a	Madagascar
MF056986	L2a1b1a	Madagascar
MF056987	L3d1a1a1	Madagascar
MF056991	L3e2b+152	Madagascar
MF056993	L3e3b1	Madagascar
MF056994	L3e1a3a	Madagascar
MF056998	L2a1a2	Madagascar
MF057002	L3x1a2	Madagascar
MF057008	L3e1a1a	Madagascar
MF057011	L2b2a	Madagascar
MF057013	L2a1a2	Madagascar
MF057014	L0a2a2a	Madagascar
MF057019	L3b1a1a	Madagascar
MF057020	L3e3a	Madagascar
MF057025	L2a1a2	Madagascar
MF057026	L2a1b1a	Madagascar
MF057027	L3f1b4a1	Madagascar
MF057028	L2a1b1a	Madagascar

MF057029	L0a2a2a	Madagascar
MF057031	L3e3a	Madagascar
MF057133	L2a1b1a	Madagascar
MF057135	L2a1b1a	Madagascar
MF057137	L3a+709	Madagascar
MF057138	L1c2b1a'b	Madagascar
MF057141	L0a2a2a	Madagascar
MF057143	L2a1b1a	Madagascar
MF057147	L0a2a2a	Madagascar
MF057148	L0a2a2a	Madagascar
MF057150	L0a1b1a1	Madagascar
MF057151	L2a1a2	Madagascar
MF057153	L2a1f3	Madagascar
MF057154	L0d2a'b'd	Madagascar
MF057155	L0d1c	Madagascar
MF057157	L1c1d	Madagascar
MF057158	L2a1f3	Madagascar
MF057159	L3e3a	Madagascar
MF057160	L2a1f3	Madagascar
MF057161	L0a2a2a	Madagascar
MF057162	L0a1b1a1	Madagascar
MF057164	L3b1a1a	Madagascar
MF057165	L3b1a1a	Madagascar
MF057166	L2a1g	Madagascar
MF057168	L3e3a	Madagascar
MF057171	L1c3a	Madagascar
MF057172	L3e3a	Madagascar
MF057175	L3e1b2	Madagascar
MF057176	L3b1a1a	Madagascar
MF057177	L0a2a2a	Madagascar
MF057179	L3d1a1a	Madagascar
MF057180	L2a1b1a	Madagascar
MF057181	L3e3a	Madagascar
MF057182	L3e1b2	Madagascar
MF057183	L2a1b1a	Madagascar
MF057184	L2a1a2	Madagascar
MF057189	L3e3b2	Madagascar
MF057190	L2c2b1b	Madagascar
MF057193	L2a1a	Madagascar
MF057195	L2a1b1a	Madagascar
MF057277	L3a+709	Madagascar
MF057279	L2a1b1a	Madagascar
MF057282	L2a1b1a	Madagascar
MF057291	L3e3b1	Madagascar
MF057293	L3d1a1a1	Madagascar
MF057294	L3e3b1	Madagascar
MF057299	L3b1a1a	Madagascar
MF057300	L3b1a1a	Madagascar
MF057305	L0a2a2a	Madagascar
MF057308	L3d1a1a1	Madagascar
MF057313	L1c3a	Madagascar
MF057314	L2a1b1a	Madagascar
MF057315	L2a1b1a	Madagascar
MF057316	L2a1h	Madagascar
MF057318	L3b1a1a	Madagascar
MF057320	L3e1a3a	Madagascar
MF057325	L2a1b1a	Madagascar
MF057326	L3e3a	Madagascar
MF057327	L3e2b	Madagascar
MF057328	L3e3b2	Madagascar
MF057329	L3e3a	Madagascar
MF057332	L3b1a8	Madagascar
MF057333	L3d1a1a1	Madagascar
MF057334	L3d1a1a1	Madagascar
MF057335	L0a1+16293	Madagascar
MF057336	L2a1b1a	Madagascar
MF057338	L2a1a2	Madagascar
MF057339	L3e1a3a	Madagascar
MF057340	L2a1b1a	Madagascar
MF057342	L0a1b1a1	Madagascar
MF057345	L3e3a	Madagascar
MF057346	L0a	Madagascar
MF057347	L0a	Madagascar
MF057348	L3b1a1a	Madagascar
MF057351	L3d1a1a1	Madagascar
MF057352	L3b1a1a	Madagascar
MF057356	L2a1a2	Madagascar
MF057360	L1c1d	Madagascar
MF057446	L3e1a3a	Madagascar
MF057449	L1c1d	Madagascar
MF057450	L2a1b1a	Madagascar
MF057451	L0a2a2a	Madagascar
MF057455	L2a1b1a	Madagascar
MF057459	L0a2a2a	Madagascar
MF057460	L3e1b2	Madagascar
MF057461	L2a1b1a	Madagascar
MF057464	L3b1a1a	Madagascar
MF057465	L2a1b1a	Madagascar
MF057469	L2a1b1a	Madagascar
MF057470	L1c1d	Madagascar
MF057472	L2a1b1a	Madagascar
MF057473	L2a1a2	Madagascar

MF057474	L2b1a3	Madagascar	MF057686	L0a2a2a	Madagascar
MF057475	L2a1b1a	Madagascar	MF057692	L3b1a1a	Madagascar
MF057477	L2a1b1a	Madagascar	MF057693	L0a2a2a	Madagascar
MF057478	L2a1b1a	Madagascar	MF057694	L0a2a2a	Madagascar
MF057480	L2a1b1a	Madagascar	MF057696	L0a2a2a	Madagascar
MF057481	L3e3a	Madagascar	MF057698	L2a1a2	Madagascar
MF057482	L3b1a1a	Madagascar	MF057702	L3b1a1a	Madagascar
MF057487	L0a2a2a	Madagascar	MF057708	L3b1a1a	Madagascar
MF057498	L3b1a1a	Madagascar	MF057711	L2a1a2	Madagascar
MF057501	L0a2a2a	Madagascar	MF057712	L3e1a3a	Madagascar
MF057502	L0a1b1a1	Madagascar	MF057713	L3e1a3a	Madagascar
MF057503	L3e1a1a	Madagascar	MF057714	L3b1a1a	Madagascar
MF057504	L0a1b1a1	Madagascar	MF057715	L3e3a	Madagascar
MF057507	L3d3a1	Madagascar	MF057718	L3b1a1a	Madagascar
MF057508	L2a1a	Madagascar	MF057719	L3e1a3a	Madagascar
MF057509	L3b1a1a	Madagascar	MF057720	L2a1+143	Madagascar
MF057511	L0a1b1a1	Madagascar	MF057814	L3b1a1a	Madagascar
MF057512	L1c3c	Madagascar	MF057815	L2a1b1a	Madagascar
MF057515	L2a1a2	Madagascar	MF057818	L0a1b1a1	Madagascar
MF057517	L3e2b+152	Madagascar	MF057820	L0a2a2a	Madagascar
MF057518	L3e1d1	Madagascar	MF057821	L0a	Madagascar
MF057520	L3b1a1a	Madagascar	MF057822	L3e3a	Madagascar
MF057521	L1c1d	Madagascar	MF057824	L0a2a2a	Madagascar
MF057522	L3e2b	Madagascar	MF057828	L2a1b1a	Madagascar
MF057631	L3b1a1a	Madagascar	MF057829	L3e2b3	Madagascar
MF057633	L3b1a1a	Madagascar	MF057831	L3e1a3a	Madagascar
MF057635	L0a2a2a	Madagascar	MF057832	L1c3a1b	Madagascar
MF057636	L3e1e1	Madagascar	MF057833	L3b1a3	Madagascar
MF057637	L3e1e1	Madagascar	MF057834	L3d1a1a	Madagascar
MF057638	L2a1b1a	Madagascar	MF057838	L3e3a	Madagascar
MF057639	L2a1a2	Madagascar	MF057842	L3e3a	Madagascar
MF057644	L3d1a1a	Madagascar	MF057843	L1c3c	Madagascar
MF057645	L2a1f	Madagascar	MF057844	L3b1a1a	Madagascar
MF057646	L2a1b1a	Madagascar	MF057848	L3b1a8	Madagascar
MF057648	L2a1b1a	Madagascar	MF057849	L0a2a2a	Madagascar
MF057650	L0a2a2a	Madagascar	MF057850	L4b2a1	Madagascar
MF057651	L3b1a1a	Madagascar	MF057852	L3e1	Madagascar
MF057653	L3d1a1a	Madagascar	MF057853	L3d1a1a	Madagascar
MF057655	L3d1a1a	Madagascar	MF057854	L3e1a1a	Madagascar
MF057659	L3d1a1a	Madagascar	MF057856	L3b1a8	Madagascar
MF057664	L2a1b1a	Madagascar	MF057857	L3f1b4a1	Madagascar
MF057667	L0a1b1a1	Madagascar	MF057859	L3f1b4a1	Madagascar
MF057671	L3d1a1a1	Madagascar	MF057862	L3b1a8	Madagascar
MF057679	L3b1a1a	Madagascar	MF057863	L3e1d1	Madagascar
MF057682	L0a2a2a	Madagascar	MF057864	L0a2a2a	Madagascar
MF057683	L3e3a	Madagascar	MF057872	L2a1b1a	Madagascar

MF057874	L3e1a3a	Madagascar	MF058140	L3b1a1a	Madagascar
MF057877	L3e1a3a	Madagascar	MF058142	L2b1a3	Madagascar
MF057879	L2a1a3c	Madagascar	MF058144	L2a1b1a	Madagascar
MF057882	L3d1a1a1	Madagascar	MF058145	L3b1a1a	Madagascar
MF057884	L2b1a3	Madagascar	MF058146	L1c2a1a	Madagascar
MF057885	L3e1a3a	Madagascar	MF058147	L2a1b1a	Madagascar
MF057887	L0a2a2a	Madagascar	MF058149	L3d1a1a1	Madagascar
MF057889	L3e3a	Madagascar	MF058152	L3e1d1	Madagascar
MF057981	L3h1a2	Madagascar	MF058153	L2a1b1a	Madagascar
MF057985	L3d1a1a	Madagascar	MF058154	L1c3c	Madagascar
MF057986	L3b1a1a	Madagascar	MF058155	L2a1b1a	Madagascar
MF057987	L0a1b1a1	Madagascar	MF058157	L3k1	Madagascar
MF057994	L3b1a8	Madagascar	MF058158	L2a5	Madagascar
MF058012	L1c3c	Madagascar	MF058159	L2a1g	Madagascar
MF058013	L3e3b1	Madagascar	MF058161	L3e1d1	Madagascar
MF058018	L2a1b1a	Madagascar	MF058162	L2a1b1a	Madagascar
MF058021	L3a+709	Madagascar	MF058163	L0a1b1a1	Madagascar
MF058025	L3f	Madagascar	MF058165	L3e1a3a	Madagascar
MF058026	L3e3a	Madagascar	MF058166	L1c2a1a	Madagascar
MF058030	L3b1a1a	Madagascar	MF058167	L3e1a3a	Madagascar
MF058032	L3b1a1a	Madagascar	MF058168	L3e2b1a2	Madagascar
MF058038	L3e3b1	Madagascar	MF058169	L3e3b1	Madagascar
MF058039	L3e3b1	Madagascar	MF058170	L3e2b1a2	Madagascar
MF058040	L2a1b1a	Madagascar	MF058173	L3e3a	Madagascar
MF058043	L2a1a3c	Madagascar	MF058174	L3b1a1a	Madagascar
MF058044	L2a1b1a	Madagascar	MF058176	L3d1a1a	Madagascar
MF058048	L3b1a1a	Madagascar	MF058177	L1c2a1a	Madagascar
MF058049	L2a1b1a	Madagascar	MF058179	L4b2a1	Madagascar
MF058051	L3b1a1a	Madagascar	MF058182	L0a2	Madagascar
MF058052	L0a1b1a1	Madagascar	MF058183	L2a1a	Madagascar
MF058054	L2a1b1a	Madagascar	MF058184	L4b2a1	Madagascar
MF058055	L2a1b1a	Madagascar	MF058185	L4b2a1	Madagascar
MF058059	L3a+709	Madagascar	MF058187	L3d1a1a	Madagascar
MF058061	L3d1a1a	Madagascar	MF058188	L0a2	Madagascar
MF058066	L0a2a2a	Madagascar	MF058190	L3d1a1a1	Madagascar
MF058067	L0a2a2a	Madagascar	MF058191	L0a2a2a	Madagascar
MF058069	L1c2a3a	Madagascar	MF058193	L2b2a	Madagascar
MF058070	L3e3b1	Madagascar	MF058194	L3k1	Madagascar
MF058071	L3e1a3a	Madagascar	MF058276	L3b1a1a	Madagascar
MF058072	L2a1b1a	Madagascar	MF058281	L3b1a1a	Madagascar
MF058074	L3e3a	Madagascar	MF058287	L3b1a1a	Madagascar
MF058075	L3e1b2	Madagascar	MF058288	L3e1e1	Madagascar
MF058077	L1c3b1a	Madagascar	MF058289	L0a2a2a	Madagascar
MF058078	L2a1b1a	Madagascar	MF058292	L1c1	Madagascar
MF058079	L3e1a1a	Madagascar	MF058293	L3e3b1	Madagascar
MF058081	L3b1a8	Madagascar	MF058296	L2a1b1a	Madagascar

MF058308	L3b1a1a	Madagascar
MF058309	L3e1a3a	Madagascar
MF058310	L3b1a1a	Madagascar
MF058313	L3e3a	Madagascar
MF058318	L3d1a1a	Madagascar
MF058322	L0a1b1a1	Madagascar
MF058330	L0a1b1a1	Madagascar
MF058335	L0a2a1a	Madagascar
MF058339	L3b1a1a	Madagascar
MF058340	L2a1b1a	Madagascar
MF058341	L3d1a1a1	Madagascar
MF058342	L3b1a1a	Madagascar
MF058345	L2a1b1a	Madagascar
MF058346	L2a1b1a	Madagascar
MF058350	L3b1a1a	Madagascar
MF058352	L3b1a1a	Madagascar
MF058355	L3e3a	Madagascar
MF058356	L3b1a1a	Madagascar
MF058363	L1c3a	Madagascar
MF058365	L0a2a2a	Madagascar
MF058366	L3d1a1a	Madagascar
MF058367	L3d1a1a	Madagascar
MF058368	L3e1a3a	Madagascar
MF058371	L4b2a1	Madagascar
MF058373	L3e2b+152	Madagascar
MF058374	L2a1b1a	Madagascar
MF058375	L3b1a8	Madagascar
MF058382	L4b2a1	Madagascar
MF058383	L3b1a1a	Madagascar
MF058384	L2a1a2	Madagascar
MF058472	L3b1a1a	Madagascar
MF058479	L3b1a1a	Madagascar
MF058483	L3b1a1a	Madagascar
MF058486	L3b1a1a	Madagascar
MF058490	L3b1a1a	Madagascar
MF058498	L3e2b3	Madagascar
MF058505	L3e1a3a	Madagascar
MF058506	L3b1a1a	Madagascar
MF058507	L0a2a2a	Madagascar
MF058509	L2a1a2	Madagascar
MF058511	L2a1b1a	Madagascar
MF058515	L2a1a2	Madagascar
MF058521	L0a2a2a	Madagascar
MF058525	L1c3c	Madagascar
MF058528	L3e3b1	Madagascar
MF058529	L2a1f	Madagascar
MF058530	L0a2a2a	Madagascar
MF058531	L3e3a	Madagascar
MF058533	L0a2a2a	Madagascar
MF058535	L0f	Madagascar
MF058536	L1c3c	Madagascar
MF058537	L3b1a1a	Madagascar
MF058539	L0a2a2a	Madagascar
MF058540	L2a1b1a	Madagascar
MF058541	L2a1b1a	Madagascar
MF058544	L3e1e	Madagascar
MF058545	L3e3a	Madagascar
MF058546	L3e3a	Madagascar
MF058547	L3e3a	Madagascar
MF058549	L3d1a1a	Madagascar
MF058550	L3e3a	Madagascar
MF058552	L3e2b+152	Madagascar
MF058553	L2a1f3	Madagascar
MF058555	L0a2a2a	Madagascar
MF058557	L3e3b1	Madagascar
MF058559	L3e1a1a	Madagascar
MF058560	L1c3c	Madagascar
MF058561	L3e3a	Madagascar
MF381309	L1c1b	Angola
MF381310	L3d3a1a	Angola
MF381311	L3e1a2	Angola
MF381312	L3f1b4a	Angola
MF381313	L0a1b1	Angola
MF381314	L1c1b	Angola
MF381315	L3e1a2	Angola
MF381316	L1c1b	Angola
MF381317	L3d3a1a	Angola
MF381318	L0a1b1	Angola
MF381319	L0a1b1	Angola
MF381320	L0d1b1b1	Angola
MF381321	L3e1a2	Angola
MF381322	L3d3a1a	Angola
MF381323	L1c1b	Angola
MF381324	L3d3a1a	Angola
MF381325	L3e1a2	Angola
MF381326	L1c1b	Angola
MF381327	L1c1b	Angola
MF381328	L3e1a2	Angola
MF381329	L3e1a2	Angola
MF381330	L3d3a1a	Angola
MF381331	L3f1b4a	Angola
MF381332	L3e1a2	Angola

MF381333	L3d3a1a	Angola	MF381417	L3d3a1a	Angola
MF381334	L3e1a2	Angola	MF381418	L3f1b4a	Angola
MF381335	L0a1b1	Angola	MF381419	L0a1b1	Angola
MF381336	L3d3a1a	Angola	MF381420	L3f1b4a	Angola
MF381337	L3d3a1a	Angola	MF381421	L3f1b4a	Angola
MF381338	L0a2a1b	Angola	MF381422	L1c1b	Angola
MF381339	L3e1a2	Angola	MF381461	L0a2a1b	Angola
MF381340	L3d3a1a	Angola	MF381462	L3f1b4a	Angola
MF381341	L3d3a1a	Angola	MF381463	L3f1b4a	Angola
MF381342	L3d3a1a	Angola	MF381464	L0a1b1	Angola
MF381343	L3d3a1a	Angola	MF381465	L0a2a1b	Angola
MF381344	L3d3a1a	Angola	MF381466	L0a2a1b	Angola
MF381345	L3f1b4a	Angola	MF381467	L1c1b	Angola
MF381346	L3d3a1a	Angola	MF381468	L0a2a1b	Angola
MF381385	L1c1b	Angola	MF381469	L0a1b1	Angola
MF381386	L0a1b1	Angola	MF381470	L3d3a1a	Angola
MF381387	L1c1b	Angola	MF381471	L3d3a1a	Angola
MF381388	L1c2a1a	Angola	MF381472	L0d1b1b1	Angola
MF381389	L0d1b1b1	Angola	MF381473	L3f1b4a	Angola
MF381390	L1c2a1a	Angola	MF381474	L0a2a1b	Angola
MF381391	L3f1b4a	Angola	MF381475	L3d3a1a	Angola
MF381392	L0d1a1b1a	Angola	MF381476	L3d3a1a	Angola
MF381393	L0d1a1b1a	Angola	MF381477	L0a1b1	Angola
MF381394	L0d1b1b1	Angola	MF381478	L3d3a1a	Angola
MF381395	L3f1b4a	Angola	MF381479	L3f1b4a	Angola
MF381396	L0d1a1b1a	Angola	MF381480	L0a1b2	Angola
MF381397	L0d1a1b1a	Angola	MF381481	L3e2b	Angola
MF381398	L0d1b1b1	Angola	MF381482	L0a1b2	Angola
MF381399	L0d1a1b1a	Angola	MF381483	L0a1b2	Angola
MF381400	L0d1b1b1	Angola	MF381484	L0a2a1b	Angola
MF381401	L0d1a1b1a	Angola	MF381485	L3f1b4a	Angola
MF381402	L1c1b	Angola	MF381486	L0d1b1b1	Angola
MF381403	L3f1b4a	Angola	MF381487	L0d1a1b1a	Angola
MF381404	L3f1b4a	Angola	MF381488	L1c1b	Angola
MF381405	L0d1a1b1a	Angola	MF381489	L3f1b4a	Angola
MF381406	L0d1a1b1a	Angola	MF381490	L0a2a1b	Angola
MF381407	L0d1b1b1	Angola	MF381491	L1c1b	Angola
MF381408	L2a1c1	Angola	MF381492	L0d1b1b1	Angola
MF381409	L3e2b	Angola	MF381493	L0a2a1b	Angola
MF381410	L3e4a	Angola	MF381494	L0d1b1b1	Angola
MF381411	L0d1a1b1a	Angola	MF381495	L0d1b1+@152	Angola
MF381412	L0d1a1b1a	Angola	MF381496	L0a1b2	Angola
MF381413	L0d1a1b1a	Angola	MF381497	L0d1b1b1	Angola
MF381414	L3f1b4a	Angola	MF381498	L0d1b1b1	Angola
MF381415	L3e1a2	Angola	MF381537	L0d1c1a1a	Angola
MF381416	L3d3a1a	Angola	MF381538	L0d2a1a	Angola

MF381539	L0k1a1a	Angola	MF621104	L3h1a2b	Sudan
MF381540	L0k1a1b	Angola	MF621105	L3h1a2b	Sudan
MF381541	L0d2a1a	Angola	MF621106	L3h1b1a	Saudi Arabia
MF381542	L0d2a1a	Angola	MF621107	L3h1b2	Saudi Arabia
MF381543	L0d1b2b1a	Angola	MF621108	L4a1a	Saudi Arabia
MF381544	L0d2a1a	Angola	MF621109	L4a2	Saudi Arabia
MF381545	L0d1c1a1b	Angola	MF621110	L4a2	Rwanda
MF381546	L0d1c1a1a	Angola	MF621111	L4b1a	Ivory Coast
MF381547	L0d2a1a	Angola	MF621112	L4b1	Sudan
MF381548	L3d3a1	Angola	MF621113	L4b1	Sudan
MF381549	L0d1c1a1a	Angola	MF621114	L4b1	Kenya
MF381550	L0k1a1	Angola	MF621115	L4b1	Sudan
MF381551	L0d1c1a1a	Angola	MF621116	L4b1	Sudan
MF381552	L0k1a1	Angola	MF621117	L4b1	Sudan
MF381553	L0d1c1a1a	Angola	MF621118	L4b2a1	Saudi Arabia
MF381554	L0d1b1a	Angola	MF621119	L4b2b	Equatorial Guinea
MF381555	L0d2a1a	Angola	MF621120	L2a1c2a	Ghana
MF381556	L0d2a1a	Angola	MF621121	L2a1c3b	Sudan
MF381557	L0d2a1a	Angola	MF621122	L2a1+143+16189	Saudi Arabia
MF381558	L0d1c1a1a	Angola	MF621123	L2b1a3	Nigeria
MF381559	L0d1c1a1a	Angola	MF621124	L2d+16129	Mauritania
MF381560	L0d2a1a	Angola	MF621125	L5a1a	Tanzania
MF381561	L0d1c1a1b	Angola	MF621127	L1b2a	Nigeria
MF381562	L0k1a1a	Angola	MF621128	L1c1a1a1b1	Equatorial Guinea
MF381563	L0d2a1a	Angola	MF621129	L1c2a1b	Nigeria
MF381564	L0k1a1a	Angola	MF621130	L0a1b1	Morocco
MF381565	L0d2a1a	Angola	MF695863	L3e3a	Kenya
MF381566	L0k1a1b	Angola	MF695864	L0a2a1b	Kenya
MF381567	L0d1c1a1b	Angola	MF695905	L0a2a2a	Kenya
MF381568	L0d1c1a1a	Angola	MF695906	L2a1h	Kenya
MF381569	L0d2a1a	Angola	MF695907	L0a2a2a	Kenya
MF381570	L0d2a1a	Angola	MF695908	L1b1a	Kenya
MF381571	L0d1c1a1b	Angola	MF695909	L0a1'4	Kenya
MF381572	L0d1b2a1	Angola	MF695910	L3d1a1a1	Kenya
MF381573	L0d1c1a1a	Angola	MF695911	L0a	Kenya
MF381574	L0d1c1a1a	Angola	MF695912	L2a1+143	Kenya
MF621094	L3x2b	Spain	MF695913	L3d1a1a	Kenya
MF621095	L3x2a1a	Jordan	MF695914	L3d1a1a1	Kenya
MF621096	L3h1a1	Sudan	MF695915	L0a1b1a1a	Kenya
MF621097	L3h1a1	Sudan	MF695916	L2a1b1a	Kenya
MF621098	L3h1a1	Sudan	MF695917	L2a1b1a	Kenya
MF621099	L3h1a2a1	Tunisia	MF695918	L0a2	Kenya
MF621100	L3h1a2a1	Kenya	MF695919	L2a1+143	Kenya
MF621101	L3h1a2a1	Sudan	MF695920	L3d1a1a1	Kenya
MF621102	L3h1a2a1	Kenya	MF695921	L3e3a	Kenya
MF621103	L3h1a2a1	Sudan	MF695922	L4b2a2	Kenya

MF695923	L0a2a2a	Kenya
MF695924	L3e3a	Kenya
MF695925	L3e3a	Kenya
MF695926	L4b2a2	Kenya
MF695927	L3e3a	Kenya
MF695928	L4b2a2	Kenya
MF695929	L2b2a	Kenya
MF695930	L3d1a1a1	Kenya
MF695931	L0a2a2a	Kenya
MF695932	L0f2a	Kenya
MF695933	L3e1e1	Kenya
MF695934	L0a2a2a	Kenya
MF695936	L3e3a	Kenya
MF695937	L0a2a2a	Kenya
MF695938	L2a1b1a	Kenya
MF695939	L3e1d1	Kenya
MF695940	L3e3a	Kenya
MF695941	L3e1d1	Kenya
MF695942	L2a1h	Kenya
MF695943	L3e3a	Kenya
MF695983	L1c2a3	Kenya
MF695984	L3d1a1a	Kenya
MF695985	L0a2a2a	Kenya
MF695986	L0a	Kenya
MF695987	L3f	Kenya
MF695988	L3b1a1a	Kenya
MF695989	L4b2a2	Kenya
MF695991	L2a5	Kenya
MF695992	L3b1a2	Kenya
MF695993	L0f2a	Kenya
MF695994	L3b1a2	Kenya
MF695995	L2a1b1a	Kenya
MF695996	L3h1a2a1	Kenya
MF695997	L3d1a1a	Kenya
MF695998	L3e3a	Kenya
MF695999	L0f2a	Kenya
MF696000	L0f2a	Kenya
MF696001	L3d1a1a	Kenya
MF696003	L3b1a1a	Kenya
MF696004	L0a2a2a	Kenya
MF696005	L3f1b4a1	Kenya
MF696006	L1b1a	Kenya
MF696007	L3b1a1a	Kenya
MF696008	L3d1a1a	Kenya
MF696009	L3d1a1a	Kenya
MF696010	L3b1a1a	Kenya

MF696011	L0a2a2a	Kenya
MF696012	L3b1a1a	Kenya
MF696013	L3d1a1a1	Kenya
MF696014	L3d1a1a1	Kenya
MF696015	L0a2a2a	Kenya
MF696016	L3e1a2	Kenya
MF696017	L0a2a2a	Kenya
MF696018	L2a1h	Kenya
MF696019	L1b1a	Kenya
MF696020	L2a1b1a	Kenya
MF696021	L2a1h	Kenya
MF696022	L3e3a	Kenya
MF696062	L1b1a	Kenya
MF696063	L1b1a	Kenya
MF696064	L0f	Kenya
MF696065	L1b1a	Kenya
MF696066	L3e3a	Kenya
MF696067	L2a1+143+@16309	Kenya
MF696068	L4b2a2c	Kenya
MF696069	L0d3	Kenya
MF696070	L3d1a1a	Kenya
MF696071	L3h1b2	Kenya
MF696072	L1b1a	Kenya
MF696073	L3h1a2a1	Kenya
MF696074	L2a1f	Kenya
MF696075	L0a2a2a	Kenya
MF696076	L3x1a2	Kenya
MF696078	L0f2a	Kenya
MF696079	L3d1a1a	Kenya
MF696080	L0a2a2a	Kenya
MF696081	L0a2a2a	Kenya
MF696082	L3e1a2	Kenya
MF696083	L2a1b1a	Kenya
MF696085	L3d1a1a	Kenya
MF696086	L5b2	Kenya
MF696087	L3e2b	Kenya
MF696088	L0a2a2a	Kenya
MF696089	L3d1a1a	Kenya
MF696090	L0a2d	Kenya
MF696091	L0a2a1a	Comoros
MF696092	L1c2a1a	Comoros
MF696093	L1c2b2	Comoros
MF696094	L3d1a1a1	Comoros
MF696095	L3e3a	Comoros
MF696096	L2c2b1b	Comoros
MF696097	L2a5	Comoros

MF696098	L2a5	Comoros
MF696099	L3b1a1a	Comoros
MF696100	L2a1f3	Comoros
MF696102	L3d1a1a1	Comoros
MG022094	L3d1b2	USA
MG182028	L3e2b1	Algeria
MG182030	L2a1a	Algeria
MG201854	L0d1a1d	South Africa
MG561401	L3e2a	Sweden
MG571168	L3e1a1a	Peru
MG609035	L3f1b	USA
MH043563	L2c	Ibiza
MH043576	L2c	Ibiza
MH161386	L1b1a	USA
MH644539	L3e3b1	Brazil
MH681106	L3d6	USA
MH980013	L3b1a+@16124	Dominican Republic
MH981600	L2a1b1a	South Africa
MH981601	L0a2a2a	South Africa
MH981602	L0a1b1a1	South Africa
MH981603	L0a2a2a	South Africa
MH981604	L0d1c	South Africa
MH981605	L2a1b1a	South Africa
MH981606	L3d1a1a1	South Africa
MH981607	L0f1	South Africa
MH981608	L3b1a11	South Africa
MH981609	L2b2a	South Africa
MH981610	L1c2a3a	South Africa
MH981611	L0d1c	South Africa
MH981612	L3e2b1a2	South Africa
MH981613	L0a1b1a1	South Africa
MH981614	L0d1b2b1b	South Africa
MH981615	L2a1b1a	South Africa
MH981616	L1c1	South Africa
MH981618	L1c2a3a	South Africa
MH981619	L0a2a1a2	South Africa
MH981620	L3e1a1a	South Africa
MH981621	L0a2a2a	South Africa
MH981622	L0d1b2b2a	South Africa
MH981624	L0d1b2b1b	South Africa
MH981626	L0a2a2a	South Africa
MH981627	L3e2b1a2	South Africa
MH981671	L0d1b2b2a	South Africa
MH981672	L2a1b1a	South Africa
MH981673	L0a1b1a1	South Africa
MH981674	L0a2a2a	South Africa
MH981675	L3e1a3a	South Africa
MH981676	L2a1b1a	South Africa
MH981677	L0a2a2a	South Africa
MH981678	L0d2a1	South Africa
MH981679	L2a1a2a1a	South Africa
MH981680	L2a1b1a	South Africa
MH981681	L2a1h	South Africa
MH981682	L2a1b1a	South Africa
MH981684	L3e1b2	South Africa
MH981685	L1c2a3a	South Africa
MH981686	L3d1a1a1	South Africa
MH981836	L1c1a2	Paraguay
MH981841	L3e2a1b1	Paraguay
MH981849	L1b1a12a	Paraguay
MH981851	L2b1b	Paraguay
MH981854	L1c1b	Paraguay
MH981867	L3e1a1a	Paraguay
MH981878	L1b1a12a	Paraguay
MK049276	L3a2a	Somalia
MK139597	L1b1a+189	Spain
MK139598	L3b1a+@16124	Spain
MK139630	L3d1b3a	Spain
MK139649	L3b1a+@16124	Spain
MK228971	L2b2a	USA
MK295855	L0a1a1	Bulgaria
MK321329	L2a1l	Spain
MK484611	L4a1	Somalia
MK572004	L3h1b1a	Libya
MK732938	L2e	Uganda
NA18486	L1b1a	Nigeria
NA18487	L2a1l3	Nigeria
NA18488	L3e2b8	Nigeria
NA18489	L2a1a	Nigeria
NA18498	L1b1a3b	Nigeria
NA18917	L1b1a+189	Nigeria
NA18921	L2b2	Nigeria
NA18923	L1b1a3	Nigeria
NA18924	L1b1a3a	Nigeria
NA18933	L1b1a15	Nigeria
NA18934	L3e2a1b1	Nigeria
NA19017	L3b1a1a	Kenya
NA19019	L5a1b	Kenya
NA19020	L3e2a	Kenya
NA19022	L3b1a1a	Kenya
NA19023	L2a1f	Kenya
NA19024	L2b1a3	Kenya

NA19026	L3b1a1a	Kenya	NA19228	L2a1c2a	Nigeria
NA19027	L0a1a+200	Kenya	NA19229	L2c2	Nigeria
NA19028	L2a5	Kenya	NA19235	L3e2b+152	Nigeria
NA19030	L3d1a1a1	Kenya	NA19236	L3e2b+152	Nigeria
NA19031	L0b	Kenya	NA19238	L3e2b5	Nigeria
NA19035	L3e1a3a	Kenya	NA19239	L2a1p	Nigeria
NA19036	L3b1a1a	Kenya	NA19247	L2b	Nigeria
NA19037	L3h1a1	Kenya	NA19248	L2b3a	Nigeria
NA19038	L3h1a2a1	Kenya	NA19250	L1b1a3a1	Nigeria
NA19039	L0a3	Kenya	NA19253	L2b2a	Nigeria
NA19041	L1b1a	Kenya	NA19256	L1c3b2	Nigeria
NA19042	L0a1a+200	Kenya	NA19257	L3b1a10	Nigeria
NA19043	L5b2	Kenya	NA19259	L4b2b	Nigeria
NA19044	L3b1a1a	Kenya	NA19260	L3e3b	Nigeria
NA19045	L2a5	Kenya	NA19262	L3d1b2	Nigeria
NA19046	L2a5	Kenya	NA19266	L2a1a1	Nigeria
NA19092	L3e2a1b	Nigeria	NA19307	L3b1a1a	Kenya
NA19093	L2a1c5	Nigeria	NA19308	L5a1a	Kenya
NA19095	L2a1a2	Nigeria	NA19382	L0a1b1a1a	Kenya
NA19096	L2a1c3b2	Nigeria	NA19383	L4b1	Kenya
NA19098	L3b1a+152	Nigeria	NA19384	L3h1b1a	Kenya
NA19099	L2a1m1a	Nigeria	NA19385	L1c1d	Kenya
NA19102	L2a1a1	Nigeria	NA19390	L1c3b1a	Kenya
NA19107	L3b2a	Nigeria	NA19391	L3e1e	Kenya
NA19108	L2e1a	Nigeria	NA19392	L2b1a3	Kenya
NA19113	L3e2b+152	Nigeria	NA19393	L2a4b	Kenya
NA19187	L4b2b1	Nigeria	NA19395	L2a1f	Kenya
NA19189	L2a1f	Nigeria	NA19396	L3b1a1a	Kenya
NA19190	L1b1a15	Nigeria	NA19397	L3b1a1a	Kenya
NA19195	L3e2a1b2	Nigeria	NA19398	L3f2a1	Kenya
NA19196	L3e2a1b1	Nigeria	NA19399	L3e1e	Kenya
NA19197	L2b2	Nigeria	NA19401	L3x1a2	Kenya
NA19198	L1b1a15a	Nigeria	NA19402	L0a2a2a	Kenya
NA19200	L3e3b	Nigeria	NA19404	L5b1b	Kenya
NA19201	L2e1	Nigeria	NA19428	L3b1a1a	Kenya
NA19204	L3e2a	Nigeria	NA19429	L3b1a1a	Kenya
NA19207	L1b1a3	Nigeria	NA19430	L0a1a2	Kenya
NA19209	L2b1b	Nigeria	NA19431	L1b1a	Kenya
NA19210	L1b1a3	Nigeria	NA19432	L4b2a2c	Kenya
NA19213	L3f1b3	Nigeria	NA19434	L4b2a2c	Kenya
NA19214	L3e2a1b1	Nigeria	NA19435	L4b2a2c	Kenya
NA19216	L0a1a2	Nigeria	NA19436	L3b1a1a	Kenya
NA19217	L3e1	Nigeria	NA19437	L3i1	Kenya
NA19220	L3d1	Nigeria	NA19438	L3a2	Kenya
NA19222	L3b1a8	Nigeria	NA19439	L3b1a1a	Kenya
NA19223	L1b1a3	Nigeria	NA19440	L0a1c	Kenya

NA19443	L2a2b1	Kenya
NA19444	L4b2a2c	Kenya
NA19445	L4b2a2c	Kenya
NA19446	L3x1a1	Kenya
NA19448	L0a1b1a1a	Kenya
NA19449	L0a1b1a1a	Kenya
NA19451	L3b1a1a	Kenya
NA19452	L2a1a2	Kenya
NA19453	L4b2a2c	Kenya
NA19454	L0f1	Kenya
NA19921	L3e2a	USA
NA19922	L3d3a1	USA
NA19923	L2a1c3a1	USA
NA19984	L2b1a3	USA
NA19985	L0a2a2a1	USA
NA20126	L2a1f1	USA
NA20127	L2c5	USA
NA20274	L2a1b+143	USA
NA20276	L2a1a3a	USA
NA20278	L3e2a1b1	USA
NA20281	L1c1d1	USA
NA20282	L1b1a	USA
NA20287	L2a1a2a1	USA
NA20289	L1b1a4a	USA
NA20291	L3e4a	USA
NA20294	L3d1b3a	USA
NA20296	L3d4	USA
NA20298	L3k	USA
NA20317	L3d4	USA
NA20318	L3d4	USA
NA20320	L1b1a4	USA
NA20321	L1b1a4	USA
NA20322	L1b1a4	USA
NA20332	L2a1l1b	USA
NA20334	L0a1a2	USA
NA20336	L0a1a2	USA
NA20339	L3d1c1	USA
NA20340	L2a1a1	USA
NA20341	L1b1a4a	USA
NA20342	L3e2b+152	USA
NA20344	L3e2b1a1	USA
NA20346	L3e2b1a1	USA
NA20348	L3e2b1a1	USA
NA20351	L2a1c1a2	USA
NA20355	L0a1a2	USA
NA20357	L2a1e1	USA
NA20359	L1c3a	USA
NA20362	L1c3a	USA
AY195784	L3b1a	South Africa
AY195785	L2c5	South Africa
AY195788	L2a2b1a	South Africa
AY195789	L1c1a1a1b1	South Africa
AY963585	L0f2a1	Uganda
DQ282505	L1b1a7a	USA
DQ282506	L1b1a7a	USA
DQ282507	L3e1e2	USA
DQ304897	L0a1b2	USA
DQ304898	L0a1b2	USA
DQ304899	L0a1b1a	USA
DQ304900	L0a1b1	USA
DQ304901	L0a1a2	USA
DQ304902	L0a1a2	USA
DQ304903	L0a1a2	USA
DQ304904	L0a1a2	USA
DQ304905	L1b1a3a	USA
DQ304906	L1b1a3a1	USA
DQ304907	L1b1a3b	USA
DQ304908	L1b1a3	USA
DQ304909	L1b1a3	USA
DQ304910	L1b1a3	USA
DQ304911	L1b1a3a1	USA
DQ304912	L1b1a3a	USA
DQ304913	L1b1a3a	USA
DQ304914	L1b1a3a	USA
DQ304915	L1b1a3a	USA
DQ304916	L1b1a3	USA
DQ304917	L1b1a3	USA
DQ304918	L1b1a3a1	USA
DQ304919	L1b1a	USA
DQ304920	L1b1a	USA
DQ304921	L1b1a7	USA
DQ304922	L1b1a	USA
DQ304923	L1b1a7	USA
DQ304924	L2a1a	USA
DQ304963	L2a1f1	USA
DQ304964	L2a1f1	USA
DQ304965	L2a1f	USA
DQ304966	L2a1f	USA
DQ304967	L2a1f1	USA
DQ304968	L2a1a2	USA
DQ304969	L2a1a2a1a	USA
DQ304970	L2a1a2a1a	USA

DQ304971	L2a1a2	USA	DQ341076	L3f2a1a	Ethiopia
DQ304972	L2a1a2b	USA	DQ341077	L3f1b+16292	Ethiopia
DQ304973	L2a1a2b	USA	DQ341078	L3f1a1	Ethiopia
DQ304974	L2a1a2a1a	USA	DQ341079	L3h1b1	Ethiopia
DQ304975	L2a1a2a	USA	DQ341080	L3h2	Ethiopia
DQ304976	L2a1a2a1a	USA	DQ341081	L3a1b	Ethiopia
DQ304977	L2a1a2	USA	EF177417	L2a1c6	Portugal
DQ304978	L2b1a3	USA	EF184582	L5b1	Tanzania
DQ304979	L2b1a3	USA	EF184585	L0d3a	Tanzania
DQ304980	L2b1a3	USA	EF184586	L0d1b2a1	Tanzania
DQ304981	L2b1a	USA	EF184587	L0d3a	Tanzania
DQ304982	L2b1a3	USA	EF184588	L0d3a	Tanzania
DQ304983	L2b1a3	USA	EF184589	L0d3a	Tanzania
DQ304984	L2b1a3	USA	EF184590	L0d1b2a1	South Africa
DQ304985	L2b1a2	USA	EF184591	L0d2c1	South Africa
DQ304986	L2c2	USA	EF184592	L0d1c1a1a	South Africa
DQ304987	L2c2a1	USA	EF184593	L0d1c3	South Africa
DQ304988	L2c2a	USA	EF184594	L0d1c1a1b	South Africa
DQ304989	L2c2	USA	EF184595	L0f	Tanzania
DQ304990	L3b1a3	USA	EF184596	L0f2a	Tanzania
DQ304991	L3b1a11	USA	EF184597	L0f2a1	Tanzania
DQ304992	L3b1a11	USA	EF184598	L0f	Tanzania
DQ304993	L3b1a5	USA	EF556171	L3x1a	Israel
DQ304994	L3b1a1a	USA	EF556173	L5a1a	Israel
DQ304995	L3b1a1a	USA	EF556174	L0a2c	Israel
DQ304996	L3b1a	USA	ESP0167	L3e4a	Spain
DQ304997	L3b1a	USA	ESP0191	L3d1b3a	Spain
DQ304998	L3e2b+152	USA	ESP0225	L2a1c1	Spain
DQ304999	L3e2b+152	USA	ESP0297	L3e5a	Spain
DQ305000	L3e2b4	USA	ESP0313	L2a1c3a	Spain
DQ341060	L5a1a	Ethiopia	ESP0413	L4b2b	Spain
DQ341061	L5b1a	Ethiopia	ESP0702	L1b1a+189	Spain
DQ341062	L2d1a	Ethiopia	ESP0714	L3b1a+@16124	Spain
DQ341063	L6b	Ethiopia	ESP0813	L1b1a6	Spain
DQ341064	L4a1a	Ethiopia	ESP0922	L3b1b1	Spain
DQ341065	L4b2a1	Ethiopia	ESP0926	L2c5	Spain
DQ341066	L3x2a1	Ethiopia	ESP0928	L2a1c+16129	Spain
DQ341067	L3x1b	Ethiopia	ESP0954	L1b1a14	Spain
DQ341068	L3i2	Ethiopia	ESP0969	L1b1a6	Spain
DQ341069	L3i1b	Ethiopia	ESP0975	L3e2b	Spain
DQ341070	L3e5e	Ethiopia	ESP1024	L3e1f	Spain
DQ341071	L3e2b	Ethiopia	EU092658	L2a1+143+16189	Israel
DQ341072	L3d1b1	Ethiopia	EU092659	L2a1+143+16189	Israel
DQ341073	L3b1a7a	Ethiopia	EU092660	L3c	Israel
DQ341074	L3c	Ethiopia	EU092661	L2b3c	Israel
DQ341075	L3f3b	Ethiopia	EU092662	L4b2a2a	Israel

EU092663	L2a1c3a	Israel
EU092664	L2b1a2	Israel
EU092665	L0a1a	Israel
EU092666	L3x1+16311	Israel
EU092667	L1b1a16	Israel
EU092668	L0f2a1	Israel
EU092669	L3b1a2	Israel
EU092670	L0a1d	Israel
EU092671	L2a1p	Israel
EU092672	L1b1a2	Israel
EU092673	L6b	Israel
EU092674	L2a1+143+16189	Israel
EU092675	L3e1b1	Israel
EU092676	L2a1h	Israel
EU092715	L1b1a4a	Guinea-Bissau
EU092716	L1b1a6	Guinea-Bissau
EU092717	L1c1c	Guinea-Bissau
EU092718	L1c3a1a	Guinea-Bissau
EU092719	L2a1i	Guinea-Bissau
EU092720	L2a1c3b1	Guinea-Bissau
EU092721	L2a1l2	Guinea-Bissau
EU092722	L2b1a2	Guinea-Bissau
EU092723	L2c	Guinea-Bissau
EU092724	L2e	Guinea-Bissau
EU092725	L3b2a	Guinea-Bissau
EU092726	L3b1a	Guinea-Bissau
EU092727	L3b1a9a	Guinea-Bissau
EU092728	L3d1b1	Guinea-Bissau
EU092729	L3e2a3	Guinea-Bissau
EU092730	L3e2b	Guinea-Bissau
EU092731	L3e4a1	Guinea-Bissau
EU092732	L3f1b+16292	Guinea-Bissau
EU092733	L2a1c	Guinea-Bissau
EU092734	L2b3b	Guinea-Bissau
EU092735	L3d2b	Guinea-Bissau
EU092736	L3h1b2	Guinea-Bissau
EU092737	L1b1a9	Syria
EU092738	L1c2b2	Syria
EU092739	L2a1o	Syria
EU092740	L3e1c	Syria
EU092741	L3f1b+16292	Syria
EU092742	L3d4a	Syria
EU092743	L4b2a2a	Syria
EU092744	L3b1a2	Syria
EU092745	L0a2a2a	Saudi Arabia
EU092746	L0a1b2a	Saudi Arabia

EU092747	L2b1	Saudi Arabia
EU092748	L4a1a	Saudi Arabia
EU092749	L3e1a1a	Saudi Arabia
EU092750	L4b2a2	Saudi Arabia
EU092751	L3f1b+16292	Lebanon
EU092752	L3e4	Lebanon
EU092791	L3f1b4a1	Oman
EU092792	L0k2b	Yemen
EU092793	L2a1+143+16189	Yemen
EU092794	L2d1a	Yemen
EU092795	L3b1a2	Yemen
EU092796	L3d1a	Yemen
EU092797	L3d1a1a	Yemen
EU092798	L3h1a2a1	Yemen
EU092799	L4a2	Yemen
EU092800	L4a2	Yemen
EU092801	L0a1d	Yemen
EU092802	L6a	Yemen
EU092803	L6a	Yemen
EU092804	L2a1a2	Yemen
EU092805	L3f1b2	Yemen
EU092806	L2a1+16189	Morocco
EU092807	L2a1l1a	Morocco
EU092808	L4b1a	Yemen
EU092809	L0a1d	Yemen
EU092810	L0a1d	Yemen
EU092811	L1c3b1a	Morocco
EU092812	L2a1l1a1	Morocco
EU092813	L2c1a	Morocco
EU092814	L3b1b	Morocco
EU092815	L3b1a+152	Morocco
EU092816	L2a1j	Morocco
EU092817	L2d+16129	Algeria
EU092818	L3x2b	Algeria
EU092819	L0a1a2	Algeria
EU092820	L3b1a	Libya
EU092821	L3e5a1	Libya
EU092822	L3k	Libya
EU092823	L2a1+143+16189	Libya
EU092824	L3k1	Tunisia
EU092825	L3b1a9a	Tunisia
EU092826	L3b1a3	Tunisia
EU092827	L3e1	Tunisia
EU092828	L3h1b1	Tunisia
EU092867	L3e1a2	South Africa
EU092868	L0a2a1a2	South Africa

EU092869	L0a1b1a1	South Africa
EU092870	L0f1	South Africa
EU092871	L0a1b1a1	South Africa
EU092872	L2a1a2a1a	South Africa
EU092873	L1c2a1a	South Africa
EU092874	L0f1	South Africa
EU092875	L3e1a2	South Africa
EU092876	L3d1a1b	South Africa
EU092877	L3f2b	South Africa
EU092878	L0a1b	Chad
EU092879	L3e1c	Chad
EU092880	L3e1c	Chad
EU092881	L0a1a+200	Chad
EU092882	L2a2a1	Chad
EU092883	L3f1b3	Chad
EU092884	L1b1a10a	Chad
EU092885	L3e1c	Chad
EU092886	L3h1b1a	Chad
EU092887	L3e1	Chad
EU092888	L5a1b	Chad
EU092889	L0a1b	Chad
EU092890	L2a1a3a	Chad
EU092891	L3f1a	Chad
EU092892	L0a1a+200	Chad
EU092893	L1b1a10a	Chad
EU092894	L3f3a	Chad
EU092895	L3e3b	Chad
EU092896	L2a2a	Chad
EU092897	L3b1a10	Chad
EU092898	L3d1'2'3'4'5'6	Chad
EU092899	L3d1b	Chad
EU092900	L0a3	Chad
EU092901	L2a1c	Chad
EU092902	L2a2a1	Chad
EU092903	L3h1b1a	Chad
EU092904	L3b1a	Chad
EU092943	L5a1b	Ethiopia
EU092944	L3x2a	Ethiopia
EU092945	L0a1c1	Ethiopia
EU092946	L2a1+143	Ethiopia
EU092947	L3h1b1a	Ethiopia
EU092948	L1b1a2a	Ethiopia
EU092949	L4a2	Ethiopia
EU092950	L0a1d	Ethiopia
EU092951	L4b2a2b	Ethiopia
EU092952	L1b1a2a	Ethiopia
EU092953	L3f1b2a	Ethiopia
EU092954	L2a1c1a2	Ethiopia
EU092955	L2c2a	Unknown
EU092956	L1c3a1b	Unknown
EU092957	L2c2a	Unknown
EU092958	L3b1a6	Unknown
EU092959	L3e5b	Unknown
EU092960	L3e1a3b	Gambia
EU092961	L2a1f1	Unknown
EU092962	L3b1a5	Unknown
EU092963	L0a1b2	Unknown
EU092964	L0f2a	Unknown
EU092965	L0d1b2b1a	Unknown
EU092966	L0k1a1a	Unknown
EU200759	L3d1b1b	Poland
EU200760	L2a1k	Czech Rep
EU200761	L3b1b1	Russia
EU200762	L2a1c3a	Slovenia
EU200763	L2a1k	Slovenia
EU200764	L1b1a12b	Russia
EU273476	L1c1a2b	Cameroon
EU273477	L1c1a1a1a	Cameroon
EU273478	L1c1a1a1b1	Cameroon
EU273479	L1c1a2a1	Gabon
EU273480	L1c1a1a1b	Gabon
EU273481	L1c1a1a1b	Gabon
EU273482	L1c1a1a1b1	Gabon
EU273483	L1c1a1a1a	Gabon
EU935437	L0a1b1a	Egypt
EU935440	L3b2a	Egypt
EU935443	L2a1c5	Egypt
EU935449	L3b1a2	Egypt
EU935451	L3f1b2a	Egypt
EU935458	L1c3a1a	Egypt
EU935462	L1c3a1a	Egypt
EU935464	L0a1b1a	Egypt
EU935465	L3e2b7	Egypt
EU935467	L0a1b1a	Egypt
FJ157838	L0a2a2	India
FJ157839	L0a2a2	India
FJ157840	L0a2a2	India
FJ228403	L2b1b	Senegal
FJ460520	L2a1+143+@16309	Tunisia
FJ460522	L1b1a13	Tunisia
FJ460523	L2e1a	Tunisia
FJ460526	L2b3c	Tunisia

FJ460527	L2a1+143+16189	Tunisia
FJ460529	L3b1a3	Tunisia
FJ460531	L4a1	Tunisia
FJ460533	L3e5d	Tunisia
FJ460535	L2b1a3	Tunisia
FJ460536	L3b1a4	Tunisia
FJ460537	L1b1a12a	Tunisia
FJ460540	L3d4a	Tunisia
FJ460549	L2a1c4a	Tunisia
FJ460560	L2a1c1	Tunisia
FJ625845	L3f1a1	Chad
FJ625846	L3f3	Chad
FJ625847	L3f3	Chad
FJ625848	L3f3	Chad
FJ625849	L3f3	Chad
FJ625850	L3f3b	Chad
FJ625851	L3f3	Chad
FJ625852	L3f3b	Chad
FJ625853	L3f3	Chad
FJ625854	L3f3a	Chad
HG01456	L3e2a1b3	Colombia
HG01551	L3e4a	Colombia
HG01607	L1b1a6	Spain
HG01761	L3d1b1	Spain
HG02006	L3e3b1	Peru
HG02304	L3f1b+16292+150	Peru
HG02439	L2a1a2b	Barbados
HG02455	L2a1c5	Barbados
HG02461	L3b1a	Gambia
HG02462	L2c5	Gambia
HG02464	L3e4a	Gambia
HG02465	L3b2a	Gambia
HG02476	L2a1f1	Barbados
HG02477	L1b1a9	Barbados
HG02481	L2a1f2	Barbados
HG02501	L2a1a	Barbados
HG02502	L2a1c	Barbados
HG02505	L2c1	Barbados
HG02536	L2d+16129	Barbados
HG02541	L3f1b4a	Barbados
HG02545	L2d1	Barbados
HG02546	L2a1c5	Barbados
HG02549	L2a1c5	Barbados
HG02554	L2a1i1	Barbados
HG02555	L2b	Barbados
HG02557	L2a1f1	Barbados
HG02558	L3d1'2'3'4'5'6	Barbados
HG02561	L1b2	Gambia
HG02562	L1b1a7a	Gambia
HG02568	L2a1l	Gambia
HG02570	L4b1a	Gambia
HG02571	L1b1a14	Gambia
HG02573	L3e2a3	Gambia
HG02574	L3d2a	Gambia
HG02577	L2a1b1	Barbados
HG02580	L1b1a15	Barbados
HG02582	L3k1	Gambia
HG02583	L3d1a1b	Gambia
HG02769	L3e4a1	Gambia
HG02771	L3d1'2'3'4'5'6	Gambia
HG02772	L1b1a7	Gambia
HG02798	L3d1a1b	Gambia
HG02799	L2d+16129	Gambia
HG02804	L2b	Gambia
HG02805	L3h1b2	Gambia
HG02807	L3e2b	Gambia
HG02808	L2a1c	Gambia
HG02810	L3b1a5	Gambia
HG02811	L1b1a4	Gambia
HG02813	L3b2a	Gambia
HG02814	L2c	Gambia
HG02817	L3e3b	Gambia
HG02819	L2a1c2	Gambia
HG02820	L1b2a	Gambia
HG02836	L2c2	Gambia
HG02837	L2b	Gambia
HG02839	L3e2a	Gambia
HG02840	L1b1a4	Gambia
HG02851	L2a1c3b	Gambia
HG02852	L2a1a	Gambia
HG02854	L3b1a+@16124	Gambia
HG02855	L1b1a4	Gambia
HG02860	L2a1a	Gambia
HG02861	L2a1c3a1	Gambia
HG02870	L2a1a3	Gambia
HG02878	L2c4	Gambia
HG02879	L2c	Gambia
HG02881	L2d+16129	Gambia
HG02882	L2c	Gambia
HG02885	L2a1f	Gambia
HG02887	L2b1a2	Gambia
HG02888	L2c	Gambia

HG02890	L3e2a3	Gambia	HG03298	L0a1a2	Nigeria
HG02891	L1b1a4	Gambia	HG03300	L1c2b1a'b	Nigeria
HG02895	L2a1c3a1	Gambia	HG03301	L3e2a1b1	Nigeria
HG02896	L2c5	Gambia	HG03303	L3f1b4c	Nigeria
HG03066	L1b2a	Sierra Leone	HG03304	L3e2b8	Nigeria
HG03069	L2c2	Sierra Leone	HG03311	L1b1a	Nigeria
HG03072	L2c	Sierra Leone	HG03313	L1c2a2	Nigeria
HG03073	L1b1a9	Sierra Leone	HG03343	L1c3a	Nigeria
HG03074	L2c3	Sierra Leone	HG03351	L1b1a	Nigeria
HG03077	L1c1d	Sierra Leone	HG03352	L3e2b1a1	Nigeria
HG03078	L2a1c	Sierra Leone	HG03354	L2c2b1a	Nigeria
HG03079	L1b1a18	Sierra Leone	HG03363	L1c2b1b	Nigeria
HG03081	L3e4a	Sierra Leone	HG03366	L3d1b2	Nigeria
HG03082	L3e3b	Sierra Leone	HG03367	L2e1a	Nigeria
HG03084	L0a1a3	Sierra Leone	HG03369	L3e3b	Nigeria
HG03085	L2a1l	Sierra Leone	HG03370	L3b1a	Nigeria
HG03086	L3b3	Sierra Leone	HG03372	L3e3b	Nigeria
HG03088	L2a1c3b1	Sierra Leone	HG03376	L2c	Sierra Leone
HG03091	L2c	Sierra Leone	HG03378	L3e3b	Sierra Leone
HG03095	L3b1a5	Sierra Leone	HG03380	L1c1c	Sierra Leone
HG03096	L1c1c	Sierra Leone	HG03382	L2a1l1b	Sierra Leone
HG03097	L2a1l	Sierra Leone	HG03385	L1c1c	Sierra Leone
HG03099	L1b1a+189	Nigeria	HG03388	L2c2	Sierra Leone
HG03100	L3d1d	Nigeria	HG03391	L1c1c	Sierra Leone
HG03103	L1b1a15	Nigeria	HG03394	L3b1a6	Sierra Leone
HG03105	L3e3b	Nigeria	HG03397	L1c1d	Sierra Leone
HG03108	L3b1a	Nigeria	HG03401	L2a1l	Sierra Leone
HG03109	L1b1a3	Nigeria	HG03410	L2a1a1	Sierra Leone
HG03111	L3f1b3	Nigeria	HG03419	L3e2b+152	Sierra Leone
HG03112	L1b1a3	Nigeria	HG03428	L2a1l1b	Sierra Leone
HG03114	L2a1a2c	Nigeria	HG03432	L2a1i	Sierra Leone
HG03115	L3e2b1a2	Nigeria	HG03433	L2c	Sierra Leone
HG03117	L2a1c1a2	Nigeria	HG03436	L1b1a14	Sierra Leone
HG03118	L3e1a	Nigeria	HG03437	L2d1	Sierra Leone
HG03120	L1b1a3	Nigeria	HG03572	L3e2b	Sierra Leone
HG03123	L2a1a2	Nigeria	HG03575	L2a1f1	Sierra Leone
HG03124	L3b1a	Nigeria	HG03577	L3e2a	Sierra Leone
HG03126	L3e1a3b	Nigeria	HG03578	L2a1a1	Sierra Leone
HG03127	L0a1a2	Nigeria	HG03583	L3h1b1a	Sierra Leone
HG03129	L3d2a	Nigeria	HM596745	L2a5	Bermuda
HG03130	L2e1a	Nigeria	HM771113	L1c1a2c	Congo
HG03132	L2a1b1	Nigeria	HM771114	L1c1a2c	Congo
HG03291	L2b1a	Nigeria	HM771115	L1c1a2c	Congo
HG03294	L3b1b	Nigeria	HM771116	L1c4b	Congo
HG03295	L1b1a	Nigeria	HM771117	L1c4a	Congo
HG03297	L2a1f1	Nigeria	HM771118	L1c4b	Congo

HM771119	L1c4b	Congo
HM771120	L1c4b	Congo
HM771121	L1c4b	Congo
HM771122	L3e2b	Gabon
HM771123	L1c1a2b	Cameroon
HM771124	L1c1a2b	Cameroon
HM771125	L1c1b1	Cameroon
HM771126	L1c4a	Cameroon
HM771127	L1c1a1a1a	Cameroon
HM771128	L1c1a1a1b	Congo
HM771129	L1c1a1a1b	Congo
HM771130	L1c1a1a1b1	Congo
HM771131	L1c1a1a1b1	Cameroon
HM771132	L1c1a1a1a	Cameroon
HM771133	L1c1a1a1a	Congo
HM771134	L1c1a1a1a	Cameroon
HM771135	L3e1f1	Cameroon
HM771136	L1c1a2a1	Gabon
HM771137	L1c1a2b	Gabon
HM771138	L1c1a2b	Congo
HM771139	L1c1a1a1b1	Congo
HM771140	L1c1a1a1b	Congo
HM771141	L1c1a1a1a	Gabon
HM771142	L1c1a2b	Cameroon
HM771143	L1c1a2b	Cameroon
HM771144	L1c1a1a1b1	Cameroon
HM771183	L1c1a1a1b	Central African Republic
HM771184	L1c1a1a1b	Central African Republic
HM771185	L1c1a1a1b	Central African Republic
HM771186	L1c1a1a1b1	Central African Republic
HM771187	L1c1a1a1b1	Central African Republic
HM771188	L0a2b1	Central African Republic
HM771189	L0a2b	Central African Republic
HM771190	L0a2b	Central African Republic
HM771191	L2a2b2	Congo
HM771192	L2a2b1a	Congo
HM771193	L2a2b1a	Congo
HM771194	L2a2b1a	Congo
HM771195	L2a2b1a	Congo
HM771196	L2a4a	Congo
HM771197	L2a4a	Congo
HM771198	L5a1c	Unknown
HM771199	L0a2b	Central African Republic
HM771200	L0a2b1	Central African Republic
HM771201	L0a2b1	Central African Republic
HM771202	L0a2b	Central African Republic
HM771203	L5a1c	Unknown
HM771204	L5a1c	Unknown
HM771205	L2a2a1	Congo
HM771206	L2a3	Congo
HM771207	L2a2b2	Congo
HM771208	L2a2b1a	Congo
HM771209	L2a4a	Congo
HM771210	L2a4a	Congo
HM771211	L2a4a	Congo
HM771212	L2a4a	Congo
HM771213	L2a4a	Congo
HM771214	L2a4a	Congo
HM771215	L2a4a	Congo
HM771216	L1c1a2b	Gabon
HM771217	L1c1a2a1	Gabon
HM771218	L1c1a2a1	Gabon
HM771219	L1c3a1b	Gabon
HM771220	L1c1b1	Gabon
JN214448	L3e1f2	Italy
JN214449	L2a1a2	Italy
JN214450	L3d1b1a	Italy
JN214451	L3b3	Italy
JN214452	L3e5a	Hungary
JN214453	L2b1a4	Italy
JN214454	L2b1a4	Italy
JN214457	L2a1a2	Italy
JN214458	L3h1b1a	Spain
JN214459	L3d1b1a	Italy
JN214460	L1b1a16	Italy
JN214461	L1b1a6	Portugal
JN214462	L1b1a3	Portugal
JN214463	L1b1a12a	Portugal
JN214464	L1b1a	England
JN214465	L1b1a+189	Ireland
JN214466	L1b1a	Portugal
JN214467	L1b1a6	Wales
JN214468	L1b1a14	France
JN214469	L1b1a+189	Slovenia
JN214470	L1b2a	Germany
JN214471	L1b1a+189	Switzerland

JN214472	L1b1a6	Portugal	JN655841	L3f2a1	Sudan
JN214473	L1b1a14	Italy	JN655842	L3f1a1	Sudan
JN214474	L1b1a2a	Spain	JN858955	L2a1c+16129	Cameroon
JN214475	L3e2b	Italy	JN858956	L2a1d2	Benin
JN214476	L1b1a16	France	JN989561	L2a1f1	unknown
JN214477	L1b1a13	Italy	JQ044792	L1b1a4a	Burkina Faso
JN214478	L3d1b1a	Italy	JQ044793	L1b1a10	Burkina Faso
JN214479	L3d	Italy	JQ044794	L3e2b1a1	Burkina Faso
JN214480	L1b1a	Italy	JQ044835	L3e3b	Burkina Faso
JN225464	L3e2b	Italy	JQ044836	L1c6	Burkina Faso
JN225465	L1b1a16	Italy	JQ044837	L2a1b3	Burkina Faso
JN225466	L1b1a16	Italy	JQ044838	L0a1a2	Burkina Faso
JN225467	L3d1b1a	Italy	JQ044839	L2a1+143+@16309	Burkina Faso
JN225468	L3d1b1a	Italy	JQ044840	L3e3'4'5	Burkina Faso
JN381504	L3e2b4	USA	JQ044841	L2a1b2	Burkina Faso
JN415484	L2a1o	France	JQ044842	L3d1c1	Burkina Faso
JN655811	L3e1d1	Somalia	JQ044843	L3b2a	Burkina Faso
JN655812	L3e3a	Somalia	JQ044844	L2a1c2	Burkina Faso
JN655813	L3a1a	Somalia	JQ044845	L2a1a	Burkina Faso
JN655814	L3i2	Somalia	JQ044846	L2b2a	Burkina Faso
JN655815	L3h2	Somalia	JQ044847	L3e1g	Burkina Faso
JN655816	L3i2	Somalia	JQ044848	L4b1a	Burkina Faso
JN655817	L3f1a1	Somalia	JQ044849	L0a1a2	Burkina Faso
JN655818	L3x1b	Somalia	JQ044850	L3e3b3	Burkina Faso
JN655819	L3f2a1a	Somalia	JQ044851	L0a1a+200	Burkina Faso
JN655820	L3h2	Yemen	JQ044852	L3d1c1	Burkina Faso
JN655821	L3h2	Yemen	JQ044853	L2c2b2	Burkina Faso
JN655822	L3e5	Sudan	JQ044854	L2b1a	Burkina Faso
JN655823	L3d1b1b	Sudan	JQ044855	L1c3a1b	Burkina Faso
JN655824	L3h1a2a1	Sudan	JQ044856	L3e2b	Burkina Faso
JN655825	L3h2	Sudan	JQ044857	L1b1a	Burkina Faso
JN655826	L3b1a+@16124	Sudan	JQ044858	L2c1	Burkina Faso
JN655827	L3b1a2	Sudan	JQ044859	L2a1f	Burkina Faso
JN655828	L3e5+195	Sudan	JQ044860	L2a1c1	Burkina Faso
JN655829	L3x2a1a	Sudan	JQ044861	L2a1f3	Burkina Faso
JN655830	L3h1a1	Sudan	JQ044862	L3d1c	Burkina Faso
JN655831	L3f3	Sudan	JQ044863	L1b1a4	Burkina Faso
JN655832	L3f2a1	Sudan	JQ044864	L3d5a	Burkina Faso
JN655833	L3f1b+16292	Sudan	JQ044865	L3e2b	Burkina Faso
JN655834	L3d4a	Sudan	JQ044866	L1b2	Burkina Faso
JN655835	L3f1b+16292	Sudan	JQ044867	L1b1a10	Burkina Faso
JN655836	L3f1b+16292	Sudan	JQ044868	L1b1a+189	Burkina Faso
JN655837	L3x1+16311	Sudan	JQ044869	L3b2b	Burkina Faso
JN655838	L3h1b1a	Sudan	JQ044870	L3e3b3	Burkina Faso
JN655839	L3h1b1a	Sudan	JQ044871	L3e2a	Burkina Faso
JN655840	L3h1a2a	Sudan	JQ044872	L2a1c2a	Burkina Faso

JQ044913	L3e2b	Burkina Faso
JQ044914	L2c4	Burkina Faso
JQ044915	L3d1c1	Burkina Faso
JQ044916	L2a1m	Burkina Faso
JQ044917	L2c3	Burkina Faso
JQ044918	L2a1a3b	Burkina Faso
JQ044919	L2a1l1	Burkina Faso
JQ044920	L2c2	Burkina Faso
JQ044921	L2c4	Burkina Faso
JQ044922	L2a1a	Burkina Faso
JQ044924	L2a1c2	Burkina Faso
JQ044925	L3e3b	Burkina Faso
JQ044926	L1b1a	Burkina Faso
JQ044927	L2a1a	Burkina Faso
JQ044928	L3e2	Burkina Faso
JQ044929	L2d1a	Burkina Faso
JQ044930	L2a1a	Burkina Faso
JQ044931	L3e2b1	Burkina Faso
JQ044932	L2a1l1a	Burkina Faso
JQ044933	L3e2b	Burkina Faso
JQ044935	L2a1c2	Burkina Faso
JQ044936	L1b3	Burkina Faso
JQ044937	L2a1c4a1	Burkina Faso
JQ044938	L3e2	Burkina Faso
JQ044939	L3d1b2	Burkina Faso
JQ044941	L2c	Burkina Faso
JQ044942	L2a1a	Burkina Faso
JQ044943	L0a1a3	Burkina Faso
JQ044944	L2a1n	Burkina Faso
JQ044945	L2a1a2	Burkina Faso
JQ044947	L3e3b	Burkina Faso
JQ044948	L2d1	Burkina Faso
JQ044949	L1b1a9	Burkina Faso
JQ044950	L1b1a+189	Burkina Faso
JQ044951	L2a1a2	Burkina Faso
JQ044952	L2a1a2	Burkina Faso
JQ044953	L1b1a9	Burkina Faso
JQ044954	L1b2	Burkina Faso
JQ044993	L3b1a+152	Burkina Faso
JQ044994	L2a1l2	Burkina Faso
JQ044995	L0a1a2	Burkina Faso
JQ044996	L2a1c3b	Burkina Faso
JQ044997	L2a1a2	Burkina Faso
JQ044998	L2a1c2	Burkina Faso
JQ044999	L3e3b	Burkina Faso
JQ045000	L2a1o	Burkina Faso
JQ045001	L2a1a1	Burkina Faso
JQ045002	L2c1	Burkina Faso
JQ045004	L0a1a2	Burkina Faso
JQ045005	L2a1a	Burkina Faso
JQ045006	L2a1f	Burkina Faso
JQ045008	L2b	Burkina Faso
JQ045009	L3b1a	Burkina Faso
JQ045010	L2c2	Burkina Faso
JQ045011	L2d1a	Burkina Faso
JQ045012	L2b3a	Burkina Faso
JQ045013	L2b1a2	Burkina Faso
JQ045014	L2a1c4a	Burkina Faso
JQ045015	L2a1a3	Burkina Faso
JQ045016	L3e2b	Burkina Faso
JQ045017	L2a1a2a1a	Burkina Faso
JQ045018	L3b1a9	Burkina Faso
JQ045019	L2a1c	Burkina Faso
JQ045020	L2a1a1	Burkina Faso
JQ045021	L2a1a	Burkina Faso
JQ045022	L2c1a	Burkina Faso
JQ045023	L3b1a9	Burkina Faso
JQ045024	L2a1c2	Burkina Faso
JQ045025	L2a1c2a	Burkina Faso
JQ045026	L3d4	Burkina Faso
JQ045027	L2a1a2	Burkina Faso
JQ045028	L3b1a6	Burkina Faso
JQ045029	L3b2	Burkina Faso
JQ045030	L2c	Burkina Faso
JQ045031	L3e1	Burkina Faso
JQ045032	L1b1a3a1	Burkina Faso
JQ045071	L1b2	Burkina Faso
JQ045072	L3b1a9a	Burkina Faso
JQ045073	L1c3a1a	Burkina Faso
JQ045074	L2a1a1	Burkina Faso
JQ045075	L3d1b	Burkina Faso
JQ045076	L2a1a2	Burkina Faso
JQ045077	L2a1i	Burkina Faso
JQ045078	L3b1a+152	Burkina Faso
JQ045079	L1b1a3a	Burkina Faso
JQ045080	L2a1i	Burkina Faso
JQ045081	L4b1a	Burkina Faso
JQ045082	L2a1c5	Burkina Faso
JQ045083	L1b1a3	Burkina Faso
JQ045084	L3f1b4c	Burkina Faso
JQ045085	L3f1b1a	Burkina Faso
JQ045086	L1b1a	Burkina Faso

JQ045087	L2a1f	Burkina Faso	JQ703986	L3b3	Unknown
JQ045088	L2a1a2b	Burkina Faso	JQ704094	L2c2b1a	unknown
JQ045089	L3e1	Burkina Faso	JQ704266	L1b1a15a	Unknown
JQ045090	L2a1f	Burkina Faso	JQ704668	L2a1f	unknown
JQ045091	L3d6	Burkina Faso	JQ704670	L3f1b1a	Ethiopia
JQ045092	L3e2a2	Burkina Faso	JQ704683	L1b1a17	Unknown
JQ045093	L3f1b4b	Burkina Faso	JQ704728	L3e1f	Unknown
JQ045094	L1b1a18	Burkina Faso	JQ704740	L2c2a1	unknown
JQ045095	L2a1a2b	Burkina Faso	JQ704825	L1b1a7a	Spain
JQ045096	L3e1e	Burkina Faso	JQ704917	L3e2b1a1	Ghana
JQ045097	L2a1f2	Burkina Faso	JQ704919	L3b3	Unknown
JQ045098	L2a1c5	Burkina Faso	JQ704931	L3e3b1	Unknown
JQ045099	L1c3a1b	Burkina Faso	JQ704968	L1b1a8	Unknown
JQ045100	L1b1a10	Burkina Faso	JQ704978	L2c	unknown
JQ045101	L2a1a3c	Burkina Faso	JQ705732	L1b1a3	Unknown
JQ045102	L2a1i1	Burkina Faso	JQ705753	L3b1a	Unknown
JQ045103	L3e3b1	Burkina Faso	JQ705783	L3b1a1	Unknown
JQ045104	L2c3a	Burkina Faso	JQ705832	L1b1a10	Unknown
JQ045105	L2c1a	Burkina Faso	JQ705851	L3e3b2	Unknown
JQ045106	L2c1a	Burkina Faso	JQ705864	L1c2b1a1	Unknown
JQ045107	L1b1a4	Burkina Faso	JQ705902	L3e1a3a	USA
JQ045108	L2a1c2	Burkina Faso	JQ705912	L3d1d	South Africa
JQ702428	L0a1a+200	Unknown	JQ705931	L1b1a12b	Unknown
JQ702430	L2a1+143+16189	USA	JQ706014	L2a1a1	unknown
JQ702441	L1c3b1a	Unknown	JX021728	L2a1l3	Algeria
JQ702481	L3d1a2	Unknown	JX153016	L1b1a	Italy
JQ702503	L2a1c+16086	Mexico	JX266263	L1b1a12b	Poland
JQ702504	L4b2b1	Unknown	JX266264	L2a1l2a	Poland
JQ702533	L3e2a1b	Unknown	JX266265	L2a1l2a1	Poland
JQ702600	L1c3b1b	Unknown	JX303745	L0k2a1a	Zambia
JQ702617	L1c5	Unknown	JX303746	L1b1a10b	Zambia
JQ702626	L2b3a	unknown	JX303747	L3e4a	Zambia
JQ702659	L2a1c6	France	JX303748	L2d1a	Zambia
JQ702694	L2b1a3	unknown	JX303749	L2a1c5	Zambia
JQ702903	L1c2b1c	Unknown	JX303750	L2c2a1	Zambia
JQ702904	L2a1l2a1	unknown	JX303751	L5a2	Zambia
JQ702968	L2a1c+16129	unknown	JX303752	L2a1g	Zambia
JQ703036	L1b1a3b	Unknown	JX303753	L0k2a1a	Zambia
JQ703065	L2a1n	unknown	JX303754	L2a1b1a	Zambia
JQ703138	L3e2b1a	Unknown	JX303755	L1c2a1a	Zambia
JQ703481	L0a1a2	Unknown	JX303756	L1b1a	Zambia
JQ703618	L1b1a7a	Unknown	JX303757	L0a1a2	Zambia
JQ703621	L3f1b	Spain	JX303758	L3e1a2	Zambia
JQ703625	L3e3b	Unknown	JX303759	L3e1b2	Zambia
JQ703773	L1c4b	Unknown	JX303760	L2a1a2	Zambia
JQ703960	L2a1f	unknown	JX303761	L2a1b1a	Zambia

JX303762	L0a1b1	Zambia
JX303763	L0a2a2a	Zambia
JX303764	L1b1a10b	Zambia
JX303765	L0k2a1a	Zambia
JX303766	L0a1e	Zambia
JX303767	L3e1d1	Zambia
JX303806	L2a1d2	Zambia
JX303807	L2b2a	Zambia
JX303808	L3e1d1	Zambia
JX303809	L1b2a	Zambia
JX303810	L3d3a1	Zambia
JX303811	L1c2b1b1	Zambia
JX303812	L1b1a10b	Zambia
JX303813	L1b1a10b	Zambia
JX303814	L1b1a10b	Zambia
JX303815	L3d3a1b	Zambia
JX303816	L1c2b1b1	Zambia
JX303817	L0a1b1a	Zambia
JX303818	L0d1b2a2	Zambia
JX303819	L1b1a10b	Zambia
JX303820	L1c2b1b1	Zambia
JX303821	L3d1b3a	Zambia
JX303822	L3e2b1a2	Zambia
JX303823	L0a1b1a	Zambia
JX303824	L1c3a	Zambia
JX303825	L2a1d2	Zambia
JX303826	L0a2a2a	Zambia
JX303827	L3e1a3a	Zambia
JX303828	L1c2a1a	Zambia
JX303829	L2a5	Zambia
JX303830	L0a2a1b	Zambia
JX303831	L0a2a2a1	Zambia
JX303832	L2a1f	Zambia
JX303833	L1c2b1b1	Zambia
JX303834	L3d3a1b	Zambia
JX303835	L0a2a2a1	Zambia
JX303836	L3d3a1b	Zambia
JX303837	L3e1a3a	Zambia
JX303838	L2a1d2	Zambia
JX303839	L3d3a1b	Zambia
JX303840	L1b1a10b	Zambia
JX303841	L2b2a	Zambia
JX303842	L3f1b1a1	Zambia
JX303843	L3f2a1	Zambia
JX303882	L2b1a3	Zambia
JX303883	L1c2b1b1	Zambia
JX303884	L3e1f1a	Zambia
JX303885	L3e1e1	Zambia
JX303886	L3e1d1a	Zambia
JX303887	L3e1a3a	Zambia
JX303888	L3f1b1a1	Zambia
JX303889	L3e1d1a	Zambia
JX303890	L3b1a1a	Zambia
JX303891	L3e1d1	Zambia
JX303892	L1b1a3	Zambia
JX303893	L3e1a3a	Zambia
JX303894	L3f2a1	Zambia
JX303895	L0k2a1a	Zambia
JX303896	L1b2a	Zambia
JX303897	L0d2b1a1	Zambia
JX303898	L1b1a3	Zambia
JX303899	L0d2b1a1	Zambia
JX303900	L1c2b1b1	Zambia
JX303901	L1b1a3	Zambia
JX303902	L3e1d1	Zambia
JX303903	L0d2b1a1	Zambia
JX303904	L0a2a2a	Zambia
JX303905	L1b2a	Zambia
JX303906	L2a1g	Zambia
JX303907	L1c2a1a	Zambia
JX303908	L3e1a3a	Zambia
JX303909	L2a1i1	Zambia
JX303910	L1c2b1b1	Zambia
JX303911	L0a1a2	Zambia
JX303912	L1c2a1a	Zambia
JX303913	L1c2a1a	Zambia
JX524225	L2a1c1a2	Brazil
JX666328	L3b1a7	USA
KC152939	L1c2b1a1	Unknown
KC257334	L1c3b2	USA
KC257335	L1c3b2	USA
KC257336	L1c3b2	USA
KC345794	L0k1a2	Botswana
KC345795	L0k1a2	Botswana
KC345796	L0k1a2	Botswana
KC345797	L0k1a2	Botswana
KC345798	L0d1b1a1	Botswana
KC345799	L0d1b1a1	Botswana
KC345800	L0d1b1a1	Botswana
KC345801	L0d1b1a1	Botswana
KC345802	L0d1b1a1	Botswana
KC345803	L0k1a2	Botswana

KC345804	L0k1a2	Botswana	KC345888	L0d2b1b	Botswana
KC345805	L0k1a2	Botswana	KC345889	L0d2a1a	Botswana
KC345806	L0k1a2	Botswana	KC345890	L0d2a1a	Botswana
KC345807	L0d1b2a1	Botswana	KC345891	L0d2d	Botswana
KC345808	L0d1c2a	Botswana	KC345892	L0d2c1b	Botswana
KC345809	L0d1b2b2a	Botswana	KC345893	L0k1a1a	Botswana
KC345810	L0d1c1a1a1	Botswana	KC345894	L0k1a1	Botswana
KC345811	L0d1c1a1a	Botswana	KC345895	L0k1a1a	Botswana
KC345812	L0d1c1a1a	Botswana	KC345896	L0k1a1a	Botswana
KC345813	L0d1c1a1a1	Botswana	KC345897	L0k1a1d	Botswana
KC345814	L0d1c	Botswana	KC345898	L0k1a1a	Botswana
KC345815	L0d1c2a1	Botswana	KC345899	L0k1a1	Botswana
KC345816	L0d1c	Botswana	KC345900	L0d1b2b1a	Botswana
KC345817	L0d1c1a1a	Botswana	KC345901	L0d1b2b2c1	Botswana
KC345818	L0d1c	Botswana	KC345902	L0d1b2a2	Botswana
KC345819	L0d2a2	Botswana	KC345903	L0d1b2a2	Botswana
KC345820	L0d2a2	Botswana	KC345904	L0d1b2b1a	Botswana
KC345821	L0k1a2	Botswana	KC345905	L0d1a1b	Botswana
KC345822	L0d1c1a1a2	Botswana	KC345906	L0d1c1a1b	Botswana
KC345823	L0d1c1a1a1	Botswana	KC345907	L0d1c1a1b	Botswana
KC345824	L0d1c1a2	Botswana	KC345946	L0d1c1a1a	Botswana
KC345825	L0d1a1a1	Botswana	KC345947	L0d1c1a1a	Botswana
KC345826	L0d1c1a1a	Botswana	KC345948	L0d1b2a2	Botswana
KC345827	L0d1c2a	Botswana	KC345949	L0d1b2b1a	Botswana
KC345828	L0d1c2a	Botswana	KC345950	L0d1c1a2	Botswana
KC345829	L0d1c1a1a2	Botswana	KC345951	L0d1b2b2a	Botswana
KC345830	L0d1c1a1a	Botswana	KC345952	L0d2a1a1	Botswana
KC345831	L0d1a1a1	Botswana	KC345953	L0d2a2	Botswana
KC345870	L0d1b2b1a	Botswana	KC345954	L0d2b1b	Botswana
KC345871	L0d1b2b1a	Botswana	KC345955	L0d2a1a1a	Botswana
KC345872	L0d1b2a1	Botswana	KC345956	L0d2b1a1	Botswana
KC345873	L0d1b2b2c2	Botswana	KC345957	L0d2b1a1a	Botswana
KC345874	L0d1c1a1a	Botswana	KC345958	L0d2a1a	Botswana
KC345875	L0d1b2a1	Botswana	KC345959	L0d2b1b	Botswana
KC345876	L0d1b2a2	Botswana	KC345960	L0d2a1a1a	Botswana
KC345877	L0d1b2b1a	Botswana	KC345961	L0k1a2	Botswana
KC345878	L0d1b2b1a	Botswana	KC345962	L0k1a1	Botswana
KC345879	L0d1b2b1a	Botswana	KC345963	L0k1a1	Botswana
KC345880	L0d1b2b2c1	Botswana	KC345964	L0k1a1a	Botswana
KC345881	L0d1b2b1a	Botswana	KC345965	L0k1a1b	Botswana
KC345882	L0d1b2b2c1	Botswana	KC345966	L0k1a1d	Botswana
KC345883	L0d1b2b1a	Botswana	KC345967	L0d1c1a1a1	Botswana
KC345884	L0d1b2b1a	Botswana	KC345968	L0d1c1a1b	Botswana
KC345885	L0d1c1a1a	Botswana	KC345969	L0d1a1b1a	Botswana
KC345886	L0d2a1a	Botswana	KC345970	L0d1b2b2a	Botswana
KC345887	L0d2a1a	Botswana	KC345971	L0d1a1b1a	Botswana

KC345972	L0d1b2a1	Botswana	KC346056	L0d1b2b2a	Botswana
KC345973	L0d1a1b1a	Botswana	KC346057	L0d1b2b2a	Botswana
KC345974	L0d1a1b	Botswana	KC346058	L0d1c1a1a	Botswana
KC345975	L0d1c1a1a	Botswana	KC346059	L0d1b2a1	Botswana
KC345976	L0d1a1b1a	Botswana	KC346098	L0d1c1a1a	Namibia
KC345977	L0d1b2b2b	Botswana	KC346099	L0d1c1a1b	Namibia
KC345978	L0d1a1b1a	Botswana	KC346100	L0d1c1a1a	Namibia
KC345979	L0d1a1b1a	Botswana	KC346101	L0d1c1a1b	Namibia
KC345980	L0d1a1b1	Botswana	KC346102	L0d1a1a2	Namibia
KC345981	L0d1c1a2	Botswana	KC346103	L0d1c1a1a	Namibia
KC345982	L0k1a3	Botswana	KC346104	L0d2c1	Namibia
KC345983	L0d1b2b2a	Botswana	KC346105	L0d2c1	Namibia
KC346022	L0d2a1a1	Botswana	KC346106	L0d2a1a1	Namibia
KC346023	L0k1a1d	Botswana	KC346107	L0k1a1a	Namibia
KC346024	L0k1a1b	Botswana	KC346108	L0k1a1c	Namibia
KC346025	L0d1c2a	Botswana	KC346109	L0k1a1b	Namibia
KC346026	L0d1c1a1a2	Botswana	KC346110	L0k1a1a	Namibia
KC346027	L0d1b2a1	Botswana	KC346111	L0k1a1	Namibia
KC346028	L0d1b2a1	Botswana	KC346112	L0k1a1	Namibia
KC346029	L0d1b2a1	Botswana	KC346113	L0k1a1	Namibia
KC346030	L0d1c1a1a	Botswana	KC346114	L0k1a1a	Namibia
KC346031	L0d1c1a1a	Botswana	KC346115	L0k1a1	Namibia
KC346032	L0d1c2a	Botswana	KC346116	L0d1b1a1	Namibia
KC346033	L0d2a1a1	Botswana	KC346117	L0d1b1a1	Namibia
KC346034	L0k1a2	Botswana	KC346118	L0k1a2	Namibia
KC346035	L0k1a2	Botswana	KC346119	L0k1a2	Namibia
KC346036	L0d1b2a1	Botswana	KC346120	L0d1b1a1	Namibia
KC346037	L0d1c2a1	Botswana	KC346121	L0k1b	Namibia
KC346038	L0d1c1a2	Botswana	KC346122	L0d1b1a1	Namibia
KC346039	L0d1c1a1a	Botswana	KC346123	L0d1c1a1a	Namibia
KC346040	L0d1c1a1a	Botswana	KC346124	L0d1b2a1	Namibia
KC346041	L0d1c1a1a	Botswana	KC346125	L0d1b1b1	Namibia
KC346042	L0d1c1a1a1	Botswana	KC346126	L0d1b2b1a	Namibia
KC346043	L0d1b2a1	Botswana	KC346127	L0d2a1a1	Namibia
KC346044	L0d2a1c	Botswana	KC346128	L0d1b2b1b	Namibia
KC346045	L0d2a1c	Botswana	KC346129	L0d1a1c	Namibia
KC346046	L0k1a2a	Botswana	KC346130	L0d2b1b	Namibia
KC346047	L0d1c1a1a	Botswana	KC346131	L0d2c1b	Namibia
KC346048	L0k1a1d	Botswana	KC346132	L0d2c1b	Namibia
KC346049	L0k1a1d	Botswana	KC346133	L0k1a1c	Namibia
KC346050	L0d1b2b2a	Botswana	KC346134	L0d1c1a1b	Namibia
KC346051	L0d1b2b2a	Botswana	KC346135	L0d1b2b2c2	Namibia
KC346052	L0d2c2a1a	Botswana	KC346174	L0d2a1a	Namibia
KC346053	L0d2c2a1a	Botswana	KC346175	L0d1a1b1a	Namibia
KC346054	L0k1a1b	Botswana	KC346176	L0d1a1b1a	Namibia
KC346055	L0k1a1d	Botswana	KC346177	L0d1b2b2c1	Namibia

KC346178	L0d2a1a	Namibia
KC346179	L0k1a1	Namibia
KC346180	L0k1a1a	Namibia
KC346181	L0d1b2a2	Namibia
KC346182	L0d1b2a2	Namibia
KC346183	L0d1c2	Namibia
KC346184	L0d1b2a2	Namibia
KC346185	L0d1c1a1b	Namibia
KC346186	L0d1c3	Namibia
KC346187	L0d1b2b1a	Namibia
KC346188	L0d1c1a1b	Namibia
KC346189	L0d1a1b	Namibia
KC346190	L0d1c1a1a	Namibia
KC346191	L0d1c3	Namibia
KC346192	L0d1c1a1a	Namibia
KC346193	L0d2a1c	Namibia
KC346194	L0d2a1a	Namibia
KC346195	L0d2a1a	Namibia
KC346196	L0d2a1a1	Namibia
KC346197	L0d2a1c	Namibia
KC346198	L0d2a1c	Namibia
KC346199	L0k1a2a	Namibia
KC346200	L0k1a1	Namibia
KC346201	L0k1a1	Namibia
KC346202	L0k1a2a	Namibia
KC346203	L0k1a2a	Namibia
KC346204	L0k1a1a	Namibia
KC346205	L0k1a1d	Namibia
KC346206	L0d2c1a1	Namibia
KC346207	L0d2b1a	Namibia
KC346208	L0d1c1a1a	Namibia
KC346209	L0d1b2b2b1	Namibia
KC346210	L0d2c1a	Namibia
KC346211	L0d2a1a	Namibia
KC533453	L3e1b2	South Africa
KC533454	L2a1b1a	South Africa
KC533455	L2c2b1b	South Africa
KC533456	L1c2a3a	South Africa
KC533458	L3d1a1a1	South Africa
KC533460	L3e1a1a	South Africa
KC533461	L3e1a1a	South Africa
KC533462	L2a1a2	South Africa
KC533463	L3e3b1	South Africa
KC533466	L0a1b1a1	South Africa
KC533467	L1c2a3a	South Africa
KC533469	L3e1b2	South Africa
KC533470	L3e1f1a	South Africa
KC533472	L2a1g	South Africa
KC533474	L3b1a11	South Africa
KC533475	L0d3b	South Africa
KC533476	L0a1b1a1	South Africa
KC533477	L0d2a1b	South Africa
KC533478	L0d1a1b1b	South Africa
KC533479	L0a2a2a	South Africa
KC533480	L1c2a3a	South Africa
KC533481	L0d2a1	South Africa
KC533483	L3e1a1a	South Africa
KC533484	L3e3a	South Africa
KC533485	L2a1b1a	South Africa
KC533486	L0d2a1a	South Africa
KC533487	L0d1a1d	South Africa
KC533488	L3e1a3a	South Africa
KC533490	L0d1c1a	South Africa
KC533494	L0d2c1	South Africa
KC533495	L0a2a2a	South Africa
KC533496	L0a1b1a1	South Africa
KC533497	L0d1a1c	South Africa
KC533498	L0a1b1a1	South Africa
KC533500	L2a1f3	South Africa
KC533502	L0d2a1a	South Africa
KC533503	L2a1b1a	South Africa
KC533504	L2a1b1a	South Africa
KC622084	L3d3a1a	Namibia
KC622085	L3f1b4a	Namibia
KC622086	L3f1b4a	Namibia
KC622087	L3d3a1a	Namibia
KC622088	L3d3a1a	Namibia
KC622089	L3d3a1	Namibia
KC622090	L3d3a1	Namibia
KC622091	L3f1b4a	Namibia
KC622092	L3d3a1a	Namibia
KC622093	L3f1b4a	Namibia
KC622094	L3f1b4a	Namibia
KC622095	L3f1b4a	Namibia
KC622096	L3d3a1	Namibia
KC622097	L3d3a1a	Namibia
KC622098	L3e2b1	Namibia
KC622099	L3d3a1a	Namibia
KC622100	L0g	Namibia
KC622101	L3d3a1	Namibia
KC622102	L3d3a1a	Namibia
KC622103	L3f1b4a	Namibia

KC622104	L0a1b1a1	Namibia
KC622105	L2a1f	Namibia
KC622106	L0a1b1a1	Botswana
KC622107	L0a1b1a	Botswana
KC622108	L3d3a1	Namibia
KC622109	L5a2	Botswana
KC622110	L2a1d2	Botswana
KC622111	L5a2	Botswana
KC622112	L0a1b1a1	Botswana
KC622113	L3e1b2	Botswana
KC622114	L0a1b1a1	Botswana
KC622115	L0a1b1a1	Botswana
KC622116	L3e1a3a	Botswana
KC622117	L0a1b1a1	Botswana
KC622118	L2a1f3	Botswana
KC622119	L3e1b2	Botswana
KC622120	L3f1b4a	Namibia
KC622121	L0a1b1a1	Namibia
KC622160	L3f1b4a	Namibia
KC622161	L3d3a1	Namibia
KC622162	L1b1a	Namibia
KC622163	L2b1a3	Namibia
KC622164	L0a1b1a1	Namibia
KC622165	L3e1a2	Namibia
KC622166	L3e1a2	Namibia
KC622167	L3e1a2	Namibia
KC622168	L3e1a2	Namibia
KC622169	L2a1i1	Namibia
KC622170	L1c2a1a	Namibia
KC622171	L3e1e1	Namibia
KC622172	L3e2b+152	Namibia
KC622173	L1c2b1b1	Namibia
KC622174	L3d3a1a	Namibia
KC622175	L3d3a1	Namibia
KC622176	L0g	Namibia
KC622177	L3d3a1a	Namibia
KC622178	L1b1a	Namibia
KC622179	L4b2a2c	Botswana
KC622180	L1c3a1a	Namibia
KC622181	L3d3a1a	Namibia
KC622182	L3d3a1a	Namibia
KC622183	L3e1e1	Namibia
KC622184	L2a1a3c	Namibia
KC622185	L3d1a1a	Namibia
KC622186	L3d3a1a	Namibia
KC622187	L0a2a2a	Namibia
KC622188	L3d3a1a	Namibia
KC622189	L3d3a1a	Namibia
KC622190	L3d3a1a	Namibia
KC622191	L1c2a3	Namibia
KC622192	L3d3a1a	Namibia
KC622193	L3d3a1a	Namibia
KC622194	L1c2a3	Namibia
KC622195	L3d3a1a	Namibia
KC622196	L3e1a2	Namibia
KC622197	L3e1a2	Namibia
KC622236	L2a1c	Botswana
KC622237	L3e1e1	Botswana
KC622238	L2a1a	Botswana
KC622239	L3e1e1	Botswana
KC622240	L3e1e1	Botswana
KC622241	L3d3a1	Botswana
KC622242	L3f1b1a	Botswana
KC622243	L2a1d2	Botswana
KC622244	L2a1a	Botswana
KC622245	L2a1b1a	Botswana
KC622246	L2a1d2	Botswana
KC622247	L3d3a1	Botswana
KC622248	L2a1d2	Botswana
KC622249	L0a2a2a	Botswana
KC622250	L5b1	Botswana
KC622251	L5b1	Botswana
KC622252	L3b1a1	Botswana
KC622253	L3d3a1	Botswana
KC622254	L2a1d2	Botswana
KC622255	L5a2	Botswana
KC622256	L5a2	Botswana
KC622257	L3e1a2	Botswana
KC622258	L4b2a2c	Botswana
KC622259	L4b2a2c	Botswana
KC622260	L3e2b	Botswana
KC622261	L1b1a10b	Botswana
KC622262	L3d3a1	Botswana
KC622263	L3d1a1a1	Botswana
KC622264	L3d1a1a1	Botswana
KC622265	L2a1c	Botswana
KC622267	L2a1h	Botswana
KC622268	L3e1e1	Botswana
KC622269	L3f1b1a	Botswana
KC622270	L2a1d2	Botswana
KC622271	L1c2b1b	Botswana
KC622272	L0a1b1a1	Botswana

KC911354	L2a1f3	Iran
KC911360	L3f1b+16292	Iran
KF055328	L3e1a1a	USA
KF055329	L3f1b1a	USA
KF055330	L2c	USA
KF055331	L1c4b	USA
KF055332	L2a1c4a1	USA
KF055869	L2c	Spain
KF055870	L2c	Spain
KF161500	L3b1a1a	Denmark
KF162786	L4b	Denmark
KF179062	L1b1a8	USA
KF255394	L2a1l1a	Dominican Republic
KF358472	L3e5e	Cameroon
KF358473	L3e5c	Cameroon
KF358474	L3e5c	Cameroon
KF358475	L3e5a1a	Cameroon
KF358476	L3e5b	Niger
KF358477	L3e5a1a	Cameroon
KF358478	L3e5	Cameroon
KF358479	L3e5	Chad
KF358480	L3e5f	Chad
KF358481	L3e5f	Chad
KF358482	L3e5c	Nigeria
KF358483	L3e5b	Nigeria
KF358484	L3e5d	Nigeria
KF358485	L3e5	Cameroon
KF358486	L3e5b	Cameroon
KF358487	L3e5d	Cameroon
KF358488	L3e5b	Cameroon
KF358489	L3e5b	Cameroon
KF358490	L3e5e	Cameroon
KF358712	L1c3b1a	Puerto Rico
KF450887	L1b1a3a	Pakistan
KF450890	L2a1f3	Pakistan
KF450894	L3d1a1a	Pakistan
KF450895	L2a1g	Pakistan
KF450901	L0a1b1a1	Pakistan
KF450910	L2a1a2	Pakistan
KF450917	L1c2b2	Pakistan
KF451141	L2a1c3a	Israel
KF451144	L0a1b1a	Israel
KF451149	L2b1a2	Israel
KF451150	L1b1a2	Israel
KF451154	L0a1a1	Israel
KF451163	L0a1a	Israel

KF451169	L3h1a2a1	Israel
KF451202	L2a1c+16129	Israel
KF451203	L3f1b+16292	Israel
KF451221	L3e1b1	Israel
KF451224	L1b1a2	Israel
KF451250	L3e3a	Israel
KF451254	L2a1+143+16189	Israel
KF451258	L2a1+143+16189	Israel
KF451260	L3e1b1	Israel
KF451418	L3d1b	Senegal
KF451419	L1b1a4a	Senegal
KF451420	L1b1a14	Senegal
KF451421	L1b1a17	Senegal
KF451422	L2a1c3b1	Senegal
KF451423	L3d1d	Senegal
KF451424	L3e4a1	Senegal
KF451425	L1b1a	Senegal
KF451426	L3b1a	Senegal
KF451427	L1b1a17	Senegal
KF451428	L2a1c3b1	Senegal
KF451429	L3b1a	Senegal
KF451430	L2c3a	Senegal
KF451431	L2a1c1	Senegal
KF451432	L3e4a1	Senegal
KF451433	L2c5	Senegal
KF451434	L2a1c5	Nigeria
KF451435	L2a1c5	Nigeria
KF451436	L3f1b4c	Nigeria
KF451437	L3f1b4c	Nigeria
KF451438	L1b1a3	Nigeria
KF451439	L3f1b4c	Nigeria
KF451440	L3f1b1a	Nigeria
KF451545	L0d1c1a1a	Namibia
KF451589	L2a2b1a	Congo
KF451590	L1c1a2b	Central African Republic
KF451591	L1c1a1a1a	Central African Republic
KF451592	L1c1a2b	Central African Republic
KF451593	L1c1a2a2	Central African Republic
KF451594	L1c1a2b	Central African Republic
KF451595	L1c1a1a1a	Central African Republic
KF451596	L1c1a1a1a	Central African Republic
KF451597	L1c4b	Central African Republic

KF451598	L0a2a1	Central African Republic
KF451599	L1c1a2b	Central African Republic
KF451600	L1c4b	Central African Republic
KF451653	L2c3a	Senegal
KF451654	L2c1a	Senegal
KF451655	L2c1a	Senegal
KF451656	L1b1a4	Senegal
KF451712	L3e2b1	Algeria
KF451714	L2a1+143	Algeria
KF451723	L3e2a	Algeria
KF451729	L3b1a7	Algeria
KF451734	L3e2a1b1	Algeria
KF451735	L2a1c2	Senegal
KF451736	L2c3a	Senegal
KF451737	L2a1c3b1	Senegal
KF451738	L1b1a	Senegal
KF451850	L3b1a1a	Kenya
KF451851	L0a2a2a	Kenya
KF451852	L2a1f3	Kenya
KF451853	L1c2a1a	Kenya
KF451854	L3b1a1a	Kenya
KF451855	L1c2a1a	Kenya
KF451856	L3b1a1a	Kenya
KF451857	L5a1	Kenya
KF451858	L3h1a1	Kenya
KF451859	L3e2b+152	Kenya
KF451860	L0a1a1	Kenya
KF451861	L0a1a2	Kenya
KF672834	L0a2a1a2	São Tomé e Principe
KF672835	L0a2a2a	Somalia
KF672836	L0a1a1	Sudan
KF672837	L0a1a1	Sudan
KF952774	L2a1l1a2	USA
KJ185394	L0a2a2a	Zambia
KJ185395	L3f1b4c	Zambia
KJ185396	L0a2a1a	Zambia
KJ185397	L0a2a1b	Zambia
KJ185398	L0a1b1	Zambia
KJ185399	L0a2d	Zambia
KJ185400	L0f1	Zambia
KJ185401	L1b1a+189	Zambia
KJ185402	L1c3b1a	Zambia
KJ185403	L2a1d2	Zambia
KJ185404	L3b1b	Zambia
KJ185405	L3e1a1a	Zambia
KJ185406	L3f1b4c	Zambia
KJ185407	L4b2a	Zambia
KJ185408	L0f1	Zambia
KJ185409	L3d1a1a	Zambia
KJ185410	L3e1d1	Zambia
KJ185411	L3f1b1a1	Zambia
KJ185412	L1b1a15	Zambia
KJ185413	L1b1a3	Zambia
KJ185414	L3b1a	Zambia
KJ185415	L3b1a	Zambia
KJ185416	L3e2b+152	Zambia
KJ185417	L1b1a3	Zambia
KJ185418	L1c2b1b1	Zambia
KJ185419	L1c2b1b	Zambia
KJ185420	L1c2a1a	Zambia
KJ185421	L2d1a	Zambia
KJ185422	L3e2b3	Zambia
KJ185423	L0a1b1a1	Zambia
KJ185424	L0a2a1a	Zambia
KJ185425	L0a1b1a1	Zambia
KJ185426	L1c2b1b1	Zambia
KJ185465	L1c1	Zambia
KJ185466	L1c2a2	Zambia
KJ185467	L1c2b1b	Zambia
KJ185468	L2a1c5	Zambia
KJ185469	L3b1a1a	Zambia
KJ185470	L3b1a11	Zambia
KJ185471	L3e1e1	Zambia
KJ185472	L3e1a3a	Zambia
KJ185473	L3e1	Zambia
KJ185474	L3e2b1a2	Zambia
KJ185475	L3e1e1	Zambia
KJ185476	L0a2a1b	Angola
KJ185477	L0a2a2a	Angola
KJ185478	L0a2a2a	Angola
KJ185479	L0a2a2a	Angola
KJ185480	L0a2a1b	Angola
KJ185481	L1c1b	Angola
KJ185482	L1c2b2	Angola
KJ185483	L1c2b1b1	Angola
KJ185484	L1c2b1b	Angola
KJ185485	L1c3a1a	Angola
KJ185486	L2a1c+16129	Angola
KJ185487	L2a1c4a1	Angola
KJ185488	L2a1c4a1	Angola

KJ185489	L2b1a3	Angola
KJ185490	L3d1a1a	Angola
KJ185491	L3d3a1	Angola
KJ185492	L3e1e1	Angola
KJ185493	L3e2b	Angola
KJ185494	L0a2a1b	Angola
KJ185495	L0a2a1b	Angola
KJ185496	L0a2a1b	Angola
KJ185497	L0a1b2	Angola
KJ185498	L0a2a1b	Angola
KJ185499	L0a2a1b	Angola
KJ185500	L0a2a1b	Angola
KJ185501	L0a2a1b	Angola
KJ185502	L0a1b2	Angola
KJ185541	L0a2a2a1	Zambia
KJ185542	L0a1b1a1	Zambia
KJ185543	L0a2a1b	Zambia
KJ185544	L0a2a2a	Zambia
KJ185545	L0a1b1a1	Zambia
KJ185546	L0a2a1b	Zambia
KJ185547	L0a1b1a1	Zambia
KJ185548	L0a1b1a1	Zambia
KJ185549	L0a1b1a1	Zambia
KJ185550	L0a2a2a1	Zambia
KJ185551	L0a1b2a	Zambia
KJ185552	L0a1b1a1	Zambia
KJ185553	L0a2a2a1	Zambia
KJ185554	L0a1b1a1	Zambia
KJ185555	L0a1b1a1	Zambia
KJ185556	L0a2a2a	Zambia
KJ185557	L0a2a1b	Zambia
KJ185558	L1b2a	Zambia
KJ185559	L1b2a	Zambia
KJ185560	L1b2a	Zambia
KJ185561	L1b1a10b	Zambia
KJ185562	L1c1d	Zambia
KJ185563	L1c3a	Zambia
KJ185564	L1c2a1a	Zambia
KJ185565	L1c1b	Zambia
KJ185566	L1c2a1a	Zambia
KJ185567	L1c2b1b1	Zambia
KJ185568	L1c2b1b1	Zambia
KJ185569	L1c2b1b1	Zambia
KJ185570	L1c2a1a	Zambia
KJ185571	L1c2a1a	Zambia
KJ185572	L1c2b1b1	Zambia

KJ185573	L1c2a1a	Zambia
KJ185574	L1c1d	Zambia
KJ185575	L1c3a	Zambia
KJ185576	L1c2b1b1	Zambia
KJ185577	L1c3b1a	Zambia
KJ185578	L1c2b1b1	Zambia
KJ185617	L3e1d1a	Zambia
KJ185618	L3e1d1	Zambia
KJ185619	L3e3b	Zambia
KJ185620	L3e1a3a	Zambia
KJ185621	L3e1a3a	Zambia
KJ185622	L3e1d1a	Zambia
KJ185623	L3e1e1	Zambia
KJ185624	L3e2b	Zambia
KJ185625	L3e1d1a	Zambia
KJ185626	L3e1a3a	Zambia
KJ185627	L3e1d1	Zambia
KJ185628	L3e1a3a	Zambia
KJ185629	L3e1a3a	Zambia
KJ185630	L3e1e1	Zambia
KJ185631	L3e2b	Zambia
KJ185632	L3e2a1b1	Zambia
KJ185633	L3e1a3a	Zambia
KJ185634	L3e1d1	Zambia
KJ185635	L3e2b	Zambia
KJ185636	L3e3b	Zambia
KJ185637	L3e3b2	Zambia
KJ185638	L3e3b1	Zambia
KJ185639	L3e1a3a	Zambia
KJ185640	L3e3a	Zambia
KJ185641	L3e1d1a	Zambia
KJ185642	L3e1a3a	Zambia
KJ185643	L3e1a3a	Zambia
KJ185644	L3e1d1a	Zambia
KJ185645	L3e1a3a	Zambia
KJ185646	L3e2b	Zambia
KJ185647	L3f1b4a	Zambia
KJ185648	L5a2	Zambia
KJ185649	L5a2	Zambia
KJ185650	L1c3b1a	Zambia
KJ185651	L1c2a1a	Zambia
KJ185652	L3e1d1	Zambia
KJ185653	L0a2a1b	Zambia
KJ185654	L0a2a1b	Zambia
KJ185693	L2a1g	Zambia
KJ185694	L2b2a	Zambia

KJ185695	L2e1a	Zambia
KJ185696	L3b1a11	Zambia
KJ185697	L3d3a1	Zambia
KJ185698	L3e1a3a	Zambia
KJ185699	L3e3b	Zambia
KJ185700	L3e4a	Zambia
KJ185701	L3e1e1	Zambia
KJ185702	L3e1a1a	Zambia
KJ185703	L3e1a3a	Zambia
KJ185704	L3e1	Zambia
KJ185705	L3e1a3a	Zambia
KJ185706	L3e1d1	Zambia
KJ185707	L3e1b2	Zambia
KJ185708	L3e1a3a	Zambia
KJ185709	L3e3b	Zambia
KJ185710	L3e3b2	Zambia
KJ185711	L3e1a3a	Zambia
KJ185712	L3f2a1	Zambia
KJ185713	L3f1b1a1	Zambia
KJ185714	L5a2	Zambia
KJ185715	L5a2	Zambia
KJ185716	L5a2	Zambia
KJ185717	L0a2a1b	Zambia
KJ185718	L0a1b1a1	Zambia
KJ185719	L0a2a2a1	Zambia
KJ185720	L0a2a1b	Zambia
KJ185721	L0a2a2a	Zambia
KJ185722	L0a2a1a	Zambia
KJ185723	L1c2b2	Zambia
KJ185724	L1c3a	Zambia
KJ185725	L1c2b2	Zambia
KJ185726	L1c1b	Zambia
KJ185727	L1c3a1	Zambia
KJ185728	L2a1f	Zambia
KJ185729	L2a5	Zambia
KJ185730	L2a5	Zambia
KJ185769	L2a1a2	Angola
KJ185770	L2a1a3c	Angola
KJ185771	L2a1a3c	Angola
KJ185772	L2b1a3	Angola
KJ185773	L2c3	Angola
KJ185774	L2c2b1b	Angola
KJ185775	L2c2b1b	Angola
KJ185776	L3b1a1a	Angola
KJ185777	L3b1a1a	Angola
KJ185778	L3b1a10	Angola

KJ185779	L3d1a1a	Angola
KJ185780	L3d3a1	Angola
KJ185781	L3e2b	Angola
KJ185782	L3e1	Angola
KJ185783	L3e4a	Angola
KJ185784	L3e1e1	Angola
KJ185785	L3e3b1	Angola
KJ185786	L3e1a2	Angola
KJ185787	L3e2b1a2	Angola
KJ185788	L3e3b1	Angola
KJ185789	L3e1e1	Angola
KJ185790	L3e2b	Angola
KJ185791	L3e2b1a2	Angola
KJ185792	L3e1e1	Angola
KJ185793	L3e1a1a	Angola
KJ185794	L3e2b1a2	Angola
KJ185795	L3f1b4a	Angola
KJ185796	L3f1b4a	Angola
KJ185797	L3f1b4a	Angola
KJ185798	L3f1b4a	Angola
KJ185799	L3f1b1a	Angola
KJ185800	L3f1b4a	Angola
KJ185801	L3f1b4a	Angola
KJ185802	L0a2a2a	Angola
KJ185803	L0a2a2a1	Angola
KJ185804	L0a2a2a	Angola
KJ185805	L0a1b2a	Angola
KJ185806	L0a1a2	Angola
KJ185845	L3e2a1a	Angola
KJ185846	L3e3a	Angola
KJ185847	L3e2b	Angola
KJ185848	L3e1f	Angola
KJ185849	L3e2b+152	Angola
KJ185850	L3e1	Angola
KJ185851	L3e2b+152	Angola
KJ185852	L3e1d1	Angola
KJ185853	L3e1a1a	Angola
KJ185854	L3e1a1a	Angola
KJ185855	L3e1d1	Angola
KJ185856	L3e2b3	Zambia
KJ185857	L2b1a3	Zambia
KJ185858	L0a1b1a1	Zambia
KJ185859	L1c2b1b1	Zambia
KJ185860	L1c2a1a	Zambia
KJ185861	L3e2b1a2	Zambia
KJ185862	L0a2a2a	Zambia

KJ185863	L0a1a2	Zambia
KJ185864	L3e2b	Zambia
KJ185865	L0a2a1b	Zambia
KJ185866	L1b2a	Zambia
KJ185867	L1b2a	Zambia
KJ185868	L1c3a	Zambia
KJ185869	L2b1a3	Zambia
KJ185870	L3e2b+152	Zambia
KJ185871	L3f2a1	Zambia
KJ185872	L1b1a10b	Zambia
KJ185873	L1c3a	Zambia
KJ185874	L1c2b2	Zambia
KJ185875	L1c3a1b	Zambia
KJ185876	L1c3b1a	Zambia
KJ185877	L2c2a1	Zambia
KJ185878	L3d3a1	Zambia
KJ185879	L3e1a3a	Zambia
KJ185880	L3e1a3a	Zambia
KJ185881	L0a2a1a	Zambia
KJ185882	L0a2a1a	Zambia
KJ185921	L1c2a1a	Zambia
KJ185922	L1c2b1b	Zambia
KJ185923	L2a1b1a	Zambia
KJ185924	L3e1a3a	Zambia
KJ185925	L3e1e1	Zambia
KJ185926	L0a1b2a	Zambia
KJ185927	L1c2b1b1	Zambia
KJ185928	L1c2b1b1	Zambia
KJ185929	L1c2b1b1	Zambia
KJ185930	L1c2b1b1	Zambia
KJ185931	L2a1b1a	Zambia
KJ185932	L2a5	Zambia
KJ185933	L2a1f1	Zambia
KJ185934	L2a1a2	Zambia
KJ185935	L3d2b	Zambia
KJ185936	L3e1d1	Zambia
KJ185937	L3f2a1	Zambia
KJ185938	L1b1a10b	Zambia
KJ185939	L2a1d2	Zambia
KJ185940	L3e1a2	Zambia
KJ185941	L1c3b1a	Zambia
KJ185942	L2a5	Zambia
KJ185943	L3e2b1a2	Zambia
KJ185944	L3e3b2	Zambia
KJ185945	L5a2	Zambia
KJ185946	L0a2a2a1	Zambia
KJ185947	L0a2a2a1	Zambia
KJ185948	L1c2b1b1	Zambia
KJ185949	L1c2b	Zambia
KJ185950	L1c3a	Zambia
KJ185951	L1c3a1b	Zambia
KJ185952	L2a5	Zambia
KJ185953	L2a5	Zambia
KJ185954	L2a5	Zambia
KJ185955	L2a1d2	Zambia
KJ185956	L2b1a3	Zambia
KJ185957	L3e1a3a	Zambia
KJ185958	L3e1e1	Zambia
KJ185997	L2a1a2a1a	Zambia
KJ185998	L2a1d2	Zambia
KJ185999	L2a1c	Zambia
KJ186000	L3e1e1	Zambia
KJ186001	L0a1b1a1	Zambia
KJ186002	L2a1f1	Zambia
KJ186003	L2a1h	Zambia
KJ186004	L0a1a2	Zambia
KJ186005	L0a1a2	Zambia
KJ186006	L3e2b	Zambia
KJ186007	L3e2b1	Angola
KJ186008	L0a1b2	Angola
KJ186009	L0a1	Angola
KJ445738	L0d3	Pakistan
KJ445739	L0d2a1a	South Africa
KJ445740	L0d1b2a1	Namibia
KJ445741	L0d1b2a1	Namibia
KJ445742	L0d1b2b2c1	Namibia
KJ445743	L0d1b2b2b1	South Africa
KJ445744	L0d1c3	Namibia
KJ445745	L0d1c1a1a	Namibia
KJ445746	L0k1a1a	Namibia
KJ445747	L0a1b1a1	Pakistan
KJ445748	L0a1b1a	Israel
KJ445749	L0a1a1	Israel
KJ445750	L0a1a1	Kenya
KJ445751	L0a1a	Israel
KJ445752	L0a1a2	Kenya
KJ445753	L0a2b1	Congo
KJ445754	L0a2b	Congo
KJ445755	L0a2b	Congo
KJ445756	L0a2b	Congo
KJ445757	L0a2a1	Central African Republic

KJ445758	L0a2a1	Central African Republic
KJ445759	L0a2a1	Central African Republic
KJ445760	L0a2a1	Central African Republic
KJ445761	L0a2a2a	Kenya
KJ445762	L0a2a2a	Pakistan
KJ445801	L1c2b2	Pakistan
KJ445802	L1b1a3a	Pakistan
KJ445803	L1b1a17	Senegal
KJ445804	L1b1a	Senegal
KJ445805	L1b1a2	Israel
KJ445806	L1b1a2	Israel
KJ445807	L1b1a4a	Senegal
KJ445808	L1b1a4	Senegal
KJ445809	L1b1a	Nigeria
KJ445810	L1b1a17	Senegal
KJ445811	L1b1a18	Nigeria
KJ445812	L1b1a3	Nigeria
KJ445813	L1b1a14	Senegal
KJ445814	L1b1a	Senegal
KJ445815	L1b1a10	Nigeria
KJ446417	L4b2a2c	Namibia
KJ446418	L3h1a2a1	Israel
KJ446419	L3h1a1	Kenya
KJ446509	L3d3a1a	South Africa
KJ446510	L3d3a1a	South Africa
KJ446511	L3d3a1a	South Africa
KJ446512	L3d1d	Senegal
KJ446513	L3d6	Nigeria
KJ446514	L3f1b+16292	Israel
KJ446515	L3f1b1a	Nigeria
KJ446516	L3f1b4b	Nigeria
KJ446517	L3f1b4c	Nigeria
KJ446518	L3f1b4c	Nigeria
KJ446519	L3f1b4c	Nigeria
KJ446532	L3b1a7	Algeria
KJ446533	L3b1a1a	Kenya
KJ446534	L3b1a1a	Kenya
KJ446535	L3b1a1a	Kenya
KJ446536	L3b1a	Senegal
KJ446537	L3b1a	Senegal
KJ446540	L3e4a1	Senegal
KJ446541	L3e4a1	Senegal
KJ446542	L3e3a	Israel
KJ446768	L2a1c5	Nigeria
KJ446769	L2a1+143+16189	Pakistan
KJ446770	L2a1+143+16189	Israel
KJ446771	L2a1c3a	Israel
KJ446772	L2a1+143+16189	Israel
KJ446773	L2a1+143+16189	Israel
KJ446774	L2a1f	Nigeria
KJ446775	L2a1+143	Algeria
KJ446776	L2a1f3	Kenya
KJ446777	L2a1f3	Pakistan
KJ446778	L2a1f	Nigeria
KJ669103	L0k1a2a	Namibia
KJ669104	L0k1a2	Namibia
KJ669105	L0k1a2	Namibia
KJ669106	L0k1a1c	Namibia
KJ669107	L0k1a1a	Namibia
KJ669108	L0k1a1	Namibia
KJ669109	L0k1a1a	Namibia
KJ669110	L0k1a1c	Namibia
KJ669111	L0k1a1b	Angola
KJ669112	L0g	Namibia
KJ669113	L0a2a2a2	Zimbabwe
KJ669114	L0a1b1a1	South Africa
KJ669115	L0a1b2	Namibia
KJ669116	L0a1b1	Namibia
KJ669117	L0a1b1a	Namibia
KJ669118	L0a2a2a	Namibia
KJ669119	L0a2a2a	Namibia
KJ669120	L0a1b1a1a	Zimbabwe
KJ669121	L0a1b1a1	Namibia
KJ669122	L0a2a2a1	Namibia
KJ669123	L0a2a2a1	Namibia
KJ669124	L0d1c1a1a	Namibia
KJ669125	L0d1c3	Namibia
KJ669126	L0d1c3	Namibia
KJ669127	L0d1c1a1b	Namibia
KJ669128	L0d1c2a	Namibia
KJ669129	L0d1c1a1b	Namibia
KJ669168	L0d2d	Namibia
KJ669169	L0d2a1a3	South Africa
KJ669170	L0d2a1a	South Africa
KJ669171	L0d2a1a2	South Africa
KJ669172	L0d2a1a	Namibia
KJ669173	L0d2a1a	Namibia
KJ669174	L0d2a1a	South Africa
KJ669175	L0d2a1a2	Namibia
KJ669176	L0d2a1a	South Africa
KJ669177	L0d2a1a	South Africa

KJ669178	L0d2a1a	Namibia	KM101646	L3d1'2'3'4'5'6	USA
KJ669179	L0d2a1a	Namibia	KM101647	L3e2a1b	USA
KJ669180	L0d2a1b	South Africa	KM101648	L3e2a1b1	USA
KJ801474	L3b3	USA	KM101649	L1b1a4	USA
KJ801475	L3e1f1a	USA	KM101650	L1c2a1a	USA
KJ801477	L2a1d1	USA	KM101651	L2a1c	USA
KJ801478	L2a1d1	USA	KM101652	L3b1a4	USA
KJ801479	L2a1d1	USA	KM101653	L2a1c5	USA
KJ801480	L2a1d1	USA	KM101654	L3k1	USA
KJ801481	L2a1a	USA	KM101655	L2a1a1	USA
KJ801482	L2b2	USA	KM101656	L1b1a6	USA
KJ801483	L1c3a	USA	KM101657	L2a1c2a	USA
KJ949141	L2b2a	USA	KM101658	L2a1a	USA
KJ959229	L3f1b	Spain	KM101659	L3b1a6	USA
KJ959230	L3f1b	Spain	KM101660	L2c	USA
KM096762	L2a1k	Serbia	KM101661	L3b3	USA
KM101569	L1b1a3	USA	KM101662	L0a1b2	USA
KM101570	L1b1a3	USA	KM101663	L2d1a	USA
KM101571	L2a1n	USA	KM101664	L2a1f	USA
KM101572	L3d1b3a	USA	KM101665	L3e3a	USA
KM101573	L2a1f3	USA	KM101707	L3e2b	USA
KM101576	L3b1a10	USA	KM101708	L3e1a1a	USA
KM101577	L2c2b1b	USA	KM101709	L3e3b3	USA
KM101578	L1c1c	USA	KM101711	L2a1a2	USA
KM101579	L3e1e	USA	KM101712	L2b2a	USA
KM101580	L0a2a1b	USA	KM101713	L2a1a2a1a	USA
KM101581	L2b1b	USA	KM101714	L1b1a	USA
KM101582	L3b2	USA	KM101715	L3e2b	USA
KM101626	L2a1b	USA	KM101716	L1c2b1a	USA
KM101627	L2c	USA	KM101717	L3d1'2'3'4'5'6	USA
KM101628	L1c5	USA	KM101718	L2c	USA
KM101629	L3e1	USA	KM101719	L2a1c	USA
KM101630	L2a1f	USA	KM101720	L2c2a	USA
KM101631	L3b1a6	USA	KM101721	L2a1c3a1	USA
KM101632	L1c2b1c	USA	KM101722	L3d1a1a	USA
KM101634	L3f1b+16292+150	USA	KM101724	L2a1c2	USA
KM101635	L2a1b+143	USA	KM101726	L1c2a2	USA
KM101636	L2b2a	USA	KM101727	L2b2	USA
KM101637	L3d1b2	USA	KM101728	L2a1a1	USA
KM101638	L3f1b4	USA	KM101730	L3e3b3	USA
KM101639	L2a1i	USA	KM101731	L1b1a3a	USA
KM101640	L1b1a3a	USA	KM101732	L3d1d	USA
KM101642	L2a1a3	USA	KM101734	L3e2b+152	USA
KM101643	L2a1a2a1a	USA	KM101735	L2a1c4a1	USA
KM101644	L0a1a2	USA	KM101736	L1b1a4	USA
KM101645	L3f1b4c	USA	KM101866	L2a1c1	USA

KM101868	L2a1l1b	USA
KM102043	L3e4a	USA
KM102048	L1c5	USA
KM102058	L3e2a1b	USA
KM102059	L3e1d1	USA
KM102062	L1b2a	USA
KM102081	L0a2a1a1	USA
KM102086	L1c2b1b	USA
KM102090	L2a1f	USA
KM102093	L2a1l2	USA
KM102094	L3d2b	USA
KM102095	L3e2b	USA
KM986577	L3f2a1a	Yemen
KM986580	L0a2a2a	Yemen
KM986581	L3b1a11	Yemen
KM986584	L3d1a1a	Yemen
KM986588	L3d2a	Yemen
KM986589	L3h2	Yemen
KM986590	L3b1a1a	Yemen
KM986591	L3e1a2	Yemen
KM986592	L3e3a	Yemen
KM986593	L3d1a1a	Yemen
KM986595	L3e3a	Yemen
KM986596	L3b1a1a	Yemen
KM986598	L3b1a1a	Yemen
KM986599	L0a2a2a	Yemen
KM986600	L3d1a1a	Yemen
KM986601	L3d1a1a	Yemen
KM986604	L3h2	Yemen
KM986605	L3h2	Yemen
KM986606	L3h2	Yemen
KM986608	L4b2a1	Yemen
KM986609	L0a2a2a	Yemen
KM986614	L3i2	Yemen
KM986615	L3x1+16311	Yemen
KM986619	L3d1a1a1	Yemen
KM986620	L3x1a1	Yemen
KM986621	L3i2	Yemen
KM986624	L0a2a2a	Yemen
KM986627	L3i2	Yemen
KP229441	L3i1	Unknown
KP229442	L3i1	Unknown
KP229443	L3i1	Unknown
KP229444	L3i1	Unknown
KP229445	L3i1	Unknown
KP229446	L3i1	Unknown

KP229447	L3i1	Unknown
KP229448	L3i1	Unknown
KP229449	L3i1	Unknown
KP229450	L3i1a	Unknown
KP317076	L4a1a	Somalia
KP317077	L6a	Somalia
KP317078	L4b2a	Kenya
KP635236	L3d1b3	Unknown
KP635237	L1c3b1a	Bolivia
KP635238	L0a1b2	Bolivia
KP635239	L0a1b2	Bolivia
KP635240	L1c3b1a	Bolivia
KP635241	L3d1a1a	Unknown
KP635242	L1c3b1a	Bolivia
KP635243	L0a2a2a	Bolivia
KP875569	L3f1b1a	USA
KP899747	L2b2	USA
KP900753	L2a1+143	Brazil
KP900754	L2a1n	Brazil
KP900938	L1b1a15	Jamaica
KR135841	L2a1d1	Somalia
KR135842	L2a1b1a	Somalia
KR135843	L2a1+143+16189	Somalia
KR135844	L2a1+143+@16309	Somalia
KR135845	L2a1+143+16189	Somalia
KR135846	L2a1j	Somalia
KR135847	L2a1a	Somalia
KR135848	L2a1a2	Somalia
KR135849	L2a1+143	Somalia
KR135850	L2a1+143+@16309	Somalia
KR135851	L2a1+143+@16309	Somalia
KR135852	L2a1b1a	Somalia
KR135853	L2a1+143	Sudan
KR135854	L2a1+143	Sudan
KR135855	L2a1d1	Sudan
KR135856	L2a1a	Sudan
KR135857	L2a2a1	Sudan
KR135858	L2d+16129	Sudan
KR135859	L2b2	Sudan
KR135860	L2a2a	Sudan
KR135861	L2e1	Sudan
KR135862	L2e1	São Tomé e Principe
KT819240	L2b1a2	Morocco
KT819241	L3e5	Morocco
KT819242	L3e5a	Morocco
KT819244	L1b1a	Morocco

KT819245	L1b1a	Morocco
KT819246	L1b1a6	Morocco
KT819247	L2a1+143+16189	Morocco
KT819248	L2a1c	Morocco
KT819249	L3e5a	Morocco
KT819251	L1b1a8	Morocco
KT819252	L1b1a6	Morocco
KT819253	L2a1+143+16189 (	Morocco
KT819254	L2a1+143+16189	Morocco
KT819255	L2e	Morocco
KT819256	L3b1	Morocco
KT819257	L3b1a9	Morocco
KT819258	L3b1a3	Morocco
KT819259	L3b1a5	Morocco
KT819260	L3e2b+152	Morocco
KT819261	L3e2b1a2	Morocco
KT819262	L3e5a	Morocco
KT819263	L3e5a1	Morocco
KU867582	L3b1a9a	Unknown
KU867604	L3b1a9a	Unknown
KX055476	L3f1b4a	USA
KX079703	L0a1a2	USA
KX079704	L3b1a6	Unknown
KX083678	L2a1k	Bulgaria
KY295947	L2c1	USA
KY474046	L3b2a	USA
KY498629	L2e1a	USA
KY797199	L0a1b	Lebanon
KY797207	L3f1b+16292	Lebanon
LP6008115	L1b1a+189	Ireland
LP6008115	L1b1a8	Ireland
LP6008116	L1b1a8	Ireland
MF039862	L2a1a1	Tunisia
MF055825	L3d1a1a1	Madagascar
MF055830	L3d1a	Madagascar
MF055832	L3b1a1a	Madagascar
MF055837	L0a2a2a	Madagascar
MF055838	L2a1b1a	Madagascar
MF055845	L0a1b1a1	Madagascar
MF055846	L3e1a3a	Madagascar
MF055847	L3e3a	Madagascar
MF055849	L3e3a	Madagascar
MF055850	L0a1b1a1	Madagascar
MF055853	L2a1b1a	Madagascar
MF055856	L2b1a3	Madagascar
MF055857	L2a1b1a	Madagascar
MF055858	L0a2a2a	Madagascar
MF055859	L2a1b1a	Madagascar
MF055860	L0a1b1a1	Madagascar
MF055864	L3e3a	Madagascar
MF055867	L1c3a	Madagascar
MF055870	L2a1a2	Madagascar
MF055871	L2a1a2	Madagascar
MF055874	L2a1a2	Madagascar
MF055876	L2a1a2	Madagascar
MF055879	L0a2a2a	Madagascar
MF055882	L3d1a1a1	Madagascar
MF055886	L3e3b1	Madagascar
MF055889	L2a1a2	Madagascar
MF055890	L3e1d1	Madagascar
MF055891	L3f1b4a1	Madagascar
MF055892	L3d1a1a1	Madagascar
MF055895	L2a1b1a	Madagascar
MF055896	L1b2a	Madagascar
MF055898	L1b2a	Madagascar
MF055900	L3e1a3a	Madagascar
MF055910	L0a1b1a1	Madagascar
MF055911	L3d1a1a1	Madagascar
MF055912	L3b1a1a	Madagascar
MF055914	L2a1b1a	Madagascar
MF055917	L0a2a2a	Madagascar
MF056016	L2a1b1a	Madagascar
MF056018	L3b1a1a	Madagascar
MF056019	L2a1b1a	Madagascar
MF056021	L3e1a3a	Madagascar
MF056024	L0a1b1a1	Madagascar
MF056026	L3b1a1a	Madagascar
MF056027	L0a2a2a	Madagascar
MF056028	L3b1a1a	Madagascar
MF056040	L3e3b1	Madagascar
MF056041	L2a1b1a	Madagascar
MF056045	L2a1b1a	Madagascar
MF056047	L3e1a3a	Madagascar
MF056050	L3b1a1a	Madagascar
MF056051	L3b1a1a	Madagascar
MF056055	L2a1a2	Madagascar
MF056073	L3b1a1a	Madagascar
MF056075	L2a1b1a	Madagascar
MF056078	L3b1a1a	Madagascar
MF056087	L1c3a	Madagascar
MF056088	L3b1a1a	Madagascar
MF056089	L3d1a1a1	Madagascar

MF056091	L3b1a1a	Madagascar	MF056328	L0a1b1a1	Madagascar
MF056097	L3b1a1a	Madagascar	MF056330	L3e3b1	Madagascar
MF056099	L2a1b1a	Madagascar	MF056333	L1b1a+189	Madagascar
MF056100	L2a1b1a	Madagascar	MF056334	L2a1f	Madagascar
MF056104	L2a1c	Madagascar	MF056335	L1c3a1b	Madagascar
MF056110	L0a2a2a	Madagascar	MF056336	L2a1f	Madagascar
MF056118	L3e3b1	Madagascar	MF056337	L3b1a1a	Madagascar
MF056120	L3f1b4a	Madagascar	MF056338	L2a1b1a	Madagascar
MF056121	L3b1a1a	Madagascar	MF056341	L3e3a	Madagascar
MF056122	L1c3a	Madagascar	MF056419	L3d1a1a1	Madagascar
MF056123	L3b1a1a	Madagascar	MF056421	L3e3a	Madagascar
MF056125	L3e3b1	Madagascar	MF056422	L3e3a	Madagascar
MF056126	L3e1a1a	Madagascar	MF056427	L3d1a1a1	Madagascar
MF056128	L0a2a2a	Madagascar	MF056428	L2a1b1a	Madagascar
MF056129	L0a1b1a1	Madagascar	MF056430	L3b1a1a	Madagascar
MF056130	L3e1	Madagascar	MF056431	L3e3a	Madagascar
MF056131	L0a1a2	Madagascar	MF056432	L3b1a1a	Madagascar
MF056258	L3e1a3a	Madagascar	MF056434	L3d1a1a1	Madagascar
MF056260	L3b1a1a	Madagascar	MF056435	L0a2a1a	Madagascar
MF056261	L3b1a1a	Madagascar	MF056436	L3b1a3	Madagascar
MF056272	L3e1a1a	Madagascar	MF056437	L3d1a1a1	Madagascar
MF056274	L3e1a3a	Madagascar	MF056438	L3b1a1a	Madagascar
MF056276	L3d1a1a1	Madagascar	MF056439	L0a1+16293	Madagascar
MF056277	L2a1a2	Madagascar	MF056441	L3e1e1	Madagascar
MF056278	L3e1a3a	Madagascar	MF056444	L0a2	Madagascar
MF056280	L0a2a2a	Madagascar	MF056446	L3e3a	Madagascar
MF056281	L1c3c	Madagascar	MF056452	L0a2a1a	Madagascar
MF056285	L2a1a2	Madagascar	MF056453	L3e1a3a	Madagascar
MF056286	L3e1e1	Madagascar	MF056456	L2a1b1a	Madagascar
MF056287	L2a1a2	Madagascar	MF056461	L3e3a	Madagascar
MF056288	L3b1a1a	Madagascar	MF056463	L1c3c	Madagascar
MF056289	L2a1b1a	Madagascar	MF056464	L2a1b1a	Madagascar
MF056290	L3b1a1a	Madagascar	MF056465	L0a2a1a	Madagascar
MF056292	L1c3c	Madagascar	MF056466	L3d1a1a	Madagascar
MF056293	L2a1b1a	Madagascar	MF056470	L3d1a1a	Madagascar
MF056296	L3e3b1	Madagascar	MF056476	L2a1b1a	Madagascar
MF056297	L3b1a1a	Madagascar	MF056483	L3d1a1a1	Madagascar
MF056298	L3b1a1a	Madagascar	MF056484	L2a1b1a	Madagascar
MF056299	L3e3b1	Madagascar	MF056486	L3d1a1a1	Madagascar
MF056305	L2a1a2	Madagascar	MF056488	L1c3c	Madagascar
MF056313	L1c1d	Madagascar	MF056489	L3e2b	Madagascar
MF056317	L3e1a3a	Madagascar	MF056494	L3e1a3a	Madagascar
MF056318	L3b1a1a	Madagascar	MF056496	L3b1a1a	Madagascar
MF056321	L3e1a3a	Madagascar	MF056498	L3b1a1a	Madagascar
MF056324	L3b1a1a	Madagascar	MF056503	L3b1a1a	Madagascar
MF056325	L3b1a1a	Madagascar	MF056506	L3e1b2	Madagascar

MF056511	L3e1d1	Madagascar	MF056833	L3k1	Madagascar
MF056596	L2a1b1a	Madagascar	MF056835	L3b1a1a	Madagascar
MF056597	L3e1a3a	Madagascar	MF056837	L0a2a2a	Madagascar
MF056598	L2a1b1a	Madagascar	MF056838	L3e1a3a	Madagascar
MF056601	L3k1	Madagascar	MF056839	L3k1	Madagascar
MF056604	L2a1b1a	Madagascar	MF056840	L1b1a	Madagascar
MF056605	L3k1	Madagascar	MF056841	L3d1a1a	Madagascar
MF056606	L4b1a	Madagascar	MF056842	L3e3a	Madagascar
MF056608	L4b1a	Madagascar	MF056843	L3b1a8	Madagascar
MF056609	L3k1	Madagascar	MF056844	L3d1a	Madagascar
MF056610	L2a1b1a	Madagascar	MF056845	L3b1a1a	Madagascar
MF056611	L2a1b1a	Madagascar	MF056849	L3b1a1a	Madagascar
MF056615	L3e3b1	Madagascar	MF056850	L3b1a1a	Madagascar
MF056618	L3d1a1a	Madagascar	MF056862	L3b1a1a	Madagascar
MF056619	L2a1b1a	Madagascar	MF056874	L2a1f	Madagascar
MF056620	L3b1a1a	Madagascar	MF056877	L2a1b1a	Madagascar
MF056622	L2a1a	Madagascar	MF056878	L3f1b4a1	Madagascar
MF056624	L3e3b1	Madagascar	MF056879	L2a1b1a	Madagascar
MF056627	L0a2a2a	Madagascar	MF056881	L3b1a1a	Madagascar
MF056631	L2a1b1a	Madagascar	MF056883	L2a1f	Madagascar
MF056632	L2a1f	Madagascar	MF056886	L3e1b2	Madagascar
MF056633	L3e3b1	Madagascar	MF056888	L3b1a1a	Madagascar
MF056635	L3b1a1a	Madagascar	MF056893	L0a2a2a	Madagascar
MF056638	L3e3a	Madagascar	MF056896	L3e3a	Madagascar
MF056643	L3e3b1	Madagascar	MF056903	L3e1b2	Madagascar
MF056644	L2b1a3	Madagascar	MF056904	L2a1b1a	Madagascar
MF056647	L3b1a1a	Madagascar	MF056914	L2a1a2	Madagascar
MF056651	L3b1a1a	Madagascar	MF056915	L3e1a3a	Madagascar
MF056665	L2a1b1a	Madagascar	MF056916	L3b1a1a	Madagascar
MF056669	L3b1a8	Madagascar	MF056925	L3e1a3a	Madagascar
MF056672	L3e1a3a	Madagascar	MF056927	L3e1a3a	Madagascar
MF056673	L1c3a	Madagascar	MF057033	L3e3b	Madagascar
MF056678	L3b1a3	Madagascar	MF057037	L0a1b1a1	Madagascar
MF056680	L3b1a1a	Madagascar	MF057040	L3f1b4a1	Madagascar
MF056681	L3e2b+152	Madagascar	MF057042	L3f1b4a1	Madagascar
MF056686	L2a1b1a	Madagascar	MF057043	L2a1b1a	Madagascar
MF056688	L4b1a	Madagascar	MF057048	L2a1a2	Madagascar
MF056700	L3e2b+152	Madagascar	MF057051	L3b1a1a	Madagascar
MF056701	L3b1a1a	Madagascar	MF057052	L3b1a1a	Madagascar
MF056824	L2a1b1a	Madagascar	MF057053	L2a1b1a	Madagascar
MF056825	L3f1b4a1	Madagascar	MF057055	L2a1a2	Madagascar
MF056828	L3d1a1a	Madagascar	MF057056	L3k1	Madagascar
MF056829	L0a2a2a	Madagascar	MF057072	L2a1a2	Madagascar
MF056830	L0a2a2a	Madagascar	MF057073	L3e3b1	Madagascar
MF056831	L3b1a1a	Madagascar	MF057085	L0a2a1b	Madagascar
MF056832	L3e1a3a	Madagascar	MF057088	L3e1a1a	Madagascar

MF057093	L3e1	Madagascar
MF057096	L2a1b1a	Madagascar
MF057097	L3b1a1a	Madagascar
MF057098	L3b1a1a	Madagascar
MF057099	L3e3a	Madagascar
MF057103	L2a1b1a	Madagascar
MF057105	L3e1a1a	Madagascar
MF057106	L3b1a1a	Madagascar
MF057107	L3b1a1a	Madagascar
MF057109	L3d1a1a1	Madagascar
MF057111	L3b1a1a	Madagascar
MF057113	L3e3b1	Madagascar
MF057119	L3b1a8	Madagascar
MF057121	L0a2a2a	Madagascar
MF057122	L2a1b1a	Madagascar
MF057124	L0a2a2a	Madagascar
MF057125	L3b1a1a	Madagascar
MF057126	L2a1b1a	Madagascar
MF057127	L2a1b1a	Madagascar
MF057128	L2a1a2	Madagascar
MF057129	L2a1b1a	Madagascar
MF057130	L3d1a1a1	Madagascar
MF057132	L3e1a1a	Madagascar
MF057198	L2a1b1a	Madagascar
MF057199	L0a2a2a	Madagascar
MF057200	L3b1a1a	Madagascar
MF057203	L0a2a2a	Madagascar
MF057204	L3e1a1a	Madagascar
MF057205	L3e1a1a	Madagascar
MF057206	L3e1a1a	Madagascar
MF057209	L3d1a1a1	Madagascar
MF057210	L3d1a1a	Madagascar
MF057211	L3b1a1a	Madagascar
MF057213	L3b1a1a	Madagascar
MF057217	L2d1a	Madagascar
MF057218	L3e2b+152	Madagascar
MF057225	L2a1b1a	Madagascar
MF057228	L3e1d1	Madagascar
MF057232	L2a1b1a	Madagascar
MF057233	L1c1d	Madagascar
MF057235	L2a1b1a	Madagascar
MF057238	L2a1b1a	Madagascar
MF057239	L0a2a2a	Madagascar
MF057241	L0a1a2	Madagascar
MF057242	L2a1b1a	Madagascar
MF057245	L0a1b1a1	Madagascar
MF057246	L2a1a2	Madagascar
MF057247	L3e2b+152	Madagascar
MF057250	L3f1b4a1	Madagascar
MF057251	L3f	Madagascar
MF057252	L2a1a2	Madagascar
MF057253	L0a2a2a	Madagascar
MF057254	L3a+709	Madagascar
MF057255	L3a+709	Madagascar
MF057256	L3a+709	Madagascar
MF057258	L2a1b1a	Madagascar
MF057261	L3a+709	Madagascar
MF057266	L2a1a2	Madagascar
MF057268	L2a1b1a	Madagascar
MF057269	L3b1a3	Madagascar
MF057274	L2a5	Madagascar
MF057367	L3e3a	Madagascar
MF057369	L3b1a1a	Madagascar
MF057372	L0a2a2a	Madagascar
MF057373	L3e3b1	Madagascar
MF057376	L2a1b1a	Madagascar
MF057377	L2a1b1a	Madagascar
MF057379	L2b1a3	Madagascar
MF057380	L3e1a3a	Madagascar
MF057383	L0a2a2a	Madagascar
MF057385	L3e3a	Madagascar
MF057386	L3b1a1a	Madagascar
MF057387	L3e3a	Madagascar
MF057393	L3b1a1a	Madagascar
MF057396	L3a+709	Madagascar
MF057399	L3e3b1	Madagascar
MF057400	L3e1a3a	Madagascar
MF057403	L3e3b1	Madagascar
MF057404	L3b1a1a	Madagascar
MF057405	L3e3a	Madagascar
MF057406	L2a1b1a	Madagascar
MF057407	L3b1a1a	Madagascar
MF057412	L0a2a2a	Madagascar
MF057413	L3e1a3a	Madagascar
MF057418	L2a1b1a	Madagascar
MF057420	L0a2a2a	Madagascar
MF057422	L3k1	Madagascar
MF057424	L0a2a2a	Madagascar
MF057425	L3e1a3a	Madagascar
MF057428	L0a2a2a	Madagascar
MF057430	L2a1b1a	Madagascar
MF057431	L1c3a	Madagascar

MF057432	L0a2a2a	Madagascar	MF057724	L3e3a	Madagascar
MF057434	L3b1a1a	Madagascar	MF057725	L0a2a2a	Madagascar
MF057435	L3e1a3a	Madagascar	MF057726	L3e3a	Madagascar
MF057436	L3b1a1a	Madagascar	MF057728	L3e1a3a	Madagascar
MF057437	L3e3a	Madagascar	MF057729	L3e1a3a	Madagascar
MF057443	L3e1d1	Madagascar	MF057731	L3e1a3a	Madagascar
MF057444	L1c3b1a	Madagascar	MF057736	L3e3a	Madagascar
MF057523	L2a1b1a	Madagascar	MF057738	L0a2a2a	Madagascar
MF057526	L0a1b1a1	Madagascar	MF057740	L3b1a1a	Madagascar
MF057527	L1c3c	Madagascar	MF057741	L2a1b1a	Madagascar
MF057528	L3b1a1a	Madagascar	MF057745	L2a1b1a	Madagascar
MF057529	L2a1b1a	Madagascar	MF057747	L2a1f	Madagascar
MF057532	L2a1b1a	Madagascar	MF057750	L3b2	Madagascar
MF057535	L3e2b+152	Madagascar	MF057753	L3b2	Madagascar
MF057540	L3b1a1a	Madagascar	MF057754	L3e1a3a	Madagascar
MF057542	L3d1a1a	Madagascar	MF057762	L2a1a2	Madagascar
MF057544	L0a2a2a	Madagascar	MF057763	L3b1a1a	Madagascar
MF057545	L1c1d	Madagascar	MF057766	L3b1a1a	Madagascar
MF057550	L3f1b4a	Madagascar	MF057769	L3e3a	Madagascar
MF057553	L4b2a	Madagascar	MF057773	L3b1a1a	Madagascar
MF057554	L0a1b1a1	Madagascar	MF057783	L3b1a1a	Madagascar
MF057555	L3b1a1a	Madagascar	MF057785	L3b1a1a	Madagascar
MF057559	L3e2b+152	Madagascar	MF057786	L3b1a1a	Madagascar
MF057561	L3b1a1a	Madagascar	MF057787	L3d1a1a1	Madagascar
MF057563	L3e1a3a	Madagascar	MF057788	L3b1a1a	Madagascar
MF057566	L2a1b1a	Madagascar	MF057790	L2a1b1a	Madagascar
MF057572	L2a1b1a	Madagascar	MF057793	L3e1a3a	Madagascar
MF057573	L2a1b1a	Madagascar	MF057794	L2b1a3	Madagascar
MF057574	L0a2a2a	Madagascar	MF057796	L0a1e	Madagascar
MF057583	L0a1b1a1	Madagascar	MF057798	L1c3c	Madagascar
MF057586	L2a1b1a	Madagascar	MF057799	L3b1a1a	Madagascar
MF057587	L2a1b1a	Madagascar	MF057801	L3b1a1a	Madagascar
MF057590	L3b1a1a	Madagascar	MF057803	L3b1a1a	Madagascar
MF057591	L3d1a1a1	Madagascar	MF057808	L2a1b1a	Madagascar
MF057592	L3e3b1	Madagascar	MF057809	L3d1a1a1	Madagascar
MF057598	L2a1a2	Madagascar	MF057811	L0a1b1a1	Madagascar
MF057599	L3b1a1a	Madagascar	MF057813	L3e3b1	Madagascar
MF057600	L3d1a1a	Madagascar	MF057894	L2a1h	Madagascar
MF057605	L2a1a2a1a	Madagascar	MF057903	L1c3c	Madagascar
MF057607	L3b1a1a	Madagascar	MF057905	L4b1a	Madagascar
MF057617	L3d1a1a	Madagascar	MF057908	L3e1	Madagascar
MF057620	L3e3a	Madagascar	MF057910	L3d1a1a1	Madagascar
MF057622	L3e2b	Madagascar	MF057911	L3e1a3a	Madagascar
MF057623	L3f1b4a	Madagascar	MF057914	L3d1a1a1	Madagascar
MF057629	L3b1a1a	Madagascar	MF057915	L2a1b1a	Madagascar
MF057723	L3e1a3a	Madagascar	MF057916	L0a2a2a	Madagascar

MF057918	L3b1a1a	Madagascar	MF058103	L0f	Madagascar
MF057920	L3d1a1a1	Madagascar	MF058104	L3e1a3a	Madagascar
MF057921	L3d1a1a1	Madagascar	MF058105	L3e2b	Madagascar
MF057922	L3d1a1a1	Madagascar	MF058106	L2c2b1b	Madagascar
MF057927	L2a1f	Madagascar	MF058107	L3d1a1a	Madagascar
MF057928	L3b1a1a	Madagascar	MF058108	L1c2a1a	Madagascar
MF057929	L3e1d1	Madagascar	MF058110	L0a2a2a	Madagascar
MF057933	L0a2a1b	Madagascar	MF058112	L2c2b1b	Madagascar
MF057934	L3f1b4a1	Madagascar	MF058113	L0a2	Madagascar
MF057935	L3d1a1a	Madagascar	MF058115	L3e1a3a	Madagascar
MF057937	L3e3a	Madagascar	MF058116	L0a2	Madagascar
MF057938	L2a1b1a	Madagascar	MF058117	L3e3a	Madagascar
MF057940	L2a1b1a	Madagascar	MF058118	L2a1b1a	Madagascar
MF057943	L2a1a2	Madagascar	MF058119	L0a2	Madagascar
MF057946	L0a2a2a	Madagascar	MF058122	L0a2d	Madagascar
MF057947	L2a1a2	Madagascar	MF058123	L3d3a1	Madagascar
MF057948	L2a1a2	Madagascar	MF058124	L0a2a1b	Madagascar
MF057951	L0a2a2a	Madagascar	MF058125	L1c2b1b	Madagascar
MF057955	L0a2a2a	Madagascar	MF058126	L0a1'4	Madagascar
MF057956	L1c3a	Madagascar	MF058127	L2a1b1a	Madagascar
MF057959	L3e1	Madagascar	MF058137	L3d1a1a1	Madagascar
MF057960	L3e1a1a	Madagascar	MF058196	L0a1a2	Madagascar
MF057961	L3f1b4a1	Madagascar	MF058197	L0a1+16293	Madagascar
MF057967	L2a1a2	Madagascar	MF058198	L2b2a	Madagascar
MF057968	L2a1f	Madagascar	MF058199	L3e3a	Madagascar
MF057973	L3e1a3a	Madagascar	MF058200	L3e3a	Madagascar
MF057978	L2a1b1a	Madagascar	MF058203	L0a2a2a	Madagascar
MF057979	L3e1a3a	Madagascar	MF058204	L3b1a1a	Madagascar
MF057980	L2a1a	Madagascar	MF058205	L0a2a2a	Madagascar
MF058082	L3e3a	Madagascar	MF058206	L3e1a3a	Madagascar
MF058085	L2a1a	Madagascar	MF058207	L0a2a2a	Madagascar
MF058087	L3b1a1a	Madagascar	MF058209	L0a2a2a	Madagascar
MF058088	L2a1a	Madagascar	MF058210	L1b2a	Madagascar
MF058090	L3d1a1a1	Madagascar	MF058212	L3e1	Madagascar
MF058091	L1c3a	Madagascar	MF058216	L1c1	Madagascar
MF058092	L3b1a1a	Madagascar	MF058217	L0a1b1a1	Madagascar
MF058093	L3b1a1a	Madagascar	MF058219	L3e1a1a	Madagascar
MF058094	L1c3a1a	Madagascar	MF058222	L3e3b1	Madagascar
MF058095	L2a1a	Madagascar	MF058223	L2a1a2a1a	Madagascar
MF058096	L0a2a2a	Madagascar	MF058224	L0a2a2a	Madagascar
MF058097	L2a1b1a	Madagascar	MF058225	L0a2a1a2	Madagascar
MF058098	L1c2a1a	Madagascar	MF058227	L3b1a1a	Madagascar
MF058099	L2a1b1a	Madagascar	MF058233	L2a5	Madagascar
MF058100	L0a1b1a1	Madagascar	MF058234	L3b1a1a	Madagascar
MF058101	L0a1b1a1	Madagascar	MF058235	L4b1a	Madagascar
MF058102	L0a1b1a1	Madagascar	MF058237	L4b2a	Madagascar

MF058238	L3b1a1a	Madagascar
MF058241	L2a1b1a	Madagascar
MF058243	L3e1d1	Madagascar
MF058245	L4b1a	Madagascar
MF058246	L2a1b1a	Madagascar
MF058249	L3b1a+@16124	Madagascar
MF058251	L3d1a1a	Madagascar
MF058257	L3b1a1a	Madagascar
MF058259	L3b1a1a	Madagascar
MF058260	L3b1a1a	Madagascar
MF058261	L2a1b1a	Madagascar
MF058264	L3a1	Madagascar
MF058272	L3b1a1a	Madagascar
MF058385	L3e1a1a	Madagascar
MF058387	L0a1b1a1	Madagascar
MF058389	L2a1b1a	Madagascar
MF058392	L3e2b+152	Madagascar
MF058393	L0a2a2a	Madagascar
MF058394	L0a2a2a	Madagascar
MF058396	L2a1b1a	Madagascar
MF058397	L0a2a2a	Madagascar
MF058398	L3e3a	Madagascar
MF058399	L3d1a1a	Madagascar
MF058400	L3d1a1a	Madagascar
MF058401	L3b1a1a	Madagascar
MF058409	L0a2a2a	Madagascar
MF058411	L3f1b1a1	Madagascar
MF058413	L3e1a1a	Madagascar
MF058414	L1c2a1a	Madagascar
MF058417	L0a2a2a	Madagascar
MF058419	L0d1c	Madagascar
MF058421	L3e2b1a2	Madagascar
MF058422	L1c3b1a	Madagascar
MF058424	L3d1a1a1	Madagascar
MF058427	L0a1b1a1	Madagascar
MF058430	L2a1b1a	Madagascar
MF058431	L0a	Madagascar
MF058432	L3e3a	Madagascar
MF058433	L3e3a	Madagascar
MF058434	L3e3a	Madagascar
MF058435	L1c3c	Madagascar
MF058438	L3e3b	Madagascar
MF058441	L2a5	Madagascar
MF058445	L0a2a2a	Madagascar
MF058449	L2a1a2	Madagascar
MF058454	L0a1b1a1a	Madagascar

MF058458	L3e3b1	Madagascar
MF058460	L3b1a1a	Madagascar
MF058467	L3b1a1a	Madagascar
MF058469	L2a5	Madagascar
MF058471	L3b1a1a	Madagascar
MF058564	L2a1f	Madagascar
MF058565	L3b1a11	Madagascar
MF058566	L3e3a	Madagascar
MF058568	L3f2a1	Madagascar
MF058571	L3f1b1a	Madagascar
MF058578	L3b1a1a	Madagascar
MF058579	L0f	Madagascar
MF058581	L3b1a1a	Madagascar
MF058582	L3e1d1	Madagascar
MF058587	L3e3b1	Madagascar
MF058588	L0a2a2a	Madagascar
MF058590	L3e1a1a	Madagascar
MF058591	L2a1f	Madagascar
MF058593	L3b1a1a	Madagascar
MF058597	L0a2a2a	Madagascar
MF362754	L0a	Armenia
MF381287	L0a1b2	Angola
MF381288	L3f1b4a	Angola
MF381289	L3e1a2	Angola
MF381290	L0d1c3	Angola
MF381291	L0d1c3	Angola
MF381292	L0a1b1	Angola
MF381293	L0a1b1	Angola
MF381294	L3f1b4a	Angola
MF381295	L3f1b4a	Angola
MF381296	L0a1b1	Angola
MF381297	L1c1b	Angola
MF381298	L1c1b	Angola
MF381299	L0d1b1+@152	Angola
MF381300	L0a1b1	Angola
MF381301	L0a1b1	Angola
MF381302	L0a2a1b	Angola
MF381303	L0a1b2	Angola
MF381304	L3e1a2	Angola
MF381305	L0a1b1	Angola
MF381306	L3f1b4a	Angola
MF381307	L3f1b4a	Angola
MF381308	L1c1b	Angola
MF381347	L1c3a1b	Angola
MF381348	L3e1a3a	Angola
MF381349	L3f1b1a	Angola

MF381350	L2c2b1b	Angola	MF381434	L3f1b4a	Angola
MF381351	L3f1b4a	Angola	MF381435	L3e1a2	Angola
MF381352	L3f1b4a	Angola	MF381436	L3e1a2	Angola
MF381353	L1c2b1a'b	Angola	MF381437	L0a1b2	Angola
MF381354	L3e1a3a	Angola	MF381438	L3e2b	Angola
MF381355	L1c2b1a'b	Angola	MF381439	L3f1b4a	Angola
MF381356	L3e1a2	Angola	MF381440	L3e1a2	Angola
MF381357	L3e1e1	Angola	MF381441	L3f1b4a	Angola
MF381358	L3f1b4a	Angola	MF381442	L3e2b	Angola
MF381359	L3e1a2	Angola	MF381443	L3e1a2	Angola
MF381360	L1c1b	Angola	MF381444	L3d3a1a	Angola
MF381361	L3e1a2	Angola	MF381445	L3d3a1a	Angola
MF381362	L3f1b4a	Angola	MF381446	L0a1b2	Angola
MF381363	L3f1b4a	Angola	MF381447	L0a1b2	Angola
MF381364	L3d3a1a	Angola	MF381448	L3f1b4a	Angola
MF381365	L3f1b4a	Angola	MF381449	L1c1b	Angola
MF381366	L3f1b4a	Angola	MF381450	L0a1b1	Angola
MF381367	L3f1b4a	Angola	MF381451	L3e1a2	Angola
MF381368	L3f1b4a	Angola	MF381452	L3e1a2	Angola
MF381369	L0d1a1b1a	Angola	MF381453	L0a2a1b	Angola
MF381370	L0a1b1	Angola	MF381454	L0a1b1	Angola
MF381371	L3f1b4a	Angola	MF381455	L0a2a1b	Angola
MF381372	L0a1b1	Angola	MF381456	L0a1b1	Angola
MF381373	L0a1b1	Angola	MF381457	L3f1b4a	Angola
MF381374	L1c1b	Angola	MF381458	L0a2a1b	Angola
MF381375	L1c1b	Angola	MF381459	L0a2a1b	Angola
MF381376	L0a1b1	Angola	MF381460	L1c1b	Angola
MF381377	L0a2a1b	Angola	MF381499	L0d1b1b1	Angola
MF381378	L1c1b	Angola	MF381500	L0d1a1b1a	Angola
MF381379	L0a1b1	Angola	MF381501	L2c3	Angola
MF381380	L0a1b1	Angola	MF381502	L0d1a1b1a	Angola
MF381381	L1c1b	Angola	MF381503	L0d1a1b1a	Angola
MF381382	L0a1b1	Angola	MF381504	L0d1a1b1a	Angola
MF381383	L1c1b	Angola	MF381505	L1c1b	Angola
MF381384	L3f1b4a	Angola	MF381506	L0d1b1b1	Angola
MF381423	L3f1b4a	Angola	MF381507	L0a1b2	Angola
MF381424	L3f1b4a	Angola	MF381508	L0a1b2	Angola
MF381425	L1c1b	Angola	MF381509	L0d1b1b1	Angola
MF381426	L3f1b4a	Angola	MF381510	L1c2a1a	Angola
MF381427	L3f1b4a	Angola	MF381511	L0d1a1b1a	Angola
MF381428	L1c1b	Angola	MF381512	L3f1b4a	Angola
MF381429	L0a1b1	Angola	MF381513	L0d1b1b1	Angola
MF381430	L3f1b4a	Angola	MF381514	L2c3	Angola
MF381431	L3f1b4a	Angola	MF381515	L3f1b4a	Angola
MF381432	L3f1b4a	Angola	MF381516	L2c3	Angola
MF381433	L1c1b	Angola	MF381517	L2c3	Angola

MF381518	L0d1b1b1	Angola	MF621083	L3e2a1	Kenya
MF381519	L0d1a1b1a	Angola	MF621084	L3e2b+152	Mozambique
MF381520	L3f1b4a	Angola	MF621085	L3e3b	Mali
MF381521	L3d3a1	Angola	MF621086	L3e4a1	Mauritania
MF381522	L2c2b1b	Angola	MF621087	L3e5	Mali
MF381523	L2c2b1b	Angola	MF621088	L3e5a	Saudi Arabia
MF381524	L0d1b1b1	Angola	MF621089	L3e5	Spain
MF381525	L3f1b4a	Angola	MF621090	L3i1a	Saudi Arabia
MF381526	L1c3b1a	Angola	MF621091	L3i2	Sudan
MF381527	L3f1b4a	Angola	MF621092	L3x1a1	Saudi Arabia
MF381528	L3f1b4a	Angola	MF621093	L3x1a2	Kenya
MF381529	L0d1b1b1	Angola	MF695865	L0a1b1a1a	Kenya
MF381530	L0d1a1b1a	Angola	MF695866	L0f	Kenya
MF381531	L0d1b1b1	Angola	MF695867	L0a2a2a	Kenya
MF381532	L0d1b1b1	Angola	MF695868	L3b1a11	Kenya
MF381533	L3e2b1a2	Angola	MF695869	L0a1d	Kenya
MF381534	L0d1a1b1a	Angola	MF695870	L3b1a1a	Kenya
MF381535	L3e1a1a	Angola	MF695871	L0a2a1b	Kenya
MF381536	L3e1a2	Angola	MF695872	L1c1	Kenya
MF381575	L0d2a1a	Angola	MF695874	L1c2a3	Kenya
MF381576	L2e1	Angola	MF695875	L0a2	Kenya
MF381577	L3d3a1a	Angola	MF695876	L0a2a2a	Kenya
MF381578	L3f1b4a	Angola	MF695877	L0a1'4	Kenya
MF381579	L3e1a2	Angola	MF695878	L0a	Kenya
MF381581	L0d1a1b1a	Angola	MF695879	L2a1a2	Kenya
MF621062	L1c2b1a'b	Saudi Arabia	MF695880	L0a2a2a	Kenya
MF621063	L3a1a	Kenya	MF695881	L0f	Kenya
MF621064	L3b1a1a	Kenya	MF695882	L2a1a2	Kenya
MF621065	L3b1a+@16124	Sudan	MF695883	L0f1	Kenya
MF621066	L3b1a9	Senegal	MF695885	L3b1a1a	Kenya
MF621067	L3b1a+152	Mauritania	MF695886	L3e3a	Kenya
MF621068	L3b1a2	Sudan	MF695887	L3e3a	Kenya
MF621069	L3b2b	Mali	MF695888	L0a2a2a	Kenya
MF621070	L3f1a1	Sudan	MF695889	L3e3a	Kenya
MF621071	L3f1b+16292	Senegal	MF695890	L3d1a1a	Kenya
MF621072	L3f1b+16292	Saudi Arabia	MF695891	L0f1	Kenya
MF621073	L3f1b	Spain	MF695892	L2d1a	Kenya
MF621074	L3f2a1a	Sudan	MF695893	L0a1d	Kenya
MF621075	L3f2a1	Sudan	MF695894	L0f2a1	Kenya
MF621076	L3d1b1b	Sudan	MF695895	L0a2	Kenya
MF621077	L3d1b1b	Sudan	MF695896	L1b1a	Kenya
MF621078	L3d1b3a	Spain	MF695897	L0a2a2a	Kenya
MF621079	L3d1b3	Sudan	MF695898	L0f2a	Kenya
MF621080	L3d1c1	Ghana	MF695899	L0a	Kenya
MF621081	L3d3a1	Mozambique	MF695900	L2a1h	Kenya
MF621082	L3e1a3b	Ghana	MF695901	L4b2a2	Kenya

MF695902	L3e1e	Kenya
MF695903	L4b2a2	Kenya
MF695904	L3d1a1a1	Kenya
MF695944	L1c2a1	Kenya
MF695945	L1c2a1	Kenya
MF695946	L3e3a	Kenya
MF695947	L3x1a2	Kenya
MF695948	L1c2a1	Kenya
MF695949	L3d1d	Kenya
MF695950	L3h1a2a1	Kenya
MF695951	L4b2a2	Kenya
MF695952	L3h2	Kenya
MF695953	L1c2a1	Kenya
MF695954	L2a1f	Kenya
MF695955	L2a1f	Kenya
MF695956	L1c2a1	Kenya
MF695957	L1b1a	Kenya
MF695958	L1b2a	Kenya
MF695959	L4b2a2	Kenya
MF695960	L1b1a3	Kenya
MF695961	L3x1a2	Kenya
MF695962	L3d1a1a	Kenya
MF695963	L1b1a3	Kenya
MF695964	L2a1f1	Kenya
MF695965	L0a3	Kenya
MF695966	L1c2a1	Kenya
MF695967	L3a1	Kenya
MF695968	L3d1a1a	Kenya
MF695969	L1c2a1	Kenya
MF695970	L1c2a1	Kenya
MF695972	L3d1a1a	Kenya
MF695973	L2a1f	Kenya
MF695974	L0a2a2a	Kenya
MF695975	L3e3a	Kenya
MF695976	L4b2a2	Kenya
MF695977	L3e5	Kenya
MF695978	L0a2a2a	Kenya
MF695979	L2a1f	Kenya
MF695980	L3e3a	Kenya
MF695981	L2a1f	Kenya
MF695982	L3e2b	Kenya
MF696023	L0f	Kenya
MF696024	L2a1h	Kenya
MF696025	L2a1f	Kenya
MF696026	L3a2	Kenya
MF696027	L3e1e	Kenya

MF696028	L3d1a1a	Kenya
MF696029	L1c1	Kenya
MF696030	L2a1	Kenya
MF696031	L0a2a2a	Kenya
MF696032	L3x1a2	Kenya
MF696033	L0a1a1	Kenya
MF696034	L2a1a	Kenya
MF696035	L3e1e1	Kenya
MF696036	L3e3a	Kenya
MF696037	L0a2a2a	Kenya
MF696038	L3e3a	Kenya
MF696039	L0a1'4	Kenya
MF696040	L0f	Kenya
MF696041	L5a1	Kenya
MF696042	L2a5	Kenya
MF696044	L2a1c4a1	Kenya
MF696045	L2a1b1a	Kenya
MF696046	L3e1a2	Kenya
MF696047	L1c2a1a	Kenya
MF696048	L0a2a2a	Kenya
MF696049	L3x1a2	Kenya
MF696050	L3e3a	Kenya
MF696051	L0f2a	Kenya
MF696052	L0a2a2a	Kenya
MF696053	L3f1b4a1	Kenya
MF696054	L0d1c	Kenya
MF696055	L0a2a2a	Kenya
MF696056	L1c2a1a	Kenya
MF696057	L0a2a2a	Kenya
MF696058	L3d1a1a1	Kenya
MF696059	L0f	Kenya
MF696060	L3h1a2a1	Kenya
MF696061	L1b1a	Kenya
MF696103	L1c3a	Comoros
MF696104	L0a2	Comoros
MF696105	L0a2a1a	Comoros
MF696106	L2c2b1b	Comoros
MF696107	L2a5	Comoros
MF696108	L3e3a	Comoros
MF696109	L0a1b1a1	Comoros
MF696110	L3b1a1a	Comoros
MF696111	L3b1a1a	Comoros
MF696112	L1c3a	Comoros
MF696113	L2a1b1a	Comoros
MF696114	L0a2a2a	Comoros
MF696115	L0a2a2a	Comoros

MF696116	L2a1b1a	Comoros	MH981653	L0d2a1a	South Africa
MF696117	L3e3a	Comoros	MH981654	L1c2a3a	South Africa
MF696118	L3f2a1	Comoros	MH981655	L3e2b	South Africa
MF696119	L0a2a2a	Comoros	MH981657	L2a1b1a	South Africa
MF696120	L0a2a2a	Comoros	MH981658	L0d1a1d	South Africa
MF696121	L3e3a	Comoros	MH981659	L1c2a3a	South Africa
MF696122	L0a2a2a	Comoros	MH981660	L0a2a2a	South Africa
MF696123	L0g	Comoros	MH981661	L0a2a2a	South Africa
MF696124	L3e3b2	Comoros	MH981662	L3d1a1a1	South Africa
MF696125	L0k2b	Comoros	MH981663	L0a2a2a	South Africa
MF696126	L2a1b1a	Comoros	MH981664	L0a2a2a	South Africa
MF696127	L1c3c	Comoros	MH981665	L2a1b1a	South Africa
MF696128	L2a1g	Comoros	MH981666	L0d2b2	South Africa
MF696129	L2a1b1a	Comoros	MH981667	L3d1a1a1	South Africa
MF696130	L0a2a2a	Comoros	MH981668	L2a1b1a	South Africa
MF696131	L2a1f3	Comoros	MH981669	L0d2a1a	South Africa
MF696132	L2a1f	Comoros	MH981670	L0d1b2b2b	South Africa
MF696133	L3e1d1	Comoros	NA18499	L3e2a1b1	Nigeria
MF696134	L3f1b1a1	Comoros	NA18501	L1b1a18	Nigeria
MF696135	L3f1b1a1	Comoros	NA18502	L3e2b1a2	Nigeria
MF696136	L3f1b1a1	Comoros	NA18504	L3e2b	Nigeria
MF696137	L3e1d1	Comoros	NA18505	L2a1f2	Nigeria
MF696138	L3d1a1a1	Comoros	NA18507	L1b1a3	Nigeria
MF997504	L2a1a1	USA	NA18508	L3b1a7a	Nigeria
MF997533	L3f1b+16292	Unknown	NA18510	L0a1a3	USA
MH981628	L2a1b1a	South Africa	NA18511	L2a1f	Nigeria
MH981629	L4b2b1	South Africa	NA18516	L2b1a3	Nigeria
MH981630	L0d1b2b1b	South Africa	NA18517	L2c2b1a	Nigeria
MH981631	L0a2a1a2	South Africa	NA18520	L3f1b4b	Nigeria
MH981632	L0d1b2b1b	South Africa	NA18522	L2b1a3	Nigeria
MH981633	L0a1b1a1	South Africa	NA18523	L2a1f	Nigeria
MH981634	L0a2a2a	South Africa	NA18853	L3e2b	Nigeria
MH981635	L3e2b1a2	South Africa	NA18856	L2a1b3	Nigeria
MH981636	L2a1b1a	South Africa	NA18858	L3b3	Nigeria
MH981637	L0a2a2a	South Africa	NA18861	L0a1a2	Nigeria
MH981638	L0d2a1	South Africa	NA18864	L3d6	Nigeria
MH981639	L1c3a	South Africa	NA18865	L3b1a5a	Nigeria
MH981640	L2a1a2a1a	South Africa	NA18867	L3d1a1b	Nigeria
MH981641	L2a1a2a1a	South Africa	NA18868	L2a1c1a1	Nigeria
MH981642	L2a1b1a	South Africa	NA18870	L2b3a	Nigeria
MH981644	L0d2c2	South Africa	NA18871	L3b1a1	Nigeria
MH981645	L2a1b1a	South Africa	NA18873	L3e3b	Nigeria
MH981646	L3f1b4a1	South Africa	NA18874	L2a1a1	Nigeria
MH981647	L3f1b4a1	South Africa	NA18876	L0a1a2	Nigeria
MH981648	L0d1a1a	South Africa	NA18877	L0a1a2	Nigeria
MH981651	L0d1b2b	South Africa	NA18878	L2a1i1	Nigeria

NA18879	L4b2b	Nigeria
NA18881	L3f1b1a	Nigeria
NA18907	L3b1a5a	Nigeria
NA18908	L2a1c5	Nigeria
NA18909	L3e4a	Nigeria
NA18910	L2a1c2a	Nigeria
NA18912	L2a1c4a1	Nigeria
NA18915	L1c3b1b	Nigeria
NA18916	L3b2b	Nigeria
NA19114	L3e1a3b	Nigeria
NA19116	L2a1e1	Nigeria
NA19117	L2a1a2a1a	Nigeria
NA19118	L3e2a1b	Nigeria
NA19119	L3b1a7a	Nigeria
NA19121	L3d5a	Nigeria
NA19122	L2c1a	Nigeria
NA19124	L3b1a4	Nigeria
NA19125	L2a1f	Nigeria
NA19129	L2a1b1	Nigeria
NA19130	L2a1c3b2	Nigeria
NA19131	L1b1a	Nigeria
NA19133	L1b1a3	Nigeria
NA19135	L1b2a	Nigeria
NA19137	L0a1a2	Nigeria
NA19138	L3e2b3	Nigeria
NA19141	L3e1	Nigeria
NA19143	L3b1a7a	Nigeria
NA19144	L3d5a	Nigeria
NA19146	L2e1a	Nigeria
NA19147	L3e2b6	Nigeria
NA19149	L2a1c5	Nigeria
NA19150	L3e2b8	Nigeria
NA19153	L3d2b	Nigeria
NA19156	L0a1a+200	Nigeria
NA19157	L2a1f	Nigeria
NA19159	L3f1b4c	Nigeria
NA19160	L3e2b2	Nigeria
NA19162	L3e2a2	Nigeria
NA19163	L1c3b2	Nigeria
NA19166	L3e1b1	Nigeria
NA19168	L3e1	Nigeria
NA19171	L3e3b	Nigeria
NA19175	L3b1a8	Nigeria
NA19181	L3b1a8	Nigeria
NA19182	L3f1b1a	Nigeria
NA19184	L3e1b1	Nigeria
NA19185	L2b1a3	Nigeria
NA19309	L3e2b	Kenya
NA19310	L3b1a1a	Kenya
NA19311	L0a1a+200	Kenya
NA19312	L0a2a2a	Kenya
NA19315	L3e3b1	Kenya
NA19316	L3d1a	Kenya
NA19317	L5a1c	Kenya
NA19318	L5b2	Kenya
NA19319	L3e3b2	Kenya
NA19320	L3b1a1a	Kenya
NA19321	L3b1a1a	Kenya
NA19323	L1c2a1a	Kenya
NA19324	L3b1a1a	Kenya
NA19327	L0f	Kenya
NA19328	L0a2a2a	Kenya
NA19331	L3b1a1a	Kenya
NA19332	L5b2	Kenya
NA19334	L3b1a1a	Kenya
NA19338	L3b1a1a	Kenya
NA19346	L3b1a1a	Kenya
NA19347	L3b1a1a	Kenya
NA19350	L0a1a+200	Kenya
NA19351	L2a1a2	Kenya
NA19352	L3b1a1a	Kenya
NA19355	L3b1a1a	Kenya
NA19359	L4b2a2	Kenya
NA19360	L3b1a1a	Kenya
NA19371	L3e1a2	Kenya
NA19372	L3b1a1a	Kenya
NA19373	L5b1	Kenya
NA19374	L5b1	Kenya
NA19375	L3b1a1a	Kenya
NA19376	L2a4b	Kenya
NA19377	L3h1a2a1	Kenya
NA19378	L1c2b1a	Kenya
NA19379	L0a1a+200	Kenya
NA19380	L3b1a1a	Kenya
NA19381	L2a1q	Kenya
NA19455	L5b2	Kenya
NA19456	L3b1a1a	Kenya
NA19457	L3h1a1	Kenya
NA19461	L5b2	Kenya
NA19462	L1b1a15	Kenya
NA19463	L4b2a2b	Kenya
NA19466	L0a1b1a1a	Kenya

NA19467	L0a1c1	Kenya
NA19468	L3b1a1a	Kenya
NA19469	L2a2b1	Kenya
NA19470	L2a2b1	Kenya
NA19471	L1b1a3	Kenya
NA19472	L3b1a1a	Kenya
NA19473	L3b1a1a	Kenya
NA19474	L0b	Kenya
NA19475	L3b1a1a	Kenya
NA19625	L2a1b+143	USA
NA19700	L3e2b+152	USA
NA19703	L0a1b1a	USA
NA19704	L3e2a1a	USA
NA19707	L3e2a	USA
NA19711	L3e3b	USA
NA19712	L1c2b1c	USA
NA19713	L0a2a2a1	USA
NA19818	L3e1a3a	USA
NA19819	L3e2b+152	USA
NA19834	L1b1a	USA
NA19835	L2c5	USA
NA19900	L3e3b	USA
NA19901	L3f1b1a	USA
NA19904	L0a2a2a	USA
NA19908	L2a1c	USA
NA19909	L1b2	USA
NA19913	L3b1a+@16124	USA
NA19914	L1c3a1b	USA
NA19916	L1b1a9	USA
NA19917	L1c1b	USA
NA19920	L3h1b2	USA
NA20363	L1c3a	USA
NA20412	L1b1a	USA
NA20414	L2a1b+143	USA
NA20753	L1b1a5	Italy
NA20903	L2a1d	USA
NA21118	L3i1b	USA
NA21142	L3i1b	USA
