# Supplementary material for: Phylogeography of Sub-Saharan Mitochondrial Lineages Outside Africa Highlights the Roles of the Holocene Climate Changes and the Atlantic Slave Trade
Source: Int J Mol Sci. 2022 Aug 16;23(16):9219. doi: 10.3390/ijms23169219 (PMC9408831; doi:10.3390/ijms23169219)
Supplement: Supplementary file 1 [file ijms-23-09219-s001.zip › Table S2.pdf]

Table S2. Detected founders in each model, including their ϱ estimate and standard error.

Clade	Probable Source	Sink (model)	number of samples (n)	ρ estimate	Standard error
L0a1	East Africa	North America	1	0	
L0a1a+64T	North Africa	Southwest Asia	3	4.666667	1.699673
L0a1a1	West Africa	Europe	1	0	
L0a1a1	East Africa	Southwest Asia	3	4.333333	1.452966
L0a1a2	East Africa	North America	3	1.333333	0.816497
L0a1a2+200G	West Africa	North America	3	3	1
L0a1a2+200G+12172G	Southern Africa	North America	1	0	
L0a1a2+200G+16362C	West Africa	North America	1	0	
L0a1a2+200G+16362C+150T	West Africa	North America	1	0	
L0a1a2+200G+16362C+236C	Southern Africa	North Africa	1	0	
L0a1a2+200G+16362C+236C	Southern Africa	North America	2	1.5	0.866025
L0a1a2+200G+8521G	West Africa	North America	1	0	
L0a1a3	West Africa	North America	1	0	
L0a1b+5563A	Southern Africa	South America	2	0	0
L0a1b+5563A+15099C+152C	Southern Africa	Southwest Asia	1	0	
L0a1b+5563A+7711C	Southern Africa	North America	3	0	0
L0a1b1	Southern Africa	North America	2	2.5	1.118034
L0a1b1+16093C+3644C	Southern Africa	North America	2	0	0
L0a1b1+16188G	Southern Africa	North Africa	2	1	0.707107
L0a1b1+16188G+16187T	East Africa	Southwest Asia	1	0	
L0a1b1a+3311T	Central Africa	North Africa	4	0	0
L0a1b1a+3311T+593C	North Africa	Southwest Asia	1	0	
L0a1b1a1+16188G+15481T	East Africa	Southwest Asia	1	0	
L0a1c1	East Africa	Southwest Asia	1	0	
L0a1d	East Africa	Southwest Asia	4	1	0.612372

L0a2a1a1	Southern Africa	North America	1	0	
L0a2a1b+16188G+16093C	Southern Africa	North America	1	0	
L0a2a2	East Africa	Southwest Asia	3	0	0
L0a2a2+16188G	East Africa	Southwest Asia	15	0.866667	0.371184
L0a2a2+16188G+150T	Southern Africa	North America	1	0	
L0a2a2+16188G+64T	East Africa	Southwest Asia	2	0	0
L0a2a2+16188G+64T	Southern Africa	South America	1	0	
L0a2a2a+16187T+16188T+7146G	East Africa	North Africa	1	0	
L0a2a2a+16274A	East Africa	Southwest Asia	1	0	
L0a2a2a+16274A+5201C	East Africa	North Africa	1	0	
L0a2a2a1	Southern Africa	North America	2	0	0
L0a2c+95C	East Africa	Southwest Asia	7	3.142857	0.92582
L0d3+152C	East Africa	Southwest Asia	2	5	1.581139
L0f+1822C+143A	East Africa	Southwest Asia	2	0	0
L0f2a1+11299C	East Africa	Southwest Asia	1	0	
L0f2b+7419A	East Africa	Southwest Asia	1	0	
L0k+198T+204C+15221A+3882A	East Africa	Southwest Asia	1	0	
L1b+16292T+11812G	West Africa	North America	1	0	
L1b1a	West Africa	North Africa	6	3.666667	0.971825
L1b1a	North Africa	Europe	10	4.4	1.029563
L1b1a	North Africa	Europe	10	4.4	1.029563
L1b1a	West Africa	North America	1	0	
L1b1a+1462A+11002G+198T	Europe (Western)	South America	2	0	0
L1b1a+146C	West Africa	North America	1	0	
L1b1a+16145A	West Africa	Europe	1	0	
L1b1a+16145A+3966T	West Africa	Europe	1	0	
L1b1a+16145A+3966T	West Africa	Europe	1	0	
L1b1a+16293C	West Africa	North Africa	2	0	0

L1b1a+16293C	West Africa	North America	2	1	0.707107
L1b1a+16293C+152C	West Africa	North America	1	0	
L1b1a+16293C+152C+73G	North Africa	North America	1	0	
L1b1a+16293C+3505G	West Africa	North America	1	0	
L1b1a+16293C+8251A	West Africa	Europe	2	0	0
L1b1a+16293C+8251A	West Africa	North America	1	0	
L1b1a+182T+189G+12696C	West Africa	North America	1	0	
L1b1a+185C	West Africa	Europe	8	3	0.829156
L1b1a+185C	West Africa	Europe	8	2.875	0.81968
L1b1a+189G	West Africa	Europe	7	4	1.087968
L1b1a+189G	West Africa	Europe	7	4	1.087968
L1b1a+189G	West Africa	North America	1	0	
L1b1a+189G+16293G+6446A	West Africa	Southwest Asia	1	0	
L1b1a+189G+16293G+6446A	West Africa	North America	2	1.5	0.866025
L1b1a10	West Africa	North America	2	0.5	0.5
L1b1a13	North Africa	Europe	1	0	
L1b1a14	West Africa	Europe	3	5.666667	1.795055
L1b1a14	West Africa	Europe	3	5.666667	1.795055
L1b1a14+16256T	West Africa	North America	1	0	
L1b1a15+152C	West Africa	North America	1	0	
L1b1a15+199C	East Africa	Southwest Asia	1	0	
L1b1a15+4080C	West Africa	North America	1	0	
L1b1a15a+146C	West Africa	North America	1	0	
L1b1a17+7859A	West Africa	North America	1	0	
L1b1a2	East Africa	Southwest Asia	3	0	0
L1b1a2a	East Africa	North Africa	1	0	
L1b1a2a+16126C	East Africa	Europe	1	0	
L1b1a2a+16126C	East Africa	Europe	1	0	

L1b1a3	West Africa	Europe	1	0	
L1b1a3	West Africa	Europe	1	0	
L1b1a3	West Africa	North America	6	0.333333	0.235702
L1b1a3+11020G	Southern Africa	North America	4	0	0
L1b1a3a	West Africa	Europe	1	0	
L1b1a3a	West Africa	Europe	1	0	
L1b1a3a	West Africa	Southwest Asia	2	0	0
L1b1a3a	West Africa	North America	9	0.666667	0.351364
L1b1a3a1	West Africa	North America	3	1	0.745356
L1b1a3b	West Africa	North America	2	2.5	1.118034
L1b1a4+16114A	West Africa	North America	2	2	1
L1b1a4+16114A+10172A	West Africa	North America	1	0	
L1b1a4+16114A+16234T	West Africa	North America	3	0.333333	0.333333
L1b1a4+3849A	West Africa	North America	1	0	
L1b1a4a+16114G	West Africa	North America	2	0.5	0.5
L1b1a5	North Africa	Europe	2	1	0.707107
L1b1a6	West Africa	North Africa	3	3.333333	1.054093
L1b1a6	North Africa	Europe	8	3	0.790569
L1b1a6	North Africa	Europe	8	3	0.790569
L1b1a6+3385G	West Africa	North America	2	0.5	0.5
L1b1a7	West Africa	North America	1	0	
L1b1a7+14053G	West Africa	North America	1	0	
L1b1a7+16293G	West Africa	North America	3	1	0.57735
L1b1a7+9855G	West Africa	North America	1	0	
L1b1a7a	West Africa	North America	1	0	
L1b1a7a+15224T	West Africa	Europe	1	0	
L1b1a7a+15224T	West Africa	Europe	1	0	
L1b1a7a+15224T	West Africa	North America	2	0.5	0.5

L1b1a8	North Africa	Europe	6	3.833333	1.190238
L1b1a8	North Africa	Europe	6	3.833333	1.190238
L1b1a8+16270T+5174A	North Africa	North America	1	0	
L1b2a+569T	West Africa	Europe	1	0	
L1c1+247A+16294T+16172C	West Africa	North America	1	0	
L1c1a	Central Africa	North America	1	0	
L1c1a+198T+93G+89C	Southern Africa	South America	1	0	
L1c1b+416A+4688C+10861C+508G	Southern Africa	South America	1	0	
L1c1b+416A+4688C+520T	WEST AFRICA	North America	1	0	
L1c1b+4688C+5876G	Southern Africa	North America	1	0	
L1c1d1+8657T+4167T	Central Africa	North America	1	0	
L1c2a1a+8251A+5585A	Southern Africa	North America	1	0	
L1c2a2+7070T+6990T	West Africa	North America	1	0	
L1c2b1a	Southern Africa	North America	1	0	
L1c2b1a'b+12477+1598A+6962A+200G+513A	East Africa	Southwest Asia	1	0	
L1c2b1b+513A	Southern Africa	North America	1	0	
L1c2b1b1+16093C	Southern Africa	Southern America	1	0	
L1c2b1c+5580C	West Africa	Europe	1	0	
L1c2b1c+5580C	West Africa	Europe	1	0	
L1c2b1c+5580C	West Africa	North America	1	0	
L1c2b1c+5580C16129A	West Africa	North America	1	0	
L1c2b2+182T+4722G	Southern Africa	North America	1	0	
L1c2b2+8621G	Southern Africa	Southwest Asia	3	4	1.490712
L1c3+195C	West Africa	North Africa	1	0	
L1c3+195C+6260A+16215G	West Africa	North America	1	0	
L1c3+195C+6260A+16215G+16093C	Central Africa	North America	1	0	
L1c3+195C+6260A+16215G+16145A	West Africa	North America	3	1.333333	0.942809
L1c3+195C+6260A+16215G+6791+16368C	Southern Africa	North America	2	0.5	0.5

L1c3a1a	West Africa	North Africa	2	0	0
L1c3a1a+16093C	West Africa	North America	1	0	
L1c3a1b+783G	Southern Africa	South America	1	0	
L1c3a1b+783G+16390A+16093C	West Africa	North America	1	0	
L1c3b1	West Africa	Southwest Asia	1	0	
L1c3b1+195C+513A	West Africa	Southern America	1	0	
L1c3b1a	Southern Africa	Southern America	3	1.666667	0.881917
L1c3b1a	Southern Africa	North America	2	0.5	0.5
L1c3b1b	West Africa	North America	1	0	
L1c3b2+16086C+16104T+14929T	West Africa	North America	11	0	0
L1c4b+9494G	Central Africa	North America	1	0	
L1c4b+9494G+146C	Central Africa	North America	1	0	
L1c5+7762A+195C	West Africa	North America	2	0.5	0.5
L2a1+143A+16189C+16192T	West Africa	Europe	4	4.25	1.520691
L2a1+16189C+143A	East Africa	North Africa	10	4.4	1.148913
L2a1+16189C+143A+11016A+5135T	East Africa	Southwest Asia	1	0	
L2a1+16189C+143A+16192T+16093C	North Africa	Southwest Asia	4	4	1.581139
L2a1+16189C+143A+16192T+16093C+5460A	North Africa	Europe	1	0	
L2a1+16189C+143A+16192T+16093C+5460A	North Africa	Europe	1	0	
L2a1+16189C+143A+16192T+16292T	North Africa	Southwest Asia	2	0	0
L2a1+16189C+143A+16309G+16192T	East Africa	Southwest Asia	7	4.285714	1.498298
L2a1+16189C+143A+16309G+16192T	Southwest Asia	North America	1	0	
L2a1+16189C+143A+16309G+16192T+4688C	North Africa	Southwest Asia	1	0	
L2a1+16189C+143A+16309G+16192T+8788T	Southwest Asia	North America	1	0	
L2a1+16189C+143A+16309G+6743C	East Africa	Southwest Asia	1	0	
L2a1a	West Africa	North Africa	1	0	
L2a1a	West Africa	North America	5	0.6	0.34641
L2a1a+15391T+4161T	North Africa	North America	1	0	

L2a1a+16189C+7337A	West Africa	North America	1	0	
L2a1a+16390A	West Africa	North America	1	0	
L2a1a1	West Africa	North America	8	1.25	0.612372
L2a1a1+16129A	West Africa	North America	1	0	
L2a1a2	West Africa	Europe	3	0.333333	0.333333
L2a1a2	East Africa	Southwest Asia	4	0.5	0.353553
L2a1a2	West Africa	North America	4	1	0.5
L2a1a2+16093C	West Africa	North America	1	0	
L2a1a2+16274A	West Africa	Europe	1	0	
L2a1a2+16274A	West Africa	Europe	1	0	
L2a1a2+7562G	West Africa	North America	1	0	
L2a1a2a	West Africa	North America	1	0	
L2a1a2a1	West Africa	North America	1	0	
L2a1a2a1a	West Africa	North America	5	1.2	0.565685
L2a1a2a1a+16092C	Southern Africa	North Africa	1	0	
L2a1a2a1a+16092C	Southern Africa	Southwest Asia	1	0	
L2a1a2a1a+16286T	West Africa	North Africa	2	3	1.224745
L2a1a2b	West Africa	North America	3	1.666667	1
L2a1a2c	West Africa	South America	1	0	
L2a1a2c+16286T	West Africa	North America	1	0	
L2a1a2c+16309G	West Africa	Southwest Asia	2	0	0
L2a1a2c+16309G	West Africa	North America	1	0	
L2a1a2c+207A	West Africa	North America	1	0	
L2a1a3	West Africa	Europe	3	0.333333	0.333333
L2a1a3	West Africa	Europe	3	0.333333	0.333333
L2a1a3+9007G+16093C+11399C	West Africa	North America	1	0	
L2a1a3a+16093C	West Africa	North America	1	0	
L2a1a3b	West Africa	North America	1	0	

L2a1b	West Africa	North America	1	0	
L2a1b+143A	West Africa	Europe	1	0	
L2a1b+143A	West Africa	Europe	1	0	
L2a1b+143A	West Africa	North America	3	1.333333	0.816497
L2a1b+143A+195C	West Africa	North America	1	0	
L2a1b+16192T	West Africa	North Africa	1	0	
L2a1b1+16192T+12007A+8485A	West Africa	North America	1	0	
L2a1b1+16192T+12130C	West Africa	North America	3	1.666667	1
L2a1b1a+16192T	East Africa	Southwest Asia	1	0	
L2a1c	West Africa	Europe	4	3	1.118034
L2a1c	West Africa	Europe	4	3	1.118034
L2a1c	West Africa	North America	5	2.6	0.72111
L2a1c+11614T	West Africa	North America	2	0	0
L2a1c+16086C	West Africa	North America	1	0	
L2a1c+16129A	West Africa	Europe	1	0	
L2a1c+16129A	West Africa	Europe	1	0	
L2a1c+16129A	East Africa	Southwest Asia	3	0	0
L2a1c+200G	West Africa	North America	1	0	
L2a1c+5147A+8222C	Southern Africa	North America	1	0	
L2a1c+6164T	West Africa	North Africa	1	0	
L2a1c1	West Africa	North Africa	1	0	
L2a1c1	West Africa	North America	1	0	
L2a1c1+6173T	West Africa	Europe	1	0	
L2a1c1+6173T	West Africa	Europe	1	0	
L2a1c1+6173T+12361G	Southern Africa	Europe	1	0	
L2a1c1+6173T+12361G	Southern Africa	Europe	1	0	
L2a1c1a	West Africa	South America	1	0	
L2a1c1a	East Africa	North America	1	0	

L2a1c2+7299G+644G	West Africa	North America	1	0	
L2a1c2a	West Africa	North America	1	0	
L2a1c3a	West Africa	Europe	3	4.333333	1.20185
L2a1c3a	West Africa	Europe	3	4.333333	1.20185
L2a1c3a	West Africa	Southwest Asia	3	0	0
L2a1c3a1	West Africa	North America	2	0	0
L2a1c3a1+13653T	West Africa	North America	2	0	0
L2a1c3b	West Africa	North America	1	0	
L2a1c3b1+16148T	West Africa	Europe	1	0	
L2a1c3b1+16148T	West Africa	Europe	1	0	
L2a1c4	West Africa	Europe	1	0	
L2a1c4	West Africa	Europe	1	0	
L2a1c4a	West Africa	North Africa	1	0	
L2a1c4a	West Africa	North America	1	0	
L2a1c4a1	West Africa	North America	4	1.25	0.559017
L2a1c5	West Africa	North America	6	1	0.527046
L2a1d	East Africa	North America	1	0	
L2a1d1	East Africa	Southwest Asia	2	2.5	1.118034
L2a1d1	East Africa	North America	4	0	0
L2a1e	West Africa	North America	2	4	1.414214
L2a1e1	West Africa	North America	2	0	0
L2a1e1+143A	West Africa	North America	4	2.75	1.089725
L2a1e1+143A+5244G	West Africa	North America	1	0	
L2a1f	West Africa	North America	6	1.5	0.5
L2a1f+16193T	West Africa	North America	1	0	
L2a1f1	West Africa	North America	5	1.2	0.489898
L2a1f1+12753G	West Africa	North America	1	0	
L2a1f1+12753G+6480A+8310C	East Africa	North America	1	0	

L2a1f1+14869A	West Africa	North America	3	0	0
L2a1f1+16192T	Southern Africa	North America	3	1.333333	0.666667
L2a1f1a	West Africa	North America	1	0	
L2a1f2+16093C	West Africa	North America	3	1.666667	0.745356
L2a1f3	East Africa	Southwest Asia	4	0.5	0.5
L2a1f3	West Africa	North America	1	0	
L2a1f3+16192T	West Africa	North America	12	0.666667	0.288675
L2a1f3+16192T+16223T	West Africa	Southwest Asia	1	0	
L2a1g+195C	Southern Africa	Southwest Asia	2	0	0
L2a1h	East Africa	Southwest Asia	1	0	
L2a1i1	West Africa	North America	1	0	
L2a1i1+13708A+8987C	West Africa	North America	1	0	
L2a1i1+143A	Southern Africa	North America	1	0	
L2a1j	East Africa	North Africa	1	0	
L2a1j	East Africa	Southwest Asia	1	0	
L2a1l	West Africa	Europe	1	0	
L2a1l	West Africa	Europe	1	0	
L2a1l1a	West Africa	North Africa	1	0	
L2a1l1a	West Africa	South America	1	0	
L2a1l1a	West Africa	North America	2	1.5	0.866025
L2a1l1a1	West Africa	North Africa	1	0	
L2a1l1a2+3345C	West Africa	North America	1	0	
L2a1l1b	West Africa	North America	2	1	0.707107
L2a1l1b+7388G	West Africa	North America	1	0	
L2a1l2+143A	West Africa	North Africa	1	0	
L2a1l2+16192T	West Africa	North America	1	0	
L2a1l2a	West Africa	Europe	10	0.5	0.3
L2a1l3	West Africa	North Africa	1	0	

L2a1m	West Africa	North America	1	0	
L2a1m1a	West Africa	Europe	1	0	
L2a1m1a	West Africa	North America	1	0	
L2a1m1a+16192T+153G+13326C	West Africa	North America	1	0	
L2a1n	West Africa	South America	1	0	
L2a1n	West Africa	North America	1	0	
L2a1n+16309G	West Africa	North America	1	0	
L2a1o+3372C	West Africa	Europe	1	0	
L2a1o+3372C	West Africa	Southwest Asia	1	0	
L2a5+3645T+511T+12967G+5246A+6893T	Southern Africa	Europe	2	0.5	0.5
L2a5+3645T+511T+12967G+5246A+6893T	Southern Africa	Europe	2	0.5	0.5
L2b1+16129A+3417T	West Africa	Southwest Asia	1	0	
L2b1a	West Africa	Europe	3	6	1.825742
L2b1a	West Africa	Europe	3	6	1.825742
L2b1a+207A	West Africa	North Africa	2	7.5	1.936492
L2b1a+7702A+8065A	West Africa	North America	1	0	
L2b1a2	West Africa	Southwest Asia	3	0.666667	0.666667
L2b1a2	West Africa	North America	1	0	
L2b1a2+204C	West Africa	North Africa	1	0	
L2b1a3	West Africa	North Africa	1	0	
L2b1a3	West Africa	Europe	1	0	
L2b1a3	West Africa	North America	8	0.25	0.176777
L2b1b+6629G+10586C	West Africa	North Africa	1	0	
L2b1b+6629G+16153A	West Africa	South America	1	0	
L2b2+183G	West Africa	North America	1	0	
L2b2+183G+16274A	West Africa	North America	1	0	
L2b2+3196A+15318T+513A	West Africa	North America	2	1	0.707107
L2b2a+16354T	West Africa	North America	4	1	0.612372

L2b3a	West Africa	EUrope	1	0	
L2b3a	West Africa	EUrope	1	0	
L2b3a	West Africa	North America	1	0	
L2b3b	West Africa	North America	1	0	
L2b3c+185C	West Africa	North America	1	0	
L2b3c+6293C+199C	West Africa	North Africa	1	0	
L2b3c+6293C+199C	West Africa	Southwest Asia	1	0	
L2c+13470G+146C	West Africa	North America	1	0	
L2c+16320T	West Africa	Europe	5	0.8	0.489898
L2c+16320T	West Africa	Europe	4	1	0.612372
L2c+198T+16177G	West Africa	Europe	2	0.5	0.5
L2c+198T+16177G	West Africa	Europe	2	0.5	0.5
L2c+198T+16177G	West Africa	North America	1	0	
L2c+198T+16177G+16311C	West Africa	Europe	1	0	
L2c+198T+16177G+16311C	West Africa	Europe	1	0	
L2c+198T+8425G	West Africa	North America	1	0	
L2c+6150A	West Africa	North America	1	0	
L2c+6150A+64T	West Africa	North America	3	1	0.57735
L2c+95C+14750T	West Africa	North America	1	0	
L2c+95C+14750T+8269A	West Africa	North America	1	0	
L2c1	West Africa	North America	2	1.5	0.866025
L2c1+3745A	West Africa	North America	1	0	
L2c1a+13708A	West Africa	North Africa	1	0	
L2c1a+13708A+3645C	Morocco	Europe	1	0	
L2c1a+13708A+3645C	Morocco	Europe	1	0	
L2c2	West Africa	Southwest Asia	1	0	
L2c2	West Africa	North America	2	1	0.707107
L2c2+14182C	West Africa	North America	1	0	

L2c2+16093C	West Africa	North America	1	0	
L2c2a	West Africa	North America	3	1	0.57735
L2c2a+198T	West Africa	North America	1	0	
L2c2a1	West Africa	North America	1	0	
L2c2b1b	Southern Africa	Europe	1	0	
L2c2b1b	Southern Africa	North America	1	0	
L2c3+93G+146C	West Africa	North Africa	1	0	
L2c4	West Africa	North America	1	0	
L2c5+13135A	West Africa	North America	1	0	
L2c5+14374C	West Africa	Europe	1	0	
L2c5+14374C	West Africa	Europe	1	0	
L2c5+5351G	West Africa	North America	1	0	
L2d+16129A	West Africa	North America	1	0	
L2d+16129A+11253C	West Africa	South America	1	0	
L2d+16129A+16093C	West Africa	North Africa	1	0	
L2d+16129A+199C	North Africa	North America	1	0	
L2d1+2223G	West Africa	North America	1	0	
L2d1a+16311C	East Africa	Southwest Asia	1	0	
L2d1a+16311C	Southern Africa	North America	1	0	
L2d1a+16311C+16093C+3197C	East Africa	Southwest Asia	2	0	0
L2e1a+183G	West Africa	North America	1	0	
L3b1a	East Africa	North Africa	2	5	1.581139
L3b1a	East Africa	North Africa	3	5.333333	1.763834
L3b1a	East Africa	North America	1	0	
L3b1a+11002G	West Africa	North Africa	1	0	
L3b1a+11002G+151T+2071C	West Africa	North Africa	1	0	
L3b1a+15311G+15758G	West Africa	North America	1	0	
L3b1a+15311G+8347G+372G	West Africa	North America	1	0	

L3b1a+15311G+8347G+372G+152C	West Africa	North Africa	1	0	
L3b1a+15434T+8772C	West Africa	North America	1	0	
L3b1a+15434T+8772C+16301T	West Africa	North America	1	0	
L3b1a+16093C	West Africa	South America	1	0	
L3b1a+16093C	West Africa	South America	1	0	
L3b1a+16311C+2775G	Central Africa	North Africa	1	0	
L3b1a+9055A+3866C+14781T	West Africa	North America	1	0	
L3b1a10	West Africa	North America	1	0	
L3b1a10+14182T	West Africa	North America	1	0	
L3b1a11	Southern Africa	Southwest Asia	1	0	
L3b1a11	Southern Africa	North America	2	0	0
L3b1a1a+11800G	East Africa	Europe	3	1.333333	0.666667
L3b1a1a+11800G	East Africa	Southwest Asia	3	0.333333	0.333333
L3b1a1a+11800G	East Africa	North America	3	0.666667	0.471405
L3b1a1a+11800G+16093C	East Africa	Europe	1	0	
L3b1a1a+11800G+16093C	East Africa	Southwest Asia	3	1.333333	0.666667
L3b1a2	East Africa	Southwest Asia	4	5.25	1.299038
L3b1a3	East Africa	North Africa	3	2.666667	1.154701
L3b1a3	North Africa	North America	2	0	0
L3b1a4	West Africa	North Africa	1	0	
L3b1a4	West Africa	Southwest Asia	1	0	
L3b1a4	West Africa	North America	1	0	
L3b1a4+16311C	West Africa	North America	1	0	
L3b1a5	West Africa	North America	1	0	
L3b1a5+152C	West Africa	North Africa	1	0	
L3b1a6	West Africa	North America	1	0	
L3b1a6+13658T	West Africa	North America	1	0	
L3b1a7	West Africa	North Africa	2	0	0

L3b1a7+5211T	West Africa	North America	1	0	
L3b1a9	West Africa	North Africa	1	0	
L3b1a9a	West Africa	North Africa	1	0	
L3b1b+9079G+15664T	West Africa	North Africa	1	0	
L3b1b+9079G+15664T	Southwest Asia	Europe	1	0	
L3b1b+9079G+15664T	West Africa	Southwest Asia	1	0	
L3b1b+9079G+6891G	Southern Africa	North America	1	0	
L3b2+9299G	West Africa	North America	2	0	0
L3b2a	West Africa	North Africa	1	0	
L3b2a+16311C	West Africa	North America	1	0	
L3b2b	West Africa	North America	1	0	
L3b3	West Africa	North America	2	3	1.224745
L3b3+185A	West Africa	Europe	1	0	
L3b3+185A	West Africa	North America	2	1	0.707107
L3c	East Africa	Southwest Asia	1	0	
L3d	West Africa	Europe	1	0	
L3d1'2'3'4'5'6+151T	West Africa	North America	2	1	0.707107
L3d1'2'3'4'5'6+3504C	West Africa	North America	2	1.5	0.866025
L3d1a+4048A	East Africa	Southwest Asia	1	0	
L3d1a1+150T	Southern Africa	North America	1	0	
L3d1a1a	Southern Africa	North America	1	0	
L3d1a1a+3666A	East Africa	Southwest Asia	1	0	
L3d1a1a1	Southern Africa	North America	2	1.5	0.866025
L3d1a1a1+7765G	East Africa	North America	1	0	
L3d1a2+3796G+10463C	Southern Africa	North America	1	0	
L3d1b1	West Africa	Europe	5	3	1.148913
L3d1b1+150T	West Africa	Europe	1	0	
L3d1b1+150T	West Africa	Europe	1	0	

L3d1b1+150T+3459T+16093C	West Africa	Europe	1	0	
L3d1b1+150T+3459T+16093C	West Africa	Europe	1	0	
L3d1b1b	West Africa	Europe	1	0	
L3d1b2+4553C	West Africa	North America	2	1	0.707107
L3d1b3+146C+10694T	West Africa	North America	1	0	
L3d1b3+146C+16256T+14040A	West Africa	Europe	3	1	0.745356
L3d1b3+146C+16256T+14040A	West Africa	Europe	3	1	0.745356
L3d1b3+146C+16256T+14040A+12616C+11239G	West Africa	North America	1	0	
L3d1b3+146C+16256T+6446A	West Africa	North America	1	0	
L3d1c+9708C	West Africa	North Africa	1	0	
L3d1c1	West Africa	North Africa	1	0	
L3d1c1	West Africa	North America	1	0	
L3d1d+8709T+13632G	Southern Africa	North America	1	0	
L3d2b+15115C	West Africa	Southwest Asia	1	0	
L3d2b+151T+263G+850C	West Africa	North America	1	0	
L3d2b+2163G	West Africa	North America	1	0	
L3d3a	West Africa	Europe	1	0	
L3d3a1+16304C	Southern Africa	North America	1	0	
L3d3a1a	Southern Africa	Southwest Asia	1	0	
L3d3b+15752C	East Africa	Southwest Asia	1	0	
L3d3b+15752C+6722A	Southern Africa	Europe	1	0	
L3d3b+15752C+6722A	Southern Africa	Europe	1	0	
L3d4	West Africa	North Africa	3	5	1.666667
L3d4	West Africa	North America	3	0	0
L3d4a+16124C	East Africa	Southwest Asia	1	0	
L3d6	West Africa	North America	1	0	
L3e1	West Africa	Europe	1	0	
L3e1	West Africa	Europe	1	0	

L3e1	West Africa	Southwest Asia	2	0	0
L3e1	West Africa	North America	1	0	
L3e1+189G	Southwest Asia	North America	1	0	
L3e1a+152C+16189C	West Africa	South America	1	0	
L3e1a1a+16311C	East Africa	Southwest Asia	1	0	
L3e1a1a+16311C	Southern Africa	South America	1	0	
L3e1a1a+16311C	Southern Africa	North America	1	0	
L3e1a1a+16311C+8407T	Southern Africa	South America	1	0	
L3e1a2+4173G	East Africa	Southwest Asia	1	0	
L3e1a3a	Southern Africa	North America	2	0.5	0.5
L3e1a3a+195C	East Africa	Southwest Asia	1	0	
L3e1a3a+195C+207A	East Africa	Europe	1	0	
L3e1b1	Central Africa	Southwest Asia	4	3	1.224745
L3e1c	Central Africa	Southwest Asia	1	0	
L3e1d1	Southern Africa	North America	1	0	
L3e1e	East Africa	North America	2	4.5	1.5
L3e1e+16309G	West Africa	North America	1	0	
L3e1e+73G	East Africa	North America	8	0.5	0.25
L3e1f1a	Southern Africa	North America	2	0	0
L3e2a	West Africa	North Africa	3	3.666667	1.527525
L3e2a	West Africa	North America	1	0	
L3e2a+189A	West Africa	North America	1	0	
L3e2a+9196A	West Africa	Europe	1	0	
L3e2a1a	West Africa	North America	3	0.666667	0.471405
L3e2a1b	West Africa	North America	3	1	0.57735
L3e2a1b+6167C	West Africa	North America	2	0	0
L3e2a1b1+499A	West Africa	North Africa	2	0	0
L3e2a1b1+499A	West Africa	South America	1	0	

L3e2a1b1+499A	West Africa	North America	10	0.9	0.387298
L3e2a1b1+499A+14323A	West Africa	North America	1	0	
L3e2a1b1+499A+16311C	West Africa	North America	1	0	
L3e2a1b3	West Africa	South America	1	0	
L3e2a1b3	West Africa	North America	2	1.5	0.866025
L3e2a2+709A	West Africa	Southwest Asia	1	0	
L3e2b	West Africa	North Africa	3	6.333333	1.452966
L3e2b	West Africa	Europe	1	0	
L3e2b	West Africa	Europe	1	0	
L3e2b	West Africa	North America	1	0	
L3e2b+15253G	Southern Africa	North America	1	0	
L3e2b+15287C	Southern Africa	North America	1	0	
L3e2b+152C	West Africa	North America	15	0.8	0.339935
L3e2b+152C+146C	West Africa	North America	1	0	
L3e2b+152C+3606G+647G	Southern Africa	North America	1	0	
L3e2b+152C+3606G+647G+4092A	WEST AFRICA	North America	1	0	
L3e2b+15784C	West Africa	Europe	2	0.5	0.5
L3e2b+1676G	North Africa	Europe	1	0	
L3e2b+1676G	North Africa	Europe	1	0	
L3e2b+5186G	Southern Africa	North America	1	0	
L3e2b+769A	West Africa	Europe	1	0	
L3e2b+769A	West Africa	Europe	1	0	
L3e2b+769A+146A	West Africa	North America	1	0	
L3e2b+769A+4164G	Southern Africa	North America	1	0	
L3e2b+769A+4164G	East Africa	North America	1	0	
L3e2b1	West Africa	North Africa	2	0	0
L3e2b1	West Africa	Europe	1	0	
L3e2b1	West Africa	Europe	1	0	

L3e2b1+961C	West Africa	North America	1	0	
L3e2b1+961C+4496T	West Africa	North Africa	1	0	
L3e2b1a1	West Africa	North America	1	0	
L3e2b1a1+11722C	West Africa	North America	1	0	
L3e2b1a1+11722C+5210T	West Africa	North America	4	2.25	1.145644
L3e2b1a2	West Africa	North Africa	1	0	
L3e2b1a2+282C	West Africa	Europe	1	0	
L3e2b1a2+282C	West Africa	Europe	1	0	
L3e2b2	West Africa	Southwest Asia	2	0	0
L3e2b2+4560A+1694C	West Africa	North Africa	1	0	
L3e2b3	West Africa	North America	3	1	0.57735
L3e2b4+11440A	West Africa	North America	1	0	
L3e2b4+8206A	West Africa	North America	1	0	
L3e2b8+8730G	West Africa	North America	3	0	0
L3e3a	East Africa	Southwest Asia	6	1	0.707107
L3e3a	Southern Africa	North America	2	0.5	0.5
L3e3a+574C	East Africa	Southwest Asia	1	0	
L3e3b	West Africa	North America	5	0.4	0.282843
L3e3b+16093C	West Africa	North America	2	1	0.707107
L3e3b+16093C+16148T	West Africa	North America	1	0	
L3e3b+189G+16093C	West Africa	North America	1	0	
L3e3b1	Southern Africa	South America	1	0	
L3e3b1	Southern Africa	North America	1	0	
L3e3b1+16311C	Southern Africa	South America	1	0	
L3e3b1+16325C	Southern Africa	North America	1	0	
L3e3b3+195C	West Africa	North America	2	2	1
L3e4	West Africa	Southwest Asia	1	0	
L3e4a	West Africa	North America	1	0	

L3e4a+493G	Southern Africa	North America	1	0	
L3e4a+709A	West Africa	North America	1	0	
L3e4a+8688C	West Africa	Europe	1	0	
L3e4a+8688C	West Africa	Europe	1	0	
L3e4a+8688C	West Africa	North America	1	0	
L3e5	Central Africa	North Africa	3	4	1.154701
L3e5	North Africa	Europe	1	0	
L3e5	North Africa	Europe	1	0	
L3e5a	Central Africa	North Africa	3	3	1.20185
L3e5a	North Africa	Europe	2	1	0.707107
L3e5a	North Africa	Europe	2	1	0.707107
L3e5a	North Africa	Southwest Asia	1	0	
L3e5a+2833G	Central Africa	North Africa	2	3	1.224745
L3e5d	Central Africa	North Africa	1	0	
L3f+152C+16311C	East Africa	North Africa	1	0	
L3f+152C+709A	East Africa	Southwest Asia	1	0	
L3f1b	West Africa	Europe	7	2.857143	0.92582
L3f1b	West Africa	Europe	7	2.571429	0.989743
L3f1b	West Africa	North America	1	0	
L3f1b+16292T	West Africa	North Africa	2	2.5	1.118034
L3f1b+16292T	West Africa	Europe	1	0	
L3f1b+16292T	East Africa	Southwest Asia	11	4.090909	0.922627
L3f1b+16292T+13879C+10649C	West Africa	North Africa	1	0	
L3f1b+16292T+13879C+215G	West Africa	North America	2	0	0
L3f1b+16292T+150T+207A	West Africa	North America	1	0	
L3f1b+16292T+150T+207A+10670T	West Africa	South America	1	0	
L3f1b+16292T+150T+207A+10670T	West Africa	North America	2	3	1.224745
L3f1b+16292T+16295T	West Africa	Europe	1	0	

L3f1b+16292T+16295T	West Africa	Europe	1	0	
L3f1b1a	West Africa	North America	13	0.538462	0.27735
L3f1b1a+16189	West Africa	North America	2	3	1.224745
L3f1b1a+2080C	West Africa	Europe	1	0	
L3f1b1a+272G	Southern Africa	North America	1	0	
L3f1b1a+272G+16295T	Southern Africa	North America	1	0	
L3f1b4	Southern Africa	North America	2	0	0
L3f1b4+16292T	Southern Africa	North America	2	2	1
L3f1b4a1	East Africa	Southwest Asia	1	0	
L3f1b4a1	Southern Africa	North America	1	0	
L3f1b4c	West Africa	North America	2	1.5	0.866025
L3f2a1a+13194A	East Africa	Southwest Asia	2	0.5	0.5
L3f2b	Southern Africa	North Africa	1	0	
L3h1a2+16270T	East Africa	Southwest Asia	3	7.333333	1.563472
L3h1a2a+3892G	East Africa	North Africa	1	0	
L3h1a2a1	East Africa	Southwest Asia	1	0	
L3h1a2a1+16192T+152C	East Africa	Southwest Asia	1	0	
L3h1a2a1+16192T+5108C+723G	East Africa	Southwest Asia	1	0	
L3h1b1	East Africa	North Africa	1	0	
L3h1b1a+14950T+513A	East Africa	Southwest Asia	1	0	
L3h1b1a+14950T+513A+9776T	East Africa	Southwest Asia	1	0	
L3h1b1a+2831A+16215G+9827T+11563T	West Africa	Europe	1	0	
L3h1b1a+2831A+16215G+9827T+11563T	West Africa	Europe	1	0	
L3h1b1a+2831A+16215G+9827T+11563T+16126C	West Africa	North America	1	0	
L3h1b1a+6359G	Central Africa	North Africa	1	0	
L3h1b1a+6359G+152C	North Africa	Europe	1	0	
L3h1b1a+6359G+152C	North Africa	Europe	1	0	
L3h1b2	West Africa	Southwest Asia	1	0	

L3h1b2+606G+151T+13194A+152C	Southern Africa	North Africa	1	0	
L3h1b2+606G+151T+13194A+7424G	West Africa	North Africa	2	5.5	1.658312
L3h2+7805A	East Africa	Southwest Asia	6	3.833333	1.142609
L3i1a+11914A+15743A	East Africa	Southwest Asia	2	2.5	1.118034
L3i1b	East Africa	Southwest Asia	3	6	1.885618
L3i2+189G+200G+204C	East Africa	Southwest Asia	1	0	
L3i2+189G+5441G+12630A	East Africa	Southwest Asia	3	3	1
L3k	East Africa	North Africa	2	10.5	2.291288
L3k	North Africa	North America	1	0	
L3k1+152C	West Africa	North America	2	0	0
L3k1+152C+14013G	East Africa	Southwest Asia	1	0	
L3x1	East Africa	Southwest Asia	1	0	
L3x1+152C	East Africa	Southwest Asia	1	0	
L3x1a1	East Africa	Southwest Asia	2	8.5	2.061553
L3x1b+204C	East Africa	Southwest Asia	4	5.5	1.541104
L3x1b1+199C	East Africa	Southwest Asia	1	0	
L3x2a+11063T	East Africa	Southwest Asia	2	0.5	0.5
L3x2a1a	East Africa	Southwest Asia	3	3.666667	1.105542
L3x2b	East Africa	North Africa	2	6.5	1.802776
L3x2b	North Africa	Europe	4	2.5	0.790569
L3x2b	North Africa	Europe	4	2.5	0.790569
L4a1a	East Africa	Southwest Asia	1	0	
L4a1a+16270	East Africa	North Africa	1	0	
L4a1a+8631G+189G	East Africa	Southwest Asia	1	0	
L4a1a+8631G+189G+150T+12280G	East Africa	Southwest Asia	1	0	
L4a2	East Africa	Southwest Asia	4	6	1.936492
L4b+5460A+73G	East Africa	Europe	1	0	
L4b1a+709A+513A+496T+16365T	West Africa	Southwest Asia	1	0	

L4b2+152C+8227C+150T+12354C+200G	West Africa	Europe	1	0	
L4b2+152C+8227C+150T+12354C+200G	West Africa	Europe	1	0	
L4b2a+195C+152C+7241G	East Africa	Southwest Asia	3	0	0
L4b2a+6260A+1694C+16172C+391C+5483G+10325A	East Africa	Southwest Asia	1	0	
L4b2a+6260A+1694C+16172C+5048C	East Africa	Southwest Asia	2	1.5	0.866025
L4b2a+6260A+1694C+16172C+6956C+16287T	East Africa	Southwest Asia	1	0	
L4b2a+6260A+1694C+2483C+257G	East Africa	Southwest Asia	3	4	1.154701
L5a1a+5004C	East Africa	Southwest Asia	3	3.333333	1.414214
L5a1b+204C+13224C	East Africa	Southwest Asia	1	0	
L5b1a	East Africa	Southwest Asia	2	5.5	1.658312
L5b1b	East Africa	North Africa	1	0	
L6a+207A+195C	East Africa	Southwest Asia	3	0	0
L6a+207A+7145T	East Africa	North Africa	1	0	
L6b	East Africa	Southwest Asia	2	5.5	1.658312
